# Estimated economic burden of genital herpes and HIV attributable to herpes simplex virus type 2 infections in 90 low- and middle-income countries: A modeling study

**DOI:** 10.1371/journal.pmed.1003938

**Published:** 2022-12-15

**Authors:** Sachin Silva, Houssein H. Ayoub, Christine Johnston, Rifat Atun, Laith J. Abu-Raddad

**Affiliations:** 1 Harvard TH Chan School of Public Health, Harvard University Boston, Massachusetts, United States of America; 2 University of California, San Francisco, Institute for Global Health Sciences, San Francisco, California, United States of America; 3 Mathematics Program, Department of Mathematics, Statistics, and Physics, College of Arts and Sciences, Qatar University, Doha, Qatar; 4 Department of Medicine, University of Washington, Seattle, Washington, United States of America; 5 Vaccine and Infectious Diseases Division, Fred Hutchinson Cancer Research Center, Seattle, Washington, United States of America; 6 Infectious Diseases Epidemiology Group, Weill Cornell Medicine–Qatar, Doha, Qatar; 7 World Health Organization Collaborating Centre for Disease Epidemiology Analytics on HIV/AIDS, Sexually Transmitted Infections, and Viral Hepatitis, Weill Cornell Medicine–Qatar, Doha, Qatar; 8 Department of Population Health Sciences, Weill Cornell Medicine, Cornell University, New York, New York, United States of America

## Abstract

**Background:**

Economic losses due to herpes simplex infections in low- and middle-income countries (LMICs) are unknown. We estimated economic and quality-of-life losses due to genital herpes in 2019, in 90 LMICs, and from 2020 to 2030 in 45 countries in the World Health Organization (WHO) Africa. We additionally estimated economic losses due to human immunodeficiency virus (HIV) attributable to herpes simplex virus type 2 (HSV-2) infections.

**Methods and findings:**

We estimated genital herpes-related spending on treatment, wage losses due to absenteeism, and reductions in quality of life, for individuals aged 15 to 49 years, living with genital herpes. Had HSV-2 had contributed to the transmission of HIV, we estimated the share of antiretroviral treatment costs and HIV-related wage losses in 2019 that can be attributed to incident and prevalent HSV-2 infections in 2018. For the former, we used estimates of HSV-2 incidence and prevalence from the global burden of disease (GBD) study. For the latter, we calculated population attributable fractions (PAFs), using the classic (Levin’s) epidemiological formula for polytomous exposures, with relative risks (RRs) reported in literature. To extend estimates from 2020 to 2030, we modeled the transmission of HSV-2 in 45 African countries using a deterministic compartmental mathematical model, structured by age, sex, and sexual activity, which was fitted to seroprevalence gathered from a systematic review and meta-regression analysis.

In the 90 LMICs, genital herpes contributed to US$813.5 million in treatment and productivity losses in 2019 (range: US$674.4 to US$952.2 million). Given observed care-seeking and absenteeism, losses are in the range of US$29.0 billion (US$25.6 billion to US$34.5 billion). Quality-of-life losses in the amount of 61.7 million quality-adjusted life years (QALYs) are also possible (50.4 million to 74.2 million). The mean annual cost of treatment and wage losses per infection is US$183.00 (95% CI: US$153.60 to US$212.55); the mean annual cost of quality-of-life losses is US$343.27 (95% CI: 272.41 to 414.14). If HSV-2 has fueled the transmission of HIV, then seroprevalent HSV-2 cases in 2018 can account for 33.2% of the incident HIV infections in 2019, with an associated antiretroviral therapy (ART) cost of US$186.3 million (range: US$163.6 to US$209.5 million) and 28.6% of HIV-related wage losses (US$21.9 million; range: US$19.2 to US$27.4 million). In the WHO Africa region, the 3.9 million seroprevalent genital herpes cases from 2020 to 2030 contributed to US$700.2 million in treatment and productivity losses. Additionally, quality-of-life losses in the range of 88 million to 871 million QALYs are also possible. If HSV-2 has contributed to the transmission of HIV, then in 2020, the PAF of HIV due to prevalent HSV-2 will be 32.8% (95% CI: 26.7% to 29.9%) and due to incident infections will be 4.2% (95% CI: 2.6% to 3.4%). The PAF due to prevalent infections will decline to 31.0% by 2030 and incident infections to 3.6%. Though we have accounted for the uncertainty in the epidemiological and economic parameter values via the sensitivity analysis, our estimates still undervalue losses due to limiting to the 15- to 49-year-old population.

**Conclusions:**

Economic losses due to genital herpes in LMICs can be large, especially when considering the lifelong nature of the disease. Quality-of-life losses outweigh spending on treatment and reductions in productivity. If HSV-2 has contributed to the spread of HIV in LMICs, then nearly one third of antiretroviral costs and HIV-related wage losses can be attributed to HSV-2. Given the magnitude of the combined losses, a vaccine against HSV-2 must be a global priority.

## Abstract 2

In a modelling study, Dr Sachin Silva and colleagues estimate the economic burden of genital herpes and HIV attributable to herpes simplex virus type-2 infections in 90 low and middle income countries.

## Author summary

**Why was this study done?:**

▪ Economic losses due to herpes simplex virus type 2 (HSV-2) infections in low- and middle-income countries (LMICs) are unknown.
▪ Quality-of-life losses due to genital herpes in LMICs are also unknown, both in terms of their magnitude and their economic value.
▪ If HSV-2 has indeed fueled the spread of human immunodeficiency virus (HIV), as has been conjectured, then the share of HIV-related spending in LMICs that are attributable to HSV-2 infections are also unknown.

**What did the researchers do and find?:**

▪ We estimated direct and indirect economic losses and quality-of-life losses due to genital herpes in individuals between the aged of 15 to 49 living in 90 LMICs in 2019.
▪ We also estimated these costs in 45 countries in the World Health Organization (WHO) Africa region from 2020 to 2030, by constructing a mathematical model of HSV-2 dynamics to predict HSV-2 incidence and prevalence.
▪ We supplemented the 2019 estimates with the share of HIV-related spending in 2019 attributable HSV-2 infections in 2018.
▪ For the 90 LMICs, we found that treatment costs and productivity losses in 2019 are in the range of US$674.4 and US$952.2 million.
▪ Quality-of-life losses are in the range of 50.4 million to 74.2 million quality-adjusted life years (QALYs).
▪ Additionally, seroprevalent HSV-2 cases in 2019 can account for 33.2% of antiretroviral therapy (ART) costs for incident HIV cases and 28.6% of HIV-related wage.

**What do these findings mean?:**

▪ Economic losses due to genital herpes can be substantial, especially when considering the lifelong nature of the disease.
▪ The quality-of-life losses due to genital herpes outweigh spending on treatment and reduced productivity (on the order of US$212 billion).
▪ If HSV-2 has indeed contributed to the transmission of HIV, then nearly one third of antiretroviral costs and HIV-related wage losses add to these costs.
▪ Given the magnitude of the above losses in LMICs, a vaccine against HSV-2 should be a global priority.

## Introduction

More than 13% of the world’s population aged 15 to 49 years, nearly 491 million individuals, were living with a herpes simplex virus type 2 (HSV-2) infection in 2016 [1]. The 2 viral subtypes of HSV, HSV-1 and HSV-2, cause a lifelong infection that results in mucocutaneous and systemic diseases ranging from oral and genital herpes to aseptic and lymphocytic meningitis and ocular keratitis [2]. Intrapartum exposure of a newborn to HSV can cause neonatal herpes, a rare but lethal condition [3]. HSV-2 infection is also believed to increase the risk of acquisition and transmission of the human immunodeficiency virus (HIV) by several-fold [4]. HSV’s low threshold for transmission [5], high rates of seroprevalence, and broad spectrum of clinical diseases and psychosocial and psychosexual sequalae [6,7], suggest that it may underlie substantial economic and quality-of-life losses. If HSV-2 has indeed fueled the global spread of HIV as has been conjectured [8], then HSV-2 can possibly account for a large share of HIV-related losses as well. Yet, the economic and quality-of-life burden due to this seemingly ubiquitous infection, particularly in low- and middle-income countries (LMICs), is still unknown.

Estimates of economic losses due to HSV-2 diseases from high-income countries show wide variation. They also do not account for costs beyond treatment and productivity [9–12]. The failure to account for psychosocial sequalae in HSV-2 economic evaluations is particularly problematic as a spectrum of such sequalae is attributed to HSV-2 diseases [6,13,14]. Due to the lifelong nature of the infection, effects are protracted, often leading to lifelong degradation in quality of life. In regions such as sub-Saharan Africa where as much as 80% of people living with HIV are seropositive for HSV-2 [15,16] the dual burden compounds clinical disease and psychosocial sequalae and complicates treatment. Even though direct causality from HSV-2 to HIV is yet to be established beyond doubt [17,18], an estimate of the share of the economic burden of HIV that can be averted by targeting HSV-2 is still useful, more so now as efforts to contain the Severe Acute Respiratory Syndrome Coronavirus 2 (SARS-CoV-2) virus (Coronavirus Disease 2019 (COVID-19)) have had adverse impacts on HIV control programs [19]. To catalyze investment in a vaccine that can prevent the acquisition or reactivation of HSV, its economic value in terms of the averted economic burden, also needs to be quantified. An estimate of economic gains can also signal to vaccine manufactures of the size of markets in LMICs that have been traditionally ignored in investment evaluations due to a perceived lack of purchasing power.

We present, to our knowledge, the first estimates of the economic losses due to genital herpes [20] and HIV-attributable-to-HSV-2 infections in 90 LMICs. We attempt to answer 3 questions with our analysis: how large are the direct and indirect economic losses; how large are the quality-of-life losses; in which geographies are the losses most concentrated in? We focus on HSV-2 and not on HSV-1 because LMICs bear a disproportionately higher genital herpes disease burden due to HSV-2 infections, compared to high-income countries [21]. We focus on genital herpes because given its high prevalence, disability, [20] and chronicity, it is likely to be the major contributor to HSV-2-related economic losses.

## Methods

We estimated direct and indirect economic losses and quality-of-life losses that accrued to individuals between the ages of 15 to 49 years with genital herpes and HIV-attributable-to-HSV-2 infections, living in 90 LMICS in 2019 and in 45 countries in the World Health Organization (WHO) Africa region from 2020 to 2030, respectively. For the former, we used estimates of HSV-2 incidence and prevalence from global burden of disease (GBD). For the latter, we modeled the transmission of HSV-2 in the 45 LMICs using a deterministic compartmental mathematical model, structured by sex, age (20 age groups, 5-year age bands), sexual risk group (5 sexual risk groups), HSV-2 status (uninfected, infected asymptomatic, infected symptomatic), stage of infection (primary, latent, infection reactivation), and time since acquiring the infection (<1 year, 1 to 9 years, and >10 years).

The model adapted and extended a recent model characterizing the HSV-2 epidemic in the United States from 1950 to 2050 [22]. The model was parameterized for HSV-2 natural history and epidemiology [22,23] and then calibrated by fitting to HSV-2 seroprevalence data by sex, and an overall seroprevalence decline by 2% [24] (S1 Appendix, section 3.A).

To describe the natural history of genital herpes, we constructed a model with 3 natural history states—a symptomatic first episode, symptomatic recurrent episodes (up to 5 per year), and symptomatic frequent recurrences (up to 7 per year). We assumed that 21.0% of individuals with recently acquired infections would experience a first episode (range: 12.7% to 32.8%) [25] and that 83.8% of individuals with established infections would have 1 or more genital herpes recurrences in a year (range: 69.5% to 92.2%) [26,27]. We assumed that of those, 74.83% would have recurrences within the mean (range: 56.12%, 93.54%) and 25.17% would have recurrences that exceed the mean (frequent recurrences) (range: 18.88%, 31.46%) [26–28] (Table 1 and S1 Appendix, section 3.B).

**Table 1:** HSV-2 parameter values and assumptions.

Parameter	Value	Source
Percentage of individuals with recently acquired infection who experience a first episode	21.0% (12.7%, 32.8%)	Langenberg and colleagues^11^
Percentage of individuals with established infection who have 1 or more genital herpes recurrences in a year	83.8% (69.5%, 92.2%),	Beneditti^12^ and Corey and colleagues^13^
Percentage of individuals with established infection who have 1 or more genital herpes recurrences in a year	*33% (24.75%, 41.25%)	Beneditti^12^ and Corey and colleagues^13^
Percentage of genital herpes recurrences within the mean	74.83% (56.12%, 93.54%)	Beneditti and colleagues^12^, Corey and colleagues^13^, Patel and colleagues^18^
Percentage of genital herpes recurrences that exceed the mean	25.17% (18.88%, 31.46%)	Beneditti and colleagues^12^, Corey and colleagues^13^, Patel and colleagues^18^
Mean number of genital herpes days per person with recently acquired infection and experiencing a first episode in the absence of antiviral therapy	20.5 days (18.3 days, 22.8 days),	Beneditti^12^ and Corey and colleagues^13^
Mean annual number of recurrences in those with recently acquired infection	4.6 (95% CI 3.9, 5.5)	Corey and colleagues^14^
Duration of a recurrent episode in a recently acquired infection	8.5 days (95% CI 7.5, 9.5)	Corey and colleagues^14^
Duration of a recurrent episode in an established infection (1–9 years)	7.2 days (95% CI 1, 23)	Corey and colleagues^14^
Duration of a recurrent episode in an established infection (>10 years) in the absence of antiviral therapy	6.5 days (95% CI 1, 24)	Corey and colleagues^14^
Percent of recently acquired and established infections where recurrences are within the mean	*74.83% (56.12%, 93.54%)	Patel and colleagues^14^
Percent of recently acquired and established infections where recurrences exceed the mean (frequent recurrences)	*25.17% (18.88%, 31.46%)	Patel and colleagues
Average number of workdays lost in preceding 3 months due to recurrences within the mean	0.4 days (0–21)	Patel^12^
Average number of workdays lost in preceding 3 months due to recurrences exceeding the mean	0.8 days (0–21)	Patel^12^

For estimates for the 90 LMICS for 2019 and for the 45 LMICs in the WHO Africa region, from 2020 to 2030. Values gathered from a rapid review of literature (appendix sections 3B, 4C, 7).

*Where uncertainty ranges were not available from literature, a +/- 25% was assumed.

### Losses due to genital herpes

To estimate direct economic losses, which included the costs of medicines and diagnostics, we determined whether countries employed syndromic or etiologic diagnosis based on published treatment guidelines, then costed a package of treatment that included antiviral medicines (Acyclovir, Famciclovir, or Valacyclovir) and other treatment aids. We imputed public sector pricing of medicines based on unit prices reported in drug price databases [29–32] and prior reviews [33,34]. We calculated total annual costs based on the number of cases expected in each natural history state, the unit costs of treatment, the number of clinic visits expected, and care-seeking proportions, which we calculated for each WHO region based on a review of the literature. We assumed that the cost of providing treatment also included the cost of personnel and facilities, which were reflected in the unit costs of outpatient visits, available from Moses and colleagues [35] (S1 Appendix, section 3.C).

To estimate indirect economic losses, which included wage losses to patients due to absenteeism, we assumed that individuals with symptomatic infections missed 0.4 work days of work for mild and moderate episodes and 0.8 days for severe episodes during a 3-month period. [28] This was then multiplied by the wages in each country, which we predicted by regressing monthly earnings (from ILOSTAT [36]) on the gross national income (GNI) per capita (from the World Bank [37]), and the employed population in each natural history state (based on the employment-to-population ratio available from ILOSTAT [36]) and the proportion of this population who have symptomatic episodes, based on the relevant natural history proportions (Table 1 and S1 Appendix, section 3.D).

To calculate quality-of-life losses, we calculated cases in each disease state surviving through 2019 (based on the survival probability at the median age, available from the United Nations World Population Prospects [38]), then applied the quality-adjusted life year (QALY) weights estimated by Fisman [39], with episode frequency and length determined from our natural history model. We distinguished between individuals being either aware or unaware of their serostatus. In the former case, assumed the asymptomatic utility weight; in the latter case, we assumed the pre-genital herpes utility weight [39]. To estimate the consumption value of the quality-of-life losses, we multiplied the QALYs lost by the value of a statistical life year (VSLY) in each country, which we calculated based on the value of a statistical life (VSL) in the US, using benefits transfer [40] (S1 Appendix, section 3.E).

### Losses due to HIV attributable to HSV-2

We estimated incident HIV cases in 2019 attributable to incident and prevalent HSV-2 infections in 2018 by first calculating the population attributable fractions (PAFs), based on corresponding relative risks (RRs) [17], using the classic (Levin’s) epidemiological formula for polytomous exposures [41]. We applied the PAFs to country estimates of HIV incidence gathered from country projection files from UNAIDS [42] (S1 Appendix, section 4.A). To estimate the direct cost of antiretroviral therapy (ART) and related service delivery and diagnostics, we assumed that patients are diagnosed midyear and are placed on first-line ART immediately thereafter. We multiplied the proportion of the attributable population expected to survive through 2019 by the regional per-patient-per-year unit costs, which we constructed as the sum of service delivery, laboratory diagnostic, and ART costs for 2019 [43] (range: US$315 to US$2,566 for first-line; US$ US$567 to US$3,182 for second-line [43]). We applied ART coverage rates available from UNAIDS AIDSInfo to adjust for utilization [44] (S1 Appendix, section 4.B).

To arrive at wage losses due to absenteeism, we assumed that 90% of the HIV population was of working age [45] and calculated the population expected to be working after adjusting for HIV-related deaths in 2019. We computed pooled values for absent days when on ART and when not on ART for 6 months, based on a rapid literature review and meta-regression. We computed the daily wages as previously described, which we used to calculate the total wages lost due to the absent days on the basis of whether an individual was receiving ART. We assumed that individuals who are on ART have remained on ART for at least 6 months (S1 Appendix, section 4.C).

We report losses annually and during lifetime, with ranges corresponding to the upper and lower bounds of HSV-2 incidence and prevalence. We assume infection at median age and life expectancy at that age. We also report the government versus out-of-pocket (OOP) share of losses [46] (S1 Appendix, section 6). All reported values are in 2019 US$ rates, using exchange rates and deflator values from IMF [47]. We conducted a probabilistic sensitivity analysis to evaluate the sensitivity of the estimates to parameter uncertainty and choice. We sampled 5,000 draws for parameter values using a Latin hypercube sampling algorithm [48], for parameter boundaries described in S1 Appendix and Table 3. All calculations including the life table calculations and sensitivity analysis were performed using Stata (IC version 14.2) and Microsoft Excel for Mac (version 16.45). We provide the Consolidated Health Economic Evaluation Reporting Standards (CHEERS) guidelines in S1 Checklist. A prospective protocol or analysis plan was not constructed prior to undertaking the analysis.

## Results

### Losses due to genital herpes

**Fig 1:**
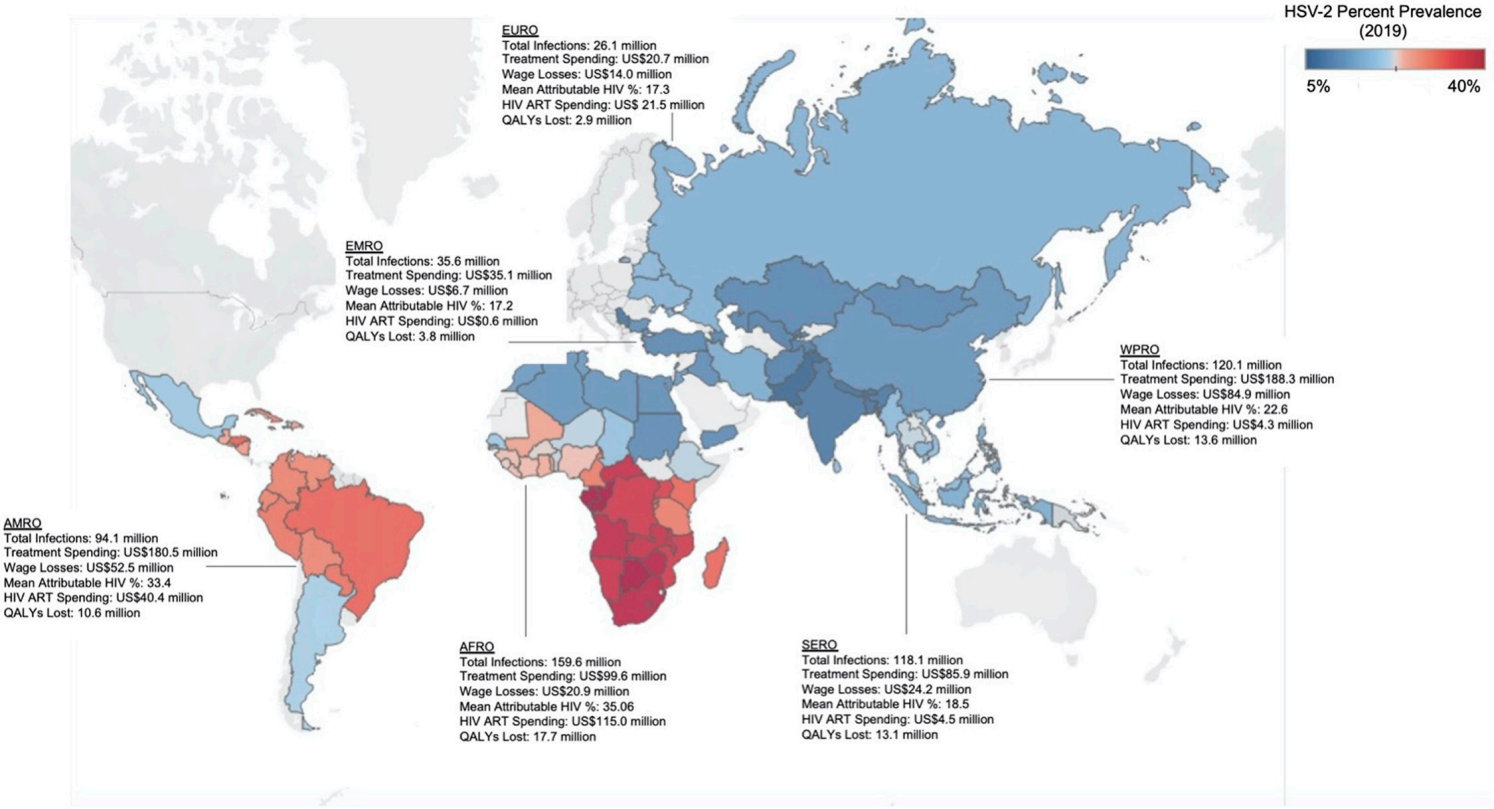
GH cases, spending on GH treatment, wage losses due to GH, HIV cases attributable to HSV-2, related ART spending, and QALY losses due to GH in 90 LMICs, by WHO region (2019). Spending on treatment includes spending on medicines and outpatient care. Estimates assume a single clinic visit per year. Wage losses are due to genital herpes related absenteeism. Estimates assume 1 absent day per year due to a first episode infection in incident cases. The attributable HIV population is represented as a regional mean and is due to incident and prevalent HSV-2 infections where the RR is assumed to be 2.7 for prevalent HSV-2 infections and 4.7 for incident HSV-2 infections^8^. Total ART spending includes service delivery and laboratory diagnostics costs and assumes midyear initiation. The total QALY losses represent losses if the serostatus is known where the asymptomatic QALY weight is assumed to be 0.89^33^. WHO regions include the Africa (AFRO), Americas (AMRO), Southeast Asian (SERO), European (EURO), Eastern Mediterranean (EMRO), and the Western Pacific (WPRO) regions. Base map from Natural Earth, http://www.naturalearthdata.com/about/terms-of-use/ (https://www.naturalearthdata.com/downloads/50m-cultural-vectors/50m-admin-0-countries-2/). ART, antiretroviral therapy; GH, genital herpes; HSV-2, herpes simplex virus type 2; LMIC, low- and middle-income country; QALY, quality-adjusted life year; RR, relative risk; WHO, World Health Organization.

There were 553.60 million individuals with incident and prevalent HSV-2 infections in the 90 LMICs in 2019 [20] (range: 453 to 666 million). Our model-based estimates suggest that 171.7 million of these individuals (31.0%) were in the WHO Africa region (S2 Appendix, section 1). We estimated the costs of medicines, clinical examinations, and diagnostic testing under 2 scenarios: (1) where a clinic visit was made per episode (a visit for the first episode and 5 to 7 visits for recurrences per year); or (2) where 2 clinic visits were made per year irrespective of recurrence frequency [28]. For scenario 1, we calculated the cost of medicines for episodic therapy to be US$737.1 million (range: US$602.5 million to US$887.0 million). For scenario 2, we calculated a cost of medicines to be US$161.0 million (range: US$131.5 million to US$193.3 million). If suppressive therapy is made available to patients with frequent recurrences (under scenario 2), then the costs rise to US$2.2 billion (range: US$1.5 billion to US$2.3 billion) (Table 1 and Fig 1 and S2 Appendix, section 1).

For scenario 1, the cost of clinical examinations and supplies that we assumed was included in outpatient unit costs are US$59.9 billion (range: US$49.0 billion to US$71.0 billion). For scenario 2, the costs are US$8.7 billion (range: US$7.1 billion to US$10.4 billion). If individuals avoided clinic visits and purchased medicines themselves, as suggested by Parker and colleagues [49], then assuming a single clinic visit for the first episode (at regional care-seeking rates), outpatient costs reduce to US$593.8 million (range: US$493.6 million to US$707.7 million). The corresponding medicines cost is US$16.6 million (range: US$13.8 million to US$19.8 million) (Table 2 and Fig 1 and S2 Appendix, section 2).

**Table 2:** Treatment and productivity losses due to genital herpes in 90 LMICs, by WHO region (2019).

If a clinic visit is made per episode for a first episode and 5–7 recurrences per year									
**WHO region**	**Prevalent infections**	**Incident infections**	**Medicines cost**	**Outpatient cost**	**Total treatment cost**	**OOP treatment cost**	**Wage losses– 1.6 absent days (first or recur. episode), 3.2 (freq. recurrence)**	**QALYs lost–if serostatus unknown**	**Consumption value–if serostatus unknown**
AFRO	141 million (117–168)	18.6 million (15.7–21.8)	$212.4 million (176.8–252.1)	$9.3 billion (7.8–11.0)	US$9.51 billion (8.0–11.2)	US$2,67 billion (2.2–3.2)	US$6.1 billion (4.5–6.0)	1.8 million (1.5–2.1)	US$1.87 billion (1.6–2.2)
AMRO	84.2 million (69.9–100)	9.9 million (8.3–11.6)	$126.0 million (104.2–150.0)	$18.5 billion (15.3–22.0)	US$18.63 billion (15.4–22.1)	US$5,86 billion (4.8–7.0)	US$16.4 billion (11.4–15.2)	1.1 million (0.9–1.3)	US$5.14 billion (4.2–6.1)
EMRO	31.4 million (25.1–38.5)	4.2 million (3.4–5.1)	$47.2 million (37.8–57.9)	$3.4 billion (2.7–4.1)	US$3.4 billion (2.7–4.1)	US$1.43 billion (1.2–1.8)	US$2.0 billion (2.0–2.7)	0.4 million (0.3–0.5)	US$911 million (735.0 mil–1.1 bil)
EURO	23.4 million (19–28.4)	2.7 million (2.1–3.2)	$35.0 million (28.4–42.3)	$2.2 billion (1.7–2.6)	US$2.19 billion (1.8–2.6)	US$805 million (654.6–975.8)	US$4.5 billion (4.6–6.1)	0.3 million (0.2–0.4)	US$2.13 billion (1.7–2.6)
SERO	104 million (83.9–126)	14.1 million (11.5–16.9)	$156.0 million (126.0–189.3)	$8.0 billion (6.5–9.7)	US$8.2 billion (6.7–9.9)	US$3.45 billion (2.8–4.2)	US$7.2 billion (6.4–8.7)	1.33 million (1.1–1.6)	US$2.53 billion (2.1–3.1)
WPRO	107 million (86.2–130)	13.1 million (10.8–15.9)	$161.6 million (129.4–195.4)	$18.5 billion (14.9–22.5)	US$18.67 billion (15.0–22.7)	US$6.96 billion (5.6–8.5)	US$25.7 billion (25.0–33.9)	1.38 million (1.1–1.7)	US$9.02 billion (7.2–11.0)
If a clinic visit is made for a first episode and for 1 recurrence, per year							**3.2 absent days per episode**	**If serostatus known**	**If serostatus known**
AFRO	141 million (117–168)	18.6 million (15.7–21.8)	$46.4 million (38.7–55.0)	US$1.4 billion (1.1–1.6)	US$1.4 billion (1.2–1.6)	US$395.5 million (328.7–469.5)	US$9.3 billion (6.9–9.1)	17.7 million (14.7–21.0)	US$18,4 billion (15.3–21.7)
AMRO	84.2 million (69.9–100)	9.9 million (8.3–11.6)	$27.3 million (22.6–32.5)	US$2.6 billion (2.2–3.2)	US$2.7 billion (2.2–3.2)	US$851.6 million (704.1 mil–1.0 bil)	US$24.9 billion (17.3–23.1)	10.6 million (8.8–12.6)	US$50,4 billion (41.7–60.1)
EMRO	31.4 million (25.1–38.5)	4.2 million (3.4–5.1)	$10.3 million (8.3–12.6)	US$487 million (393.4–592.3)	US$497.3 million (401.7–604.9)	US$209.9 million (169.7–256.1)	US$3.1 billion (3.0–4.1)	3.8 million (3.0–4.6)	US$8.94 billion (7.2–10.9)
EURO	23.4 million (19–28.4)	2.7 million (2.1–3.2)	$7.6 million (6.1–9.1)	US$311 million (252.5–376.3)	US$318.6 billion (258.7–385.5)	US$116.9 million (95.3–142.1)	US$6.8 billion (6.9–9.3)	2.9 million (2.4–3.5)	US$20.9 billion (17.1–25.3)
SERO	104 million (83.9–126)	14.1 million (11.5–16.9)	$34.2 million (27.6–41.4)	US$1.2 billion (950.3 mil–1.4 bil)	US$1.2 billion (977.0 mil–1.5 bil)	US$509.6 million (412.4–617.0)	US$10.9 billion (9.7–13.2)	13.1 million (10.6–15.9)	US$24.8 billion (20.1–30.1)
WPRO	107 million (86.2–130)	13.1 million (10.8–15.9)	$34.9 million (28.2–42.5)	US$2.7 billion (2.2–3.3)	US$2.7 billion (2.2–3.3)	US$1.0 billion (817.1 mil–1.2 bil)	US$39.1 billion (38.0–51.5)	13.6 million (10.9–16.5)	US$88.5 billion (71.1–107.7)

Total costs are reported with ranges corresponding to upper and lower bounds of HSV-2 incidence and prevalence. Cost of medicines includes medicines and diagnostics. Cost of outpatient care includes the cost of personnel and facilities. OOP costs are calculated based on percent OOP share of current health expenditure from the WHO Global Health Expenditure database^48^. All treatment-related costs assume regional care-seeking rates calculated based on a literature review of care-seeking (S1 Appendix). Wage losses assume country-level daily wage rates calculated based on historical wage rates from ILOSTAT^30^. Frequent recurrences refer to more than 10 recurrences per year. QALY losses calculated based on utility weights reported by Fisman^33^, assuming 5 recurrences and 7 recurrences. Both calculations assume midyear incidence for recently acquired infections. If serostatus is unknown, assumed that the asymptomatic utility weight is the same as the case where the individual does not have genital herpes. If serostatus is known, then assumed the asymptomatic utility weight reported by Fisman^33^ (for genital herpes patients). Consumption value calculated assuming population in each country to be at median age, experiencing life expectancy at that age. Consumption value calculation uses country VSLYs calculated based on the VSL in the United States using benefits transfer^34^. The WHO regions for which costs are reported include the Africa Region (AFRO), the Region of the Americas (AMRO), the Eastern Mediterranean Region (EMRO), the European Region (EURO), the Southeast Asia Region (SERO), and the Western Pacific Region (WPRO).

HSV-2, herpes simplex virus type 2; LMIC, low- and middle-income country; OOP, out-of-pocket; QALY, quality-adjusted life year; VSL, value of a statistical life; VSLY, value of a statistical life year; WHO, World Health Organization.

Wage losses [50–53], for the 328.3 million employed of the 553.6 million with incident and prevalent HSV-2 infections (59.3%), when considered under the scenarios of: (1) where individuals experienced 1.6 absent days per episode for first and recurrent episodes (217,124,089 of the 285,893,780 symptomatic individuals) and 3.2 absent days per episode for frequent recurrences (68,769,693 of the 285,893,780 symptomatic individuals) [28]; and (2) where individuals with incident infections experienced 1 absent day per year due to a first episode [28]—amount to US$62.0 billion (range: US$53.9 billion to US$72.6 billion) and US$203.4 million (range: US$167.0 million to US$224.7 million), respectively. The daily wages ranged between US$1.56 and US$41.99 (mean = 14.32; SD = 11.73). The absent days we assumed in scenario (1) [28] are consistent with what others have reported [12] (Table 2 and Fig 1 and S2 Appendix, section 3).

We estimated that 61.7 million QALYs would be lost due to genital herpes in 2019 (range: 50.4 million to 74.2 million), provided patients knew of their serostatus even when asymptomatic. If they were unaware of their serostatus, then less than 10% of those QALYs (6.3 million; range: 5.1 million to 7.6 million) would be lost. In the former case, the total consumption value of QALYs lost is US$212.0 billion (range: US$172.6 billion to US$255.7 billion); in the latter case, it is US$21.6 billion (range: US$17.6 billion to US$26.1 billion) (Table 2 and Fig 1 and S2 Appendix 2, section 4).

These figures suggest that in the former case, the mean disutility cost—the average monetary amount that an individual would be willing to pay to avoid the disutility of the disease, is US$343.27 (95% CI: US$272.41 to US$414.14); in the latter case, it is US$34.97 (95% CI: US$27.75 to US$42.19). The value of a QALY in each region, in both cases, ranges between US$1,013.13 (95% CI: US$640.04 to US$1,386.22) in the Africa region and US$5,320.78 (95% CI: US$3,136.80 to US$7,504.76) in the European region. This disutility cost is in addition to the mean cost of treatment per infection, which was US$63.19 (95% CI: US$51.73 to US$74.65). When wage losses are included, this cost increases to US$183.08 (95% CI: US$153.60 to US$212.55). If the unit costs are assumed to remain constant, then the average lifetime cost to the individual, assuming that an individual becomes symptomatic at median age and lives through the expected life expectancy for that age, is US$3,000.97 (95% CI: US$2,462.92 to US$3,539.02) (Table 2 and Fig 1 and S2 Appendix, section 4 and section 8).

### Losses due to HIV attributable to HSV-2

Of the 1,541,644 incident HIV cases in 2019 [20], 522,477 (33.89%) are attributable to the 553.6 million incident and prevalent HSV-2 cases in 2019. We assumed an RR of 4.7 (95% CI: 2.2 to 10.1) for incident HSV-2 infections and an RR of 2.7 (95% CI: 2.2 to 3.4) for prevalent HSV-2 [17]. Of the 522,477 cases, 35,511 were due to incident HSV-2 infections. The 1,541,644 incident HIV cases result in US$561.2 million in ART-related costs (range: US$384.4 million to US$823.0 million), if provided at the 2019 country coverage rates (including service delivery and laboratory diagnostics costs), assuming full year initiation. Of this cost, US$186.4 million (33.2%) (range: US$163.6 million to US$209.5 million) is due to the incident and prevalent HSV-2 cases. The share due to incident HSV-2 infections is modest—US$12.2 million (range: 10.2 million to 14.5 million) (2.2%) (Table 3 and Fig 1 and S2 Appendix, sections 5 and 6).

**Table 3:** Losses due to HIV potentially attributable to HSV-2 in 90 LMICs, by WHO region (2019).

Incident HIV cases due to incident and prevalent HSV-2 cases in 2019								
**WHO region**	**Total HIV population**	**Attributable HIV population**	**Mean attributable percent**	**Total ART cost**	**Total attributable ART cost**	**Mean attributable ART cost percent**	**Wage losses average number of days missed**	**Attributable wage loss percent**
AFRO	1,060,000 (701,709–1.6 mil)	409,000 (363,250–455,305)	35.06 (30.8–39.4)	$298 million (198.9–438.4)	$115.0 million (102.2–128.1)	38.6% (30.8–39.4)	$12.8 million (11.4–14.1)	31.6 (27.7–35.4)
AMRO	113,000 (78,934–163,937)	37,800 (33,007–42,637)	33.45 (29.1–37.8)	$120 million (84.3–174.3)	$40.4 million (35.2–45.5)	33.7% (29.1–37.8)	$3.0 million (2.7–3.4)	30.1 (26.2–34.0)
EMRO	26,947 (4,857–107,317)	3,952 (3,250–4,709)	17.16 (14.2–20.3)	$3.7 million (1.0–12.4)	$645,000 (536,540–760,900)	17.4% (14.2–20.3)	$60,364 (49,246–72,387)	15.5 (12.8–18.3)
EURO	148,000 (114,779–195,639)	33,169 (28,121–38,451)	17.32 (14.4–20.4)	$96.2 million (73.6–127.8)	$21.5 million (18.2–24.9)	22.4% (14.4–20.4)	$4,0 million (3.4–4.7)	15.6 (13.0–18.3)
SERO	104,000 (69,145–159,406)	18,680 (15,694–21,840)	18.47 (15.5–21.6)	$24.1 million (16.2–36.7)	$4.5 million (3.8–5.3)	18.8% (15.5–21.5)	$815,000 (690,198–946,211	16.6 (14.0–19.4)
WPRO	88,804 (47,501–154,175)	19,514 (16,424–22,779)	22.63 (19.1–26.4)	$19.1 million (10.3–33.3)	$4.3 million (3.6–5.0)	22.5% (19.1–26.4)	$1.2 million (1.0–1.4)	20.4 (17.2–23.7)
Incident HIV cases due to incident HSV-2 cases in 2019							**Absenteeism eliminated when on ART**	
AFRO	1,060,000 (701,709–1.6 mil)	409,000 (363,250–455,305)	2.44 (2.0–2.9)	$298 million (198.9–438.4)	$8.1 million (6.8–9.5)	2.7% (2.0–2.9)	$6.36 million (5.7–7.0)	31.6 (27.7–35.4)
AMRO	113,000 (78,934–163,937)	37,800 (33,007–42,637)	2.21 (1.8–2.6)	$120 million (84.3–174.3)	$2.5 million (2.1–3.0)	2.1% (1.8–2.6)	$1.59 million (1.4–1.8)	30.1 (26.2–34.0)
EMRO	26,947 (4,857–107,317)	3,952 (3,250–4,709)	1.03 (0.8–1.3)	$3.7 million (1.0–12.4)	$38,135 (30,903–47,056)	1.0% (0.8–1.3)	$52,090 (42,418–62,554)	15.5 (12.8–18.3)
EURO	148,000 (114,779–195,639)	33,169 (28,121–38,451)	0.99 (0.8–1.2)	$96.2 million (73.6–127.8)	$1.0 million (0.8–1.3)	1.1% (0.8–1.2)	$3.12 million (2.6–3.6)	15.6 (13.0–18.3)
SERO	104,000 (69,145–159,406)	18,680 (15,694–21,840)	1.10 (0.9–1.4)	$24.1 million (16.2–36.7)	$261,000 (212,385–317,117)	1.1% (0.9–1.4)	$436,000 (367,845–507,611)	16.6 (14.0–19.4)
WPRO	88,804 (47,501–154,175)	19,514 (16,424–22,779)	1.55 (1.3–1.9)	$19.1 million (10.3–33.3)	$288,000 (233,816–352,312)	1.5% (1.3–1.9)	$781,000 (655,049–914,255)	20.4 (17.2–23.7)

Attributable HIV cases calculated based on Levin’s classic formula^35^ for each country and then aggregated by WHO region. Pooled adjusted RRs for the general population were used^8^. Mean attributable percent reported with ranges corresponding to upper and lower bounds of HSV-2 incidence and prevalence. ART cost calculation assumes full year provision. The average number of days missed in wage loss calculation based on a rapid review of literature of productivity impacts of HIV (see S1 Appendix). The WHO regions for which costs are reported include the Africa Region (AFRO), the Region of the Americas (AMRO), the Eastern Mediterranean Region (EMRO), the European Region (EURO), the Southeast Asia Region (SERO), and the Western Pacific Region (WPRO).

ART, antiretroviral therapy; HSV-2, herpes simplex virus type 2; LMIC, low- and middle-income country; RR, relative risk; WHO, World Health Organization.

We estimated that 1,387,480 of the 1,541,644 incident HIV cases would be employed. The HIV status in 470,230 individuals is due to incident and prevalent HSV-2 infections. If these individuals missed 5.3 working days per month on average if not on ART, and 2.5 working days per month on average if they had been on ART for 6 months [54], then their cumulative wage losses amount to US$21.9 million (range: US$19.2 million to US$27.4 million). Assuming that due to new ART regimens, absenteeism is completely eliminated once on ART, then the US$21.9 million figure reduces to US$12.3 million (range: US$10.8 million to US$13.9 million). The wage losses due to incident HIV cases stemming from incident HSV-2 cases is US$1.4 million (Table 3 and S2 Appendix, section 7).

The cost of medicines and outpatient care for genital herpes were most sensitive to care-seeking rates (US$667.9 million to US$950.2 million and US$49.3 billion to US$70.9 billion, respectively, for 25% to 75% of symptomatic individuals seeking care) (S1 Appendix, section 7). Wages losses due to the disease were most sensitive to the proportion experiencing recurrences (US$24.4 billion to US$34.4 billion). For HIV-attributable-to-HSV-2, spending on ART was most sensitive to the RR of prevalent HSV-2 infections (US$144.2 million to US$210.1 million). Wage losses in individuals whose HIV status was attributable to HSV-2 was most sensitive to the proportion of that population expected to be working (US$18.1 million to US$25.8 million) (S2 Appendix, section 10).

### Losses in the WHO Africa region from 2020 to 2030

We modeled the transmission of HSV-2 in the 15- to 49-year-old population in the WHO Africa region between 2020 and 2050. We found that the population with prevalent HSV-2 infections will peak in 2036 and then decline through 2050. Percent prevalence in the population will steadily decline from 31.0% in 2020 to 15.1% by 2050. Percent incidence will also decline from 1.3% to 0.3%. We found that 5.6% of cases in 2019 seroconverted within 1 year of 2019, 34.2% seroconverted between 1 to 9 years (of 2019), and 60.2% seroconverted more than 10 years prior to 2019. From 2020 to 2030, we estimated that 289.9 million individuals with a prevalent HSV-2 infection would have symptomatic genital herpes, and 16.5 million individuals with incident HSV-2 would have symptomatic genital herpes (S2 Appendix, section 9).

The incident and prevalent genital herpes infections can give rise to US$32 million (US$24.0 million to US$40.1 million) to US$4.0 billion (US$3.0 billion to US$5.0 billion in medicines costs from 2020 to 2030 (present value in 2020 assuming a 3.0% discount rate)), depending on whether care is sought once per year or per episode. We assumed that the cost of medicines increased by 10% annually. Incident infections account for US$28.5 to US$62.0 million of this cost. Outpatient costs range between US$494.3 milion (US$370.7 million to US$617.9 million) and US$134.8 billion (US$101.0 billion to US$168.4 billion), taking into account the country-level fluctuations in public sector outpatient costs, which were modeled [55]. Incident infections accounts for US$440.2 million to US$1.6 billion. Wage losses are between US$173.9 million (US$130.4 million to US$217.4 million) to US$92.7 billion (US$69.6 billion to US$115.8 billion), depending on whether a single work day is lost per year or if 1.6 days are lost for first and recurrent episodes and 3.2 days for frequent recurrences [28] (incident infections account for US$78.0 million to US$3.7 billion). Additionally, 871 million QALYs (653.1 million to 1.1 billion) are lost if individuals with genital herpes were aware of their serostatus (2.3 million QALYs due to incident infections and 868.5 million due to prevalent infections). If unaware, then 88 million QALYs would be lost (66.2 million to 110.3 million) (402,794 due to incident infections and 87.9 million due to prevalent infections).

We estimated that by 2020, the PAF of HIV due to prevalent HSV-2 will be 32.8% (95% CI: 26.7% to 29.9%) and due to incident infections will be 4.2% (95% CI: 2.6% to 3.4%). The PAF in 2016 due to prevalent (established) infections was estimated to be 33.7% (95% CI: 24.8% to 43.1%) and for incident (recently acquired) infections was estimated to be 34% (13% to 75%) [56]. Both values are consistent with our predictions. Our estimates suggest that the PAF due to prevalent infections will decline to 31.0% by 2030 and incident infections to 3.6%.

Spending on treatment and wage losses during the 10-year period is at a minimum, US$700.2 million (in 2020 present value). The losses due to new infections is US$546.7 million. While this share seems marginal, especially from the perspective of providing justification for a prophylactic vaccine, it does not include the consumption value of 759,087 QALYs, which represents the quality-of-life losses averted. It also does not include the possible contribution of 32.8% of annual HIV-related losses on average.

### Sensitivity analysis

For genital herpes, the cost of medicines and outpatient care were most sensitive to care seeking. When care seeking proportions were allowed to vary between 0.25% and 0.75%, cost of medicines varied between US$667,912,217 and US$950,186,277, and the cost of outpatient care varied between US$49,342,255,803 and US$70,868,016,632. Wage losses were most sensitive to proportions with recurrences, which when allowed to vary between 69.5% and 92.2%, wage losses varied between US$24,427,759,908 and US$34,447,003,617. The consumption value of QALY losses were most sensitive to the income elasticity assumed in the calculation of the country VSLs, which, when allowed to vary between 0.5 and 1.5, resulted in a consumption value between US$144,845,820,413 and US$316,026,610,248. For HIV attributable to HSV-2, spending on ART was most sensitive to the RR of HIV incidence due to prevalent HSV-2 infections. When allowed to vary between 2.2 and 3.4, spending on ART varied between $144,291,971 and $210,075,984. Wage losses associated with incident HIV cases were most sensitive to the proportion of HIV patients who were assumed to be employed. When allowed to vary between 80% to 90%, wages losses varied between US$18,136,054 and US$25,760,540 (S2 Appendix, section 10).

## Discussion

Even under the most conservative assumptions (1 clinic visit per year), economic losses due to genital herpes in LMICs can be as large as US$39.2 billion. The average annual OOP spending per a case of genital herpes in the Africa and Southeast Asia regions are indeed comparable to the HIV OOP spending per case (US$43.87 in Africa and US$98.30 in Southeast Asia in 2015) [57]. However, genital herpes-related losses are highly sensitive to care-seeking as well as the frequency of recurrences. For example, if a clinic visit is made only for a first episode, outpatient costs are US$593.5 million; if an additional visit is made due to recurrences, the costs are US$8.7 billion, as both recent and established infections can lead to recurrences. As individuals become acclimated to the disease, they are more inclined to seek care from private pharmacies rather than public facilities, which can potentially reduce costs [58,59]. Conversely, if care is sought at public facilities, routine tests are often performed to screen for HIV and other STIs, which can potentially elevate costs [60].

Assuming an epidemiological association between HSV-2 and HIV, which, though well established [8], lacks confirmation of direct causality [4,61], incident and prevalent HSV-2 infections can accounts for nearly 33% of incident HIV infections and associated ART costs in 2019. In the Africa region in particular, 38.6% of ART costs in incident HIV cases are due to prevalent HSV-2 cases. Our model-based estimates suggest that incident PAFs will decline more sharply than prevalent PAFs, which means that new HIV cases due to HSV-2 will accrue at a slower rate, if the incidence of HIV were to remain stable or decline. However, secondary infections are not captured in our PAF estimates and it is plausible that these infections will rise as the susceptible pool increases with reducing HIV prevalence [62]. We also do not adjust for interaction effects between HIV and HSV-2; for example, initiation of ART has been found to have a large effect in triggering genital ulcers and viral shedding [63].

The highest losses due to genital herpes were from reduced quality of life. A total of 61.7 million QALYs could have been lost in 2019 if individuals were aware of their serostatus. The mean quality-of-life cost per an individual, which includes the cost of the psychosocial and psychosexual losses [64], is US$343.27 (95% CI: US$272.41 to US$414.14). This cost varies between US$144.03 in the Africa region and US$758.85 in the European region, due to the difference in mean social willingness to pay in each region, represented by the VSL, which is therefore useful as a reference for pricing an intervention such as a vaccine. The average annual cost of treatment and wage losses (US$63.19; 95% CI: US$51.73 to US$74.65) is nearly half this cost, which underscores the extent to which disability-related losses undervalue overall losses. However, often individuals are unaware of their serostatus [65] in which case the quality-of-life cost is US$34.97 (95% CI: US$27.75 to US$42.19), which is nearly half the cost of disability. This is despite disability losses being episodic while quality-of-life losses being persistent.

Our estimates are not directly comparable to prior estimates as we found no prior evidence of HSV-related economic burden estimates from LMICs other than cost effectiveness analyses (S1 Appendix, section 9). We did not find estimates of HSV-related quality-of-life losses either. Owusu-Edusei’s estimates of direct medical cost and estimates of the lifetime cost of neonatal herpes (also in the US) [9], and Szucs and colleagues’ estimates of direct medical costs and productivity costs [12], are to our knowledge, the only available estimates. Our estimates of the PAFs of HIV due to prevalent HSV-2 (32.8%; 95% CI: 26.7% to 29.9%) and due to incident HSV-2 (4.2%; 95% CI: 2.6% to 3.4%) are however consistent with estimates by Looker and colleagues [8] and Silhol and colleagues [66].

Our findings have several important implications. First, the Africa region bears the brunt of the burden due to genital herpes. This is not evident from treatment and productivity costs alone, however, as the major share of the losses stem from reductions in quality-of-life, which are highest in Africa—nearly 1.7 times the losses in the next highest region (Americas). Spending on ART for incident HIV infections due HSV-2 infections is also highest in the Africa region (US$115 million), which is nearly 3-fold the next highest region (Americas—US$40.4 million). The combined ART and wage losses are also highest (US$127.8 million) and nearly 3-fold the next highest region (Americas: US$43.4 million).

Secondly, our model-based estimates suggest that the number of cases of genital herpes will continue to rise in Africa region through 2036 to 2037, even though the incidence will begin to decline after 2025. Economic losses will correspondingly rise but will accrue at a higher rate than the annual rate that we estimated because our estimates did not include all costs and because we did not include second-order effects. For example, treating HIV can worsen the clinical course of genital herpes. Recent evidence suggests that the initiation of ART has a large effect in triggering genital ulcers and viral shedding in individuals who are seropositive for HSV-2 [63]. These individuals will likely need to be placed on both antiviral treatment and antiretroviral treatment concurrently. Acyclovir is known to reduce progression of HIV [67] and decrease the risk of genital ulcers after initiation of ART [63] though whether the effect of ART is tempered in the presence of Acyclovir is unknown.

Our natural history model, being central to our estimates, is a source of several limitations. As the natural history parameters are based on clinic-based studies, they are prone to uncertainty. Though we accounted for uncertainty in our sensitivity analysis, the model still does not account for the severity of episodes. Due to the absence of model-based estimates for all countries, we relied on GBD genital herpes estimates for countries in regions other than Africa. We checked the consistency of GBD estimates with our model-based estimates and collaborated with the GBD team to correct where needed. We computed the number of HIV cases attributable to HSV-2 using Levin’s classical formula rather than transmission dynamics models. Though not ideal, such classically derived estimates have been found to provide reasonable approximation to model-based estimates [62]. The utility weights that we used to estimate the QALY burden are from high-income countries [39]. Their applicability to LMICs is a limitation. The VSL values that we used to estimate the consumption value of QALY losses, contrary to its name, are based on the willingness to accept greater risk rather than the willingness to pay to avert the risk. Despite this discrepancy, willingness to pay and willingness to accept estimates have been found to be reasonably on par for small risk changes [68]. We recognize that we may undervalue losses due limiting our estimates to the population aged 15 to 49 years. We also recognize that the absence of upper and lower bounds for the modeled HSV-2 incidence and prevalence is a limitation, which we have accounted for by assuming a 25% uncertainty.

Our analysis provides compelling evidence that despite the commonly held notion that HSV-2 is an innocuous infection, it can lead to substantial economic losses in LMICs. While the COVID-19 pandemic has understandably diverted attention from other disease control priorities, neglecting the most ubiquitous of all human infections will undoubtedly carry with it long-lasting economic and societal consequences.

## Supporting information

S1 Appendix Methods appendix. (DOCX)

S2 Appendix Data appendix. (DOCX)

S1 Checklist CHEERS checklist. (DOCX)

## Abbreviations

ART: antiretroviral therapy
COVID-19: Coronavirus Disease 2019
GBD: global burden of disease study
GNI: gross national income
HIV: human immunodeficiency virus
HSV-2: herpes simplex virus type 2
LMIC: low- and middle-income country
OOP: out-of-pocket
PAF: population attributable fraction
QALY: quality-adjusted life year
RR: relative risk
SARS-CoV-2: Severe Acute Respiratory Syndrome Coronavirus 2
VSL: value of a statistical life
VSLY: value of a statistical life year
WHO: World Health Organization
