# Supplementary material for: Estimated economic burden of genital herpes and HIV attributable to herpes simplex virus type 2 infections in 90 low- and middle-income countries: A modeling study
Source: PLoS Med. 2022 Dec 15;19(12):e1003938. doi: 10.1371/journal.pmed.1003938 (PMC9754187; doi:10.1371/journal.pmed.1003938)
Supplement: S1 Appendix — (DOCX) [file pmed.1003938.s001.docx]

**Estimated economic burden of genital herpes and HIV attributable to herpes simplex virus type-2 infections in 90 low and middle income countries: A modeling study**

**Supplementary Appendix 1**

Sachin SILVA^1, 2^, Houssein H AYOUB^3^, Christine JOHNSTON^4,5^, Rifat ATUN^1^, Laith J ABU-RADDAD^6,7,8^

1. Harvard TH Chan School of Public Health, Harvard University

Boston, MA Massachusetts, United States of America

1. University of California, San Francisco, Institute for Global Health Sciences,

San Francisco, California, United States of America

1. Mathematics Program, Department of Mathematics, Statistics, and Physics, College of Arts and Sciences, Qatar University

Doha, Qatar

1. Department of Medicine, University of Washington

, Seattle, Washington, United States of America

1. Vaccine and Infectious Diseases Division, Fred Hutchinson Cancer Research Center Eastlake Building, Day Campus,., Seattle, Washington, United States of America
2. Infectious Diseases Epidemiology Group, Weill Cornell Medicine – Qatar

Doha, Qatar

1. World Health Organization Collaborating Centre for Disease Epidemiology Analytics on HIV/AIDS, Sexually Transmitted Infections, and Viral Hepatitis, Weill Cornell Medicine – Qatar

Doha, Qatar

1. Department of Population Health Sciences, Weill Cornell Medicine, Cornell University,

New York, New York, United States of America

***** sas7443@mail.harvard.edu

Table of Contents

[Section 1: Reference Case 3](#_Toc81857615)

[Section 2: Country Selection 3](#_Toc81857616)

[Section 3: Genital Herpes 3](#_Toc81857617)

[A. Disease Burden 3](#_Toc81857618)

[B. Natural History 4](#_Toc81857619)

[C. Treatment Costs 6](#_Toc81857620)

[D. Productivity Costs 8](#_Toc81857621)

[E. Disutility Costs 8](#_Toc81857622)

[Section 4. HIV Attributable to HSV-2 9](#_Toc81857623)

[A. Disease Burden 9](#_Toc81857624)

[B. Treatment Costs 10](#_Toc81857625)

[C. Productivity Costs 10](#_Toc81857626)

[Section 5. WHO AFRICA Region Estimates 11](#_Toc81857627)

[A. Disease Burden 11](#_Toc81857628)

[B. Economic Losses 12](#_Toc81857629)

[Section 6. Currency Conversion 13](#_Toc81857630)

[Section 7. Sensitivity Analysis 13](#_Toc81857631)

[Section 8. Literature Review 15](#_Toc81857632)

[Section 9. Supporting Tables 19](#_Toc81857634)

**Tables**

[Table 1: HSV-2 Parameter values and assumptions 5](#_Toc111481798)

[Table 2: HIV parameter values and assumptions 10](#_Toc111481799)

[Table 3: Parameters considered in sensitivity analysis 13](#_Toc111481800)

[Table 4: Query strings used in searches 18](#_Toc111481801)

[Table 5: Keywords used in literature searches 19](#_Toc111481802)

[Table 6: Treatment Guidelines Assumed 19](#_Toc111481803)

[Table 7: Literature on care-seeking for sexually transmitted infections 23](#_Toc111481804)

[Table 8: Literature on HIV related productivity losses 30](#_Toc111481805)

[Table 9: Literature on economic outcomes of HSV diseases 31](#_Toc111481806)

[Table 10: Literature on non-economic outcomes of HSV diseases 33](#_Toc111481807)

## Section 1: Reference Case

Our estimates take a broader societal perspective and estimate costs accrued to individuals/households and the government. We estimate economic costs rather than the financial costs – meaning the value of the resources as an opportunity cost, rather than the value of the payment. Our estimates are therefore independent of payment structures. Our estimates are based on guidelines rather than on real-world costs, though we adjust for care-seeking. We also estimate full costs rather than incremental costs. As our time horizon, for the 90 countries, we estimate costs accrued in 2019. For the 45 countries in the Africa region, we use 2020 to 2030 as our time horizon

## Section 2: Country Selection

We began with the 195 countries classified according to the World Bank List of Economies (June 2019 version)[1]. We excluded countries classified as high-income (83 countries). For the remaining 112 countries, we constructed a dataset that included the WHO region[2], World Bank lending group, GDP and GNI (annual and per capita), total population, median population age, 5-year survival at median age, and the abridged life tables[3]. Based on the relative availability and reliability of the data, and the relative contribution of each country towards the prevalent infections in 2019, we additionally excluded 22 countries (Table 1). Our estimates are based on a final set of 90 low-income, lower-middle income and upper middle-income countries, which accounted for 85.22 percent of prevalent cases globally and 86.68 percent of incident cases globally, in 2019. Where data was missing, we imputed using the Locally Weighted Scatterplot Smoothing (LOWESS) fitting routine built into Stata (version 14.2), or by identifying a probability distribution function that best described the trend (based on iterative curve fitting) (using SPC for Microsoft Excel for Mac, version 6.0; BPI, USA), then estimating its parameters using an ordinary least-squares minimization routine via Microsoft Excel’s Solver function (version 16.16.23), then predicting based on the fully specified probability distribution.

## Section 3: Genital Herpes

### A. Disease Burden

We modeled the transmission of HSV-2 in 45 low and middle-income African countries (in the WHO Africa region), using a deterministic compartmental mathematical model structured by sex, age, and sexual activity. The model adapted and extended a recent model characterizing the HSV-2 epidemic in the United States from 1950–2050[4]^,^[4]. The model was parameterized for HSV-2 natural history and epidemiology[5]^,^[4] and then calibrated by fitting to HSV-2 seroprevalence data by sex, and an overall seroprevalence decline by two percent[6].

For the countries that we did not model, we relied on the GBD HSV-2 estimates. GBD estimates are based on the DisMod-MR (version 2.1) generic compartmental disease model and Bayesian meta-regression statistical model, using HSV-2 serosurveys for the general population from 1980 onwards, internal systematic reviews from 2010, 2013 and 2015, the reviews by Looker et al., and ongoing input from collaborators, all amounting to roughly 386 seroprevalence sources from 77 countries.

To establish the reliability of the GBD estimates, we compared them with our model-based estimates for the WHO Africa region for 2019. We found that our prevalence estimates were comparable to the GBD estimates despite differences in data input and methodologies.

### B. Natural History

We assumed that incidence and prevalence reflected a laboratory-confirmed seropositive status (employing a valid serological test for HSV-2 antibodies). We also assumed that all incident cases of genital herpes were primary infections where the host lacked HSV antibodies in acute-phase serum prior to infection[7]. We considered infections acquired <1 year as of January 1, 2019 as *recently acquired* infections and infections acquired >1 year as *established infections*. By this definition, all infections incident in 2019 are recently acquired. To determine the proportion of prevalent infections qualifying as recently vs. established, we used our model-based estimates for the Africa region, calculating the precent of infections acquired <1 year (5.62%), 1-9 years (34.16%) and >10 years (60.21%).

For all infections, we assumed three natural history states[8]^,^[9]^,^[10]: (1) a first episode; (2) recurrent episodes; and (3) frequent recurrences. We defined an episode to mean *a symptomatic genital ulcer disease which was separated in time and space from other genital ulcers*. For recurrent episodes, we assumed the median number of recurrences per year following a recently acquired infection. As frequent recurrences, we considered cases who had at least 10 recurrences per year (following either a recently acquired or established infection). We assumed that incident infections must have a first episode in 2019 before having recurrences. For prevalent infections, aside from the recently acquired share, we assumed that the remainder only had recurrences during 2019.

We assumed that 95% of GUD was due to HSV-2 and that 21.0% (12.7-32.8) of recently acquired infection experience a first episode (up to one year after the infection was acquired). We assumed that 83.8% (69.5-92.2) of those who had a first episode experienced recurrences. Of this share, we assumed that 38% had <10 recurrences which we represented by the mean number of recurrences 4.6 (95%CI 3.9-5.5) in recently acquired infection who have had a documented first episode. We assumed that 20% had at least 10 recurrences.

We assumed that a first episode lasted 20.5 days (18.3-22.8) with a mean duration of local pain of 11.8 days. In recurrent infections, if recent (<1 year), then we assumed that an episode lasted 8.5 days (7.5-9.5), if established within 1-9 years, then 7.2 days (1-23) and if established >10 years then 6.5 days (1-24).


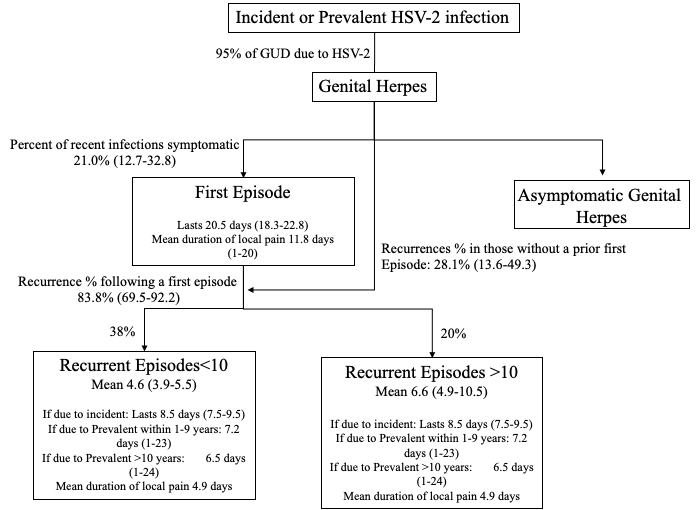


Figure 1: Genital herpes natural history states

Table 1: HSV-2 Parameter values and assumptions

| ***Parameter*** | ***Value*** | ***Source*** |
| --- | --- | --- |
| ***HSV-2***  *Percentage of individuals with recently acquired infection who experience a first episode* | *21.0% (12.7%, 32.8%)* | *Langenberg et al.*[11] |
| *Percentage of individuals with*  *established infection who have one or more GUD recurrences in a year* | *83.8% (69.5%, 92.2%),* | *Beneditti*[12] *and Corey et al.*[13] |
| *Percentage of individuals with*  *established infection who have one or more GUD recurrences in a year* | *33% (24.75%, 41.25%)* | *Beneditti*[12] *and Corey et al.*[13] |
| *Mean number of GUD days per person with recently acquired infection and experiencing a first episode in the absence of antiviral therapy* | *20.5 days (18.3 days, 22.8 days),* | *Beneditti*[12] *and Corey et al.*[13] |
| *Mean annual number of recurrences in those with recently acquired infection* | *4.6 (95%CI 3.9, 5.5)* | *Corey et al.*[14] |
| *Duration of a recurrent episode in a recently acquired infection* | *8.5 days (95%CI 7.5, 9.5)* | *Corey et al.*[14] |
| *Duration of a recurrent episode in an established infection (1- 9 years)* | *7.2 days (95%CI 1, 23)* | *Corey et al.*[14] |
| *Duration of a recurrent episode in an established infection (>10 years) in the absence of antiviral therapy* | *6.5 days (95%CI 1, 24)* | *Corey et al.*[14] |
| *Percent of recently acquired and established infections where recurrences are within the mean* | *74.83% (56.12%, 93.54%)* | *Patel et al.*[14] |
| *Percent of recently acquired and established infections where recurrences exceed the mean (frequent recurrences)* | *25.17% (18.88%, 31.46%)* | *Patel et. al.* |
| *Average number of workdays lost in preceding 3 months due to recurrences within the mean* | *0.4 days (0-21)* | *Patel*[12] |
| *Average number of workdays lost in preceding 3 months due to recurrences exceeding the mean* | *0.8 days (0-21)* | *Patel*[12] |

### C. Treatment Costs

#### Treatment Regimens

For the first clinical episode of GUD, we assumed that the recommendation was for a standard dose of acyclovir over valacyclovir or famciclovir. We assumed the standard dose to be Acyclovir 400 mg orally 3 times a day for 10 days. We additionally considered Acyclovir 200 mg orally 5 times a day for 10 days, Famciclovir 250 mg orally 3 times a day for 10 days and Valacyclovir 500 mg orally twice a day for 10 days (though likely not available in LMICs), as a part of the sensitivity analysis.

For recurrent episodes, we assumed treatment with Acyclovir 400mg orally three times a day for 5 days, 800mg twice a day for 5 days, or 800mg three times a day for 2 days. We assumed that Famciclovir 250 mg orally twice a day for 5 days was also available. As previously, we assumed that Valacyclovir 500 mg orally twice a day for 3days was not available in LMICs, though we estimated its inclusion in the sensitivity analysis. We additionally assumed that for recurrences that were frequent, episodic rather than suppressive therapy was available in LMICs. We did not alter the regimen for individuals living with HIV and people who are immunocompromised

###

#### Costs of Diagnosis

For countries that rely on etiological diagnosis (rather than syndromic algorithms), we assumed that for a patient presenting with genital ulcers, first the exudate from the active lesion was examined using dark ground microscopy, then the scraping from the base of the ulcers were examined to determine the presence of multinucleated giant cells. We assumed that an HSV ELISA and culture tests were performed using swabs taken from the ulcers. We identified the buyer unit cost of HSV ELISAs in countries where they are available from their national medical devices registration databases. We estimated the unit costs for the remaining countries within the same income group using a regression model with GDP per capita ($Y$) as a predictor. We confirmed the validity of the predicted prices by running several regression diagnostics (residuals vs. predicted values, qq-plots).

#### Costs of Treatment

Corbell et al. find that in eight African countries, the country-specific acquisition prices for acyclovir 200 mg was comparable to the median international reference price (IRP) from the Management Sciences for Health's (MSH) 2007 International Drug Price Indicator guide[15]. We used these median prices. Where not available, we imputed using a log-log regression with GDP/capita ($Y$) as a predictor. We confirmed the validity of the predicted prices by running several diagnostics (residuals vs. predicted values, qq-plots). To estimate the share incident on the individual as out-of-pocket (OOP) costs, we used the OOPS % of current health expenditure from the WHO Global Health Expenditure database[16].

#### Care-seeking Proportion

We conducted a literature search to identify studies that evaluated care-seeking behavior of individuals with sexually transmitted infections, and with genital herpes infections specifically (summarized in Table 7). We assumed that unless care is sought within 14 days of an episode start date, care will not be sought at all. We computed a blended care-seeking proportion for men and women reported in each study, and then pooled the mean blended care-seeking proportions on a log scale using the Stata *metan* command (described in section 6).

### D. Productivity Costs

#### Lost Wages

Lost wages due to absenteeism, were calculated by: (1) estimating the working population in each country by multiplying the 15-49 year old population, by the employment/population ratio (both available from ILOSTAT[17]); (2) estimating the proportion of the working population expected to have an incident/prevalent infection; (3) estimating the population in each disease states; (5) multiplying the values in (1), (2) and (3) by the number of absent days expected for each disease state by the daily wage rate for the respective country (available from ILOSTAT as a single wage rate rather than an age-specific rate). Per Patel et al. for mild to moderate episodes, 0.4 work days were lost on average, and 0.8 days were lost on average for a severe episode, over a three-month period[18]. We assumed that frequent recurrences were severe and therefore 0.8 days would be lost per three months. Where the needed ILO wage data was not available, the values were estimated using a regression model with the GNI per capita ($Y$) as a predictor (in constant 2020 US$ rates)[19]. Regression diagnostics (residuals vs. predicted values, qq-plots were etc.) were run to confirm the validity of the regression.

$$Monthly Wages= \beta_{0}+ \beta_{1}Log(Y)+\varepsilon$$

$${\begin{aligned} \\ \beta\end{aligned}}_{0}=11.726;\beta_{1}=0.081$$

### E. Disutility Costs

#### QALY Losses

The population in each disease state surviving from 2019 to 2020 was first calculated by apportioning the incident and prevalent cases into each disease state and then multiplying by each country population’s probability of survival $(_{n}P_{x})$, from the median age $\left( x \right)$ to $n=5$, during the period from 2015-2020 (available as the abridged country-specific life tables for both sexes for up to age 85, from WPP[3]) (We used the${}_{n}P_{x}$ at the upper bound of the interval if the median age exceeded the midpoint of the interval). The total QALY loss due to genital herpes was calculated as the sum of the losses by disease state. For first episodes due to incident cases (recently acquired infections), we assumed midyear incidence and therefore half of year accrual. For first episodes due to established infections, we assumed full year accrual. The loss for each disease state was calculated using the genital herpes-specific QALY weights estimated by Fisman[20] using the time-tradeoff (TTO) method, and applying them to the symptomatic and asymptomatic durations as below.

${QALY}_{First}=S_{First}\frac{\left( QALY_{PreGH}-{QALY}_{Symp} \right)}{365}D_{Sym}+$ $S_{Symp}\frac{\left( QALY_{PreGH}-{QALY}_{Asymp} \right)}{365}{(365-D}_{Symp}$)

${QALY}_{Recur}=S_{Recur}\frac{\left( QALY_{PreGH}-{QALY}_{Recur} \right)}{365}D_{Recur}N_{Recur}+$ $S_{Recur}\frac{\left( QALY_{PreGH}-{QALY}_{Recur} \right)}{365}{[365-(D}_{Symp}N_{Freq})]$

${QALY}_{Freq}=S_{Freq}\frac{\left( QALY_{PreGH}-{QALY}_{Freq} \right)}{365}D_{Freq}N_{Freq}+$ $S_{Freq}\frac{\left( QALY_{PreGH}-{QALY}_{Freq} \right)}{365}{[365-(D}_{Freq}N_{Freq})]$

#### Disutility Costs

To estimate the consumption value of the previously estimated health losses, we multiplied the QALYs (${QALY}_{i}^{j}$) and DALYs (${DALY}_{i}^{j}$) that we estimated, by a country-specific value-of-a statistical-life-year (VSLY) which we calculated by first estimating the value of a statistical life ($VSL_{i}$) in each country based on the value of the VSL in the United States, US$10,951,609 million in 2020 in 2019US$, available from the US Health and Human Services Guidelines[21] at a GNI/capita of US$65, 850 (in 2019 US$) (available from the World Bank), and an income elasticity of 1.0, assuming benefits transfer as recommended by Hammitt and Robinson[22]. The GNI/capita for each country was gathered from the World Bank data tables (in 2019 international $, adjusted for purchasing power parity[23]). The country VSL value represents the societal willingness-to-pay (WTP) for marginal risk reductions. A population‐average VSL of $10 million for example, indicates that the typical individual is willing to pay $1,000 to decrease his or her chance of dying in a given year by 1 in 10,000. Individual WTP can be summed across those affected.

$$VSL_{Target}=VSL_{US}\left( \frac{Y_{Target}}{Y_{US}} \right)^{Elasticity}$$

We then converted the country VSLs $(VSL_{i})$ to values per statistical life year (VSLYs), by dividing by the discounted expected life years remaining for an individual assuming the median age of the population ($LE_{i})$. We used the abridged life tables available from WPP[3]. When using the life table, we used the midpoint of the age interval and in cases where the median age fell in the middle of the age interval, we assumed the upper bound. We discounted assuming a discount rate ($r$) of three percent.

$$Value of health loss={QALY}_{i}^{j}*\frac{VSL_{i}}{\sum\frac{1}{\left( 1+r \right)^{t}}LE_{i}}$$

## Section 4. HIV Attributable to HSV-2

### Disease Burden

We estimated the incident HIV cases in 2020 attributable to incident HSV-2 cases in 2019 (recent infections), and prevalent HSV-2 cases in 2019 (established infections). We first estimated the respective population attributable fractions $(PAF_{P}$ and $PAF_{i})$, using the classical (Levin’s) epidemiological formula for polytomous exposures[24], along with the pooled adjusted relative risks ($RR_{P}$ and $RR_{i}$) estimated by Looker and colleagues for the general population for recent and established infections (2·7; 95% CI: 2·2–3·4 and 4·7; 95% CI: 2·2–10·1 respectively)[25]. We then applied the PAFs to country-level HIV incidence and prevalence estimates from UNAIDS[26]. We did not distinguish by gender or risk group. We assumed that the temporal and lag effects of incident HSV-2 infections giving rise to incident HIV cases is reflected in the relative risks.

$$PAF_{P} =\frac{P_{i}\left( RR_{P}-1 \right)}{P_{i} \left( RR_{P}-1 \right)+1}$$

$$PAF_{I} =\frac{I_{i}\left( RR_{I}-1 \right)}{I_{i} \left( RR_{I}-1 \right)+1}$$

### Treatment Costs

As our focus was on costs associated with incident HIV cases (${Costs}_{i}^{ART})$ in each country ($i$), we used the regional per-patient-per-year unit cost of ART for new patients from Stover and colleagues ($U_{i}^{ART}$), which we multiplied by the incident HIV cases (attributable to incident and prevalent HSV-2), expected to survive through 2019 (incident: $N_{i}^{I}$; Prevalent $N_{i}^{P}$, which we calculated by applying the survival probability at median age in each country (available from WPP[3]) ($S_{i}$), and the ART coverage rates $(A_{i})$, available from UNAIDS[27]. We assumed that HIV incidence was randomly distributed throughout the year in each country and that patients were placed on ART immediately following diagnosis and remained on ART throughout the year. Where ART coverage rates were not available (11 countries), we extracted coverage rates from national reporting. In estimating the unit cost of ART provision in 2019, Stover et al. assumed that the cost of first line drugs would decline by 55% by 2019 across all regions. The costs of service delivery and diagnostics were calculated based on an assumed visit schedule (per WHO guidelines and the recommendations of an expert panel) which reflected one CD4 test at initiation, two viral load tests, four medical consultations, and two drug delivery/adherence support visits in the first year for a patient initiating ART.

$$\sum_{i}^{90} {Costs}_{i}^{ART}=\sum_{i}^{90} N_{i}^{I}{S_{i}U}_{i}^{ART}A_{i}+N_{i}^{P}S_{i}U_{i}^{ART}A_{i}$$

### Productivity Costs

#### Lost Wages

Unlike with genital herpes, reductions in output can arise due to mortality during productive years, reduced labor force participation, absenteeism, presenteeism or premature retirement. To arrive at the share of wage losses due to HIV absenteeism attributable to HSV-2 (${Costs}_{i}^{Wages}$) in each country ($i$), we first conducted a rapid literature review to identify studies that have estimated the number of absent days due to HIV, pre and post ART initiation (see Table 8). We then pooled the values for absent days when on ART ($d_{i}^{No ART}$) and when not on ART for six months ($d_{i}^{ART}$) (see section 6 for a description of pooling). We then computed the daily wages ($W_{i}$) as described in section 3.C.1. We assumed that 90% of the HIV population in LMICs is of productive age[28] and that once on ART, productivity is restored of 75% of pre-symptomatic levels and if not on ART, productivity is reduced by 80% (compared to pre-symptomatic levels). We calculated the wage losses due to absent days on the basis of whether an individual was receiving ART ($A_{i})$, the number of absent days ($d_{i}^{No ART}$, $d_{i}^{ART}$) which we assumed was randomly distributed within the working days for the year (261 for 2019), and the daily wages ($w_{i}$). We assumed that individuals who are on ART, remain on ART through the remainder of the year.

$$\sum_{i}^{90} {Costs}_{i}^{Wages}=\sum_{i}^{90} {w_{i}d}_{i}^{ART}{A_{i}+\left( 1-A_{i} \right)w_{i}d_{i}^{No ART}}$$

Table 2: HIV parameter values and assumptions

| ***Parameter*** | ***Value*** | ***Source*** |
| --- | --- | --- |
| ***HIV*** |  |  |
| *Relative Risk due to incident HSV-2* | *4.7* | *Looker et al.*[25] |
| *Relative Risk due to prevalent HSV-2* | *2.7* | *Looker et al.*[25] |
| *Proportion of HIV population working* | *90%* | *UNAIDS*[29] |
| *Absent days when on ART for six months* | *2.52 days per month* | *Calculated based on literature* |
| *Absent days when not on ART* | *5.31 days per month* | *Calculated based on literature* |
| *Survival with/without ART* | *79.5%*  *(at 12 months)*  *96%*  *( following year)* | *Stover et al.*[30] |
| *Utilization for end-of-life care when not on ART* | *9.7 inpatient days*  *5.5 outpatient* | *Stover et al.*[30] |
| *Utilization of end-of-life care when on ART* | *1/3 of cost without ART* | *Stover et al.*[30] |

## Section 5. WHO AFRICA Region Estimates

### Disease Burden

A deterministic compartmental model was developed, based on extension and adaptation of earlier models[5], [4], to describe HSV-2 transmission in the population. The model consisted of coupled nonlinear differential equations and was structured by gender (females, males), age (20 age groups, 5-year age bands), sexual risk group (5 sexual risk groups), HSV-2 status (uninfected, infected asymptomatic, infected symptomatic), stage of infection (primary, latent, infection reactivation), and time since acquiring the infection (<1 year, 1-9 years, and >10 years).

The five sexual risk groups ranged from lower to higher risk based on number of sexual partners over the last 12 months[31]. The distribution of sexual risk behavior followed a power-law function, as informed by sexual partner data[32]. Age dependence of sexual activity was determined by sexual partner data[31], with sexual debut assumed at age ≥15 years. Sexual mixing by age and risk group was described by mixing matrices that included assortative (i.e. partners choosing partners from within their age or risk group) and proportionate (i.e. no preferential bias in choosing partners based on age or risk group) components, as informed by earlier modeling work[33]^,^ [34].

The model was calibrated through fitting to the gender-stratified HSV-2 seroprevalence data obtained from a recent systematic review and meta-analyses of HSV-2 epidemiology in the WHO Africa Region[35]. The systematic review was informed by the Cochrane Collaboration handbook and reported its findings using the PRISMA guidelines. Pooled mean HSV-2 seroprevalence among women was 52.9% (95% CI: 47.9-57.9%) in the years <2000, 36.2% (95% CI: 31.5-41.1%) in the years 2001-2010, and 43.2% (95% CI: 36.4-50.3%) in the years >2010. Pooled mean HSV-2 seroprevalence among men was 32.5% (95% CI: 27.1-38.1%) in the years <2000, 28.4% (95% CI: 23.2-33.9%) in the years 2001-2010, and 23.7% (95% CI: 16.9-31.3%) in the years >2010. Meta-regressions were conducted to determine the time trend of HSV-2 seroprevalence in the WHO Africa Region.

### Economic Losses

We calculated the regional unit cost of medicine based on the buyer price reported by Sudan’s National Medical Supplies for 2015, adjusting for the remaining countries using as the adjustment factor, the ratio of each country’s GNI/capita to Sudan’s GNI/capita in 2015. We then adjusted for 2019 using historical prices and GNI/capita as a predictor.

For the cost of an outpatient visit, Weaver and colleagues provided us the unit cost of outpatient and inpatient visits from 1995 to 2040 (mean, 2.5, and 97.5 percentiles)[36]. The estimates from 1995 to 2016 are from Moses et al. The forecasts from 2017 to 2040 are from the reference scenario, based on the forecasting methods reported in Foreman et al.[37] The forecasts use the weighted average rate of change between 1995 and 2016, to projects. Weight selection was based on out-of-sample predictive validity, with larger weights assigned to more recent trends. The weighted average of outpatient unit costs were further weighted based on the age-standardized, per capital outpatient utilization rate for both sexes reported by Moses et al.[38]

When calculating wage losses, we first predicted the employment-to-population ratio through 2030, based on values reported by ILOSTAT from 1990 to 2020. For this purpose, we used the Locally Weighted Scatter-plot Smoother (LOWESS) function available from Stata. To calculate the regional daily wage rate for each country and year from 2021 to 2030, we first predicted the GNI/capita through 2040, using GNI/capita from 1950 to 2020 (available from the World Bank). We then calculated the monthly wage rate as described in section 3.D.1 for each country. We then calculated the regional wage rate as a simple mean of the country wage rates.

To model GNI/capita from 2020 to 2030, we employed two approaches. In the first approach, we predicted future growth rates based on historical growth rates from 2000 to 2018 (available from the World Bank) using a non-parametric locally weighted smoothing regression with a bandwidth of 0.8. For this purpose, we used the Locally Weighted Scatter-plot Smoother (LOWESS) function available from Stata. We then applied the predicted growth rates recursively to each predicted value of per capita income. In the second approach, we identified a probability distribution function that best described the per capita income from 1960 to 2018, by iteratively fitting 14 distributions based on Maximum Likelihood Estimation (MLE) (using Matlab version R2017a). To determine the distribution that best described the data, we evaluated the significance level (P<0.05), the Anderson-Darling statistic, the Akaike Information Criterion (AIC), which takes into account the log likelihood[39]) and the likelihood ratio test result. Once we had identified the probability distribution function, to evaluate its parameter values (for example the shape and scale of a Weibull distribution), we used a least-squares minimization routine that we implemented via the Microsoft Excel Optimizer. Using the parameter values and the distribution, we predicted future per capita income through 2050. The second approach was much more resource intensive and did not provide an improvement in fit that was justifiable. We therefore relied on the fitting from the first approach.

## Section 6. Currency Conversion

We report our estimates in 2020 US$ rates. When monetary amounts were reported in international dollars (I$), we applied the purchasing power parity rates (PPP) to first convert to local currency units (LCUs) for the same year, then applied the appropriate deflators to inflate/deflate to 2020 LCUs, then converted the 2020 LCUs to 2020 USDs using the official exchange rates. When amounts were reported in LCUs for a year other than 2020, we first inflated/deflated to 2020 using the appropriate deflators, then converted to 2020 US$ rates by using the corresponding exchange rates. When amounts were reported in USDs for a year other than 2020, we used the deflators to inflate/deflate to 2020 as needed. All deflators, exchange rates, and PPPs were extracted from the World Bank, International Monetary Fund, Penn World Tables, the United Nations National Accounts, and the World Health Organization, and were imputed to provide a complete series for each of the variables between 1950 and 2017.

## Section 7. Sensitivity Analysis

We sampled values for each parameter between their upper and lower bounds, which we either estimated or extracted from literature. We implemented the multiway simulation with 5,000 draws based on Latin Hypercube Sampling and randomization based on Mersenne Twister pseudorandom number generator. We assumed that each parameter was distributed normally, between and upper and lower bounds that we either estimated or extracted from literature (Table 7). We report sensitivity in terms of the range of costs corresponding to each parameter, and the distribution of the means (Supplementary Appendix 2 Section 12).

Table 3: Parameters considered in sensitivity analysis

| ***Parameter*** | ***Sensitivity Analysis*** | ***Source*** |
| --- | --- | --- |
| *Natural History* |  |  |
| *Proportion with genital herpes first episodes* | *Values between 12.7% and 32.8% distributed normally.* | *Langenberg et al.[11]* |
| *Proportion with genital herpes recurrences* | *Values between 69.5% and 92.2% distributed normally.* | *Beneditti[12] and Corey et al.[13]* |
| *Number of clinic visits for recurrences* | *Values between 0 and 12 distributed normally* | *Assumed* |
| *Duration of a genital herpes first episode* | *Values between 18.3 days and 22.8 days distributed normally* | *Beneditti[12] and Corey et al.[13]* |
| *Duration of a genital herpes recurrent episode* | *Values between 7.5 and 9.5 distributed normally* | *Corey et al.[14]* |
| *Care seeking proportion* | *0.25-0.75* | *Assumed based on estimates* |
|  |  |  |
| *Cost of Medicines* |  |  |
| *Cost of medicines for first episodes* | *Values between US$0.93 and US$1.80 distributed normally* | *From MSH*[40] *and modeled costs* |
| *Cost of medicines for recurrences* | *Values between US$0.47 and US$0.90 distributed normally* | *From MSH*[40] *and modeled costs* |
| *Cost of medicines for frequent recurrences* | *Same as for recurrences* | *Assumed* |
|  |  |  |
| *Wage Losses* |  |  |
| *Average number of workdays lost in preceding 3 months due to recurrences within the mean.* | *Values between 0 and 21 distributed normally* | *Patel[12]* |
| *Average number of workdays lost in preceding 3 months due to recurrences exceeding the mean.* | *Values between 0 and 21 distributed normally* | *Patel[12]* |
|  |  |  |
| *Consumption Value of QALY Losses* |  |  |
| *Income elasticity* | *Values between 0.5 to 1.5 distributed normally* | *From Robinson et al[22].* |
|  |  |  |
| *Attributable HIV Cases* |  |  |
| *Relative risk of HIV due to prevalent HSV-2* | *Values between 2.2 and 3.4, distributed normally* | *From Looker et al.*[41] |
| *Relative risk of HIV due to incident HSV-2* | *Values between 2.2 and 10.1 distributed normally.* | *From Looker et al.*[41] |
|  |  |  |
| *Cost of ART* |  |  |
| *Cost of ART provision for a new patient in 2020* | *For first line: Values between US$315-US$2566 distributed normally.*  *For second line: Values between US$567 and US$US$3182* | *From Stover et al.*[42] |
|  |  |  |
| *HIV Wage Losses* |  |  |
| *Proportion of the HIV population who are productive* | *Values between 80 and 90% distributed normally* | *From Resch et al.*[43] |
| *Reduction in productivity when on ART* | *Values between 4% and 25% distributed normally.* | *Based on literature review.* |
| *Reduction in productivity when not on ART* | *Values between 27% and 75% distributed normally.* | *Based on literature review.* |
| *Number of absent days per year when on ART* | *Values between 1 and 5 distributed normally* | *Assumed* |
| *Number of absent days per year when not on ART* | *Values between 2 and 10 distributed normally* | *Assumed* |
|  |  |  |
| *Losses from 2019-2030* |  |  |
| *Annual increase unit cost of medicines* | *Values between 2% and 15%, distributed normally.* | *Assumed* |
| *Discount rate* | *Values between 0% and 6%, distributed normally.* | *Tan-Torres Edejer et al.*[44] |

## Section 8. Literature Review

We first searched for literature on the costs or economics of HSV diseases. We then searched for literature that linked HSV diseases to individual, household, community, and macroeconomic outcomes. We constructed a list of impact areas from the two reviews, then used those areas for targeted searches. Lastly, we searched for literature that identified prior compilations of HSV outcomes or disease reducing interventions. We used the query strings listed in Table 4 along with the impact areas listed in Table 5. We summarize our results in the PRISMA diagram in Figure 2.

We reviewed a total of 2070 articles from the scoping reviews, which suggested that genital herpes is associated with emotional distress and stigma[45]^,^ [46]^,^ [47]^,^ [48]^,^ [49]^,^ [50], intimate partner violence[51]^,^ [52]^,^ [53] marital disruptions[54]^,^ [55]^,^ [56]^,^ [57]^,^ [58] reduced fertility[59]^,^ [60]^,^ [61]^,^ [62]^,^ [63] increased risk of antiviral resistance[64]^,^ [65]^,^ [66]^,^ [67], increased risk of neonatal transmission[68]^,^ [69]^,^ [70] and lost wages[71]^,^ [72]^,^ [73]^,^ [74]. We also found cost of illness estimates to include costs associated with medicines, consultations, clinical examinations, microbiology testing, antibody testing, complete blood counts, urine analysis and hospitalizations[75].


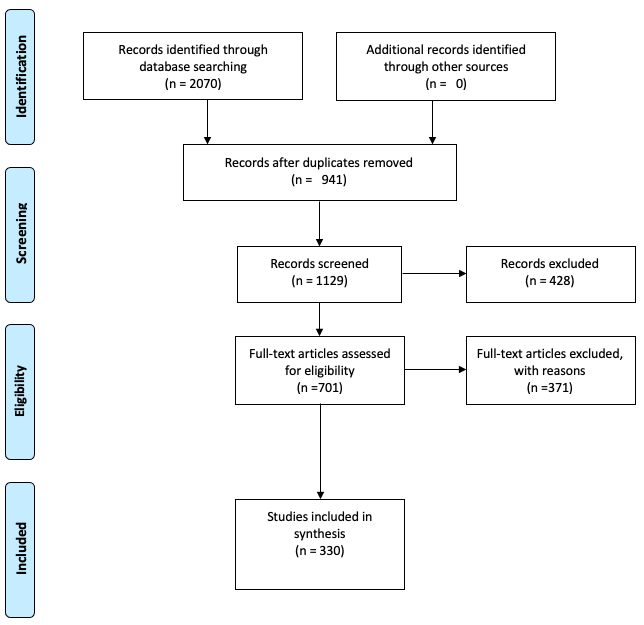


Figure 2: PRISMA diagram for HSV scoping reviews

##

## Section 9. Supporting Tables

Table : Countries excluded and reasons

| ***Country Name*** | ***Reason Excluded*** |
| --- | --- |
| Albania | Data not reliable and prevalent cases in 2019 contribute marginally to total LMIC disease burden |
| American Samoa | Data not reliable and prevalent cases in 2019 contribute marginally to total LMIC disease burden |
| Armenia | Data not reliable and prevalent cases in 2019 contribute marginally to total LMIC disease burden |
| Belize | Data not reliable and prevalent cases in 2019 contribute marginally to total LMIC disease burden |
| Bosnia and Herzegovina | Data not reliable and prevalent cases in 2019 contribute marginally to total LMIC disease burden |
| Cabo Verde | Data not reliable and prevalent cases in 2019 contribute marginally to total LMIC disease burden |
| Comoros | Data not reliable and prevalent cases in 2019 contribute marginally to total LMIC disease burden |
| Costa Rica | Data not reliable and prevalent cases in 2019 contribute marginally to total LMIC disease burden |
| Djibouti | Data not reliable and prevalent cases in 2019 contribute marginally to total LMIC disease burden |
| Dominica | Data not reliable and prevalent cases in 2019 contribute marginally to total LMIC disease burden |
| Equatorial Guinea | Data not reliable and prevalent cases in 2019 contribute marginally to total LMIC disease burden |
| Eritrea | Data not reliable and prevalent cases in 2019 contribute marginally to total LMIC disease burden |
| Eswatini | Data not reliable and prevalent cases in 2019 contribute marginally to total LMIC disease burden |
| Fiji | Data not reliable and prevalent cases in 2019 contribute marginally to total LMIC disease burden |
| Georgia | Data not reliable and prevalent cases in 2019 contribute marginally to total LMIC disease burden |
| Grenada | Data not reliable and prevalent cases in 2019 contribute marginally to total LMIC disease burden |
| Guinea-Bissau | Data not reliable and prevalent cases in 2019 contribute marginally to total LMIC disease burden |
| Guyana | Data not reliable and prevalent cases in 2019 contribute marginally to total LMIC disease burden |
| Kiribati | Data not reliable and prevalent cases in 2019 contribute marginally to total LMIC disease burden |
| Korea, Dem. People's Rep. | Data not reliable and prevalent cases in 2019 contribute marginally to total LMIC disease burden |
| Kosovo | Data not reliable and prevalent cases in 2019 contribute marginally to total LMIC disease burden |
| Kyrgyz Republic | Data not reliable and prevalent cases in 2019 contribute marginally to total LMIC disease burden |
| Lao PDR | Data not reliable and prevalent cases in 2019 contribute marginally to total LMIC disease burden |
| Maldives | Data not reliable and prevalent cases in 2019 contribute marginally to total LMIC disease burden |
| Marshall Islands | Data not reliable and prevalent cases in 2019 contribute marginally to total LMIC disease burden |
| Mauritania | Data not reliable and prevalent cases in 2019 contribute marginally to total LMIC disease burden |
| Micronesia, Fed. Sts. | Data not reliable and prevalent cases in 2019 contribute marginally to total LMIC disease burden |
| Moldova | Data not reliable and prevalent cases in 2019 contribute marginally to total LMIC disease burden |
| Montenegro | Data not reliable and prevalent cases in 2019 contribute marginally to total LMIC disease burden |
| North Macedonia | Data not reliable and prevalent cases in 2019 contribute marginally to total LMIC disease burden |
| Samoa | Data not reliable and prevalent cases in 2019 contribute marginally to total LMIC disease burden |
| Solomon Islands | Data not reliable and prevalent cases in 2019 contribute marginally to total LMIC disease burden |
| Somalia | Data not reliable and prevalent cases in 2019 contribute marginally to total LMIC disease burden |
| South Sudan | Data not reliable and prevalent cases in 2019 contribute marginally to total LMIC disease burden |
| St. Lucia | Data not reliable and prevalent cases in 2019 contribute marginally to total LMIC disease burden |
| Vincent and the Grenadines | Data not reliable and prevalent cases in 2019 contribute marginally to total LMIC disease burden |
| Suriname | Data not reliable and prevalent cases in 2019 contribute marginally to total LMIC disease burden |
| Syrian Arab Republic | Data not reliable and prevalent cases in 2019 contribute marginally to total LMIC disease burden |
| Sao Tome and Principe | Data not reliable and prevalent cases in 2019 contribute marginally to total LMIC disease burden |
| Timor-Leste | Data not reliable and prevalent cases in 2019 contribute marginally to total LMIC disease burden |
| Tonga | Data not reliable and prevalent cases in 2019 contribute marginally to total LMIC disease burden |
| Turkmenistan | Data not reliable and prevalent cases in 2019 contribute marginally to total LMIC disease burden |
| Tuvalu | Data not reliable and prevalent cases in 2019 contribute marginally to total LMIC disease burden |
| Vanuatu | Data not reliable and prevalent cases in 2019 contribute marginally to total LMIC disease burden |
| West Bank and Gaza | Data not reliable and prevalent cases in 2019 contribute marginally to total LMIC disease burden |

Table 4: Query strings used in searches

| ***Review*** | ***Burden Type*** | ***Query String*** |
| --- | --- | --- |
| *Costs of Economics of HSV diseases* | *Economic* | *("Herpes Simplex"[Mesh] OR "Herpes Genitalis"[Mesh] OR "Herpesvirus 1, Human"[Mesh] OR "Herpesvirus 2, Human"[Mesh] OR genital ulcer*[tiab] OR herpes Labialis[tiab] OR genital herpes[tiab] AND ("Costs and Cost Analysis"[Mesh] OR "Economics"[Mesh] OR "economics" [Subheading] OR "Cost of Illness"[Mesh] OR "Cost Savings"[Mesh] OR "Cost-Benefit Analysis"[Mesh] OR "Health Care Costs"[Mesh] OR "Hospital Costs"[Mesh] OR "Drug Costs"[Mesh] OR "Health Expenditures"[Mesh] )* |
| *Accrual of costs and benefits* | *Household* | *("Herpes Simplex"[Mesh] OR "Herpes Genitalis"[Mesh] OR "Herpesvirus 1, Human"[Mesh] OR "Herpesvirus 2, Human"[Mesh] OR genital ulcer*[tiab] OR herpes Labialis[tiab] OR genital herpes[tiab]) AND ("Family Characteristics"[mesh] OR "Family"[mesh] OR household*[tiab])* |
|  | *Community* | *("Herpes Simplex"[Mesh] OR "Herpes Genitalis"[Mesh] OR "Herpesvirus 1, Human"[Mesh] OR "Herpesvirus 2, Human"[Mesh] OR genital ulcer*[tiab] OR herpes Labialis[tiab] OR genital herpes[tiab]) AND ( "Community Health Services/adverse effects"[Mesh] OR "Community Health Services/economics"[Mesh] OR "Community Health Services/education"[Mesh] OR "Community Health Services/epidemiology"[Mesh] OR "Community Health Services/legislation and jurisprudence"[Mesh] OR "Community Health Services/organization and administration"[Mesh] )* |
|  | *Government* | *("Herpes Simplex"[Mesh] OR "Herpes Genitalis"[Mesh] OR "Herpesvirus 1, Human"[Mesh] OR "Herpesvirus 2, Human"[Mesh] OR genital ulcer*[tiab] OR herpes Labialis[tiab] OR genital herpes[tiab]) AND ("Government"[Mesh] OR "State Government"[Mesh] OR "Local Government"[Mesh] OR "Government Programs"[Mesh] OR "Government Agencies"[Mesh] OR "Financing, Government"[Mesh] OR "Government Employees"[Mesh] OR "legislation and jurisprudence" [Subheading])* |
|  | *Macroeconomic* | *("Herpes Simplex"[Mesh] OR "Herpes Genitalis"[Mesh] OR "Herpesvirus 1, Human"[Mesh] OR "Herpesvirus 2, Human"[Mesh] OR genital ulcer*[tiab] OR herpes Labialis[tiab] OR genital herpes[tiab]) AND ("GDP"[tiab] OR “national income” [tiab] OR “foreign investment” [tiab] OR “consumption” [tiab] OR “savings” [tiab] OR “output” [tiab] OR “growth” [tiab])* |

Table 5: Keywords used in literature searches

| ***Category*** | ***Keywords*** |
| --- | --- |
| *Productivity* | *Lifetime productivity, earning$, income, labour, sick days, cognition, cognitive ability, long term disability, mental ability, skills development, school attendance, education, educational outcomes, workforce, GDP* |
| *Household* | *Fertility, pregnancy, child mortality, female workforce, female employment, labour participation, dependency ratio* |
| *Community* | *Herd effects, herd immunity, antibiotic resistance, drug resistant strain, transmission dynamic model, serotype replacement, non-vaccine serotype* |
| *Equity* | *Equity, horizontal equity, vertical equity, distribution$* |
| *Macroeconomy* | *GDP, national income, foreign investment, consumption, saving$, output, growth, sectoral loss* |
|  |  |

Table 6: Treatment Guidelines Assumed

| **Country** | **WHO Region** | **Income Group** | **Guidelines** | **Source** |
| --- | --- | --- | --- | --- |
| Afghanistan | EMRO | Low income | WHO STI guidelines | Assumed |
| Albania | EURO | Upper middle income | European guidelines | Assumed |
| Algeria | AFRO | Upper middle income | WHO STI guidelines | Assumed |
| American Samoa | OTHR | Upper middle income | WHO STI guidelines | Assumed |
| Angola | AFRO | Lower middle income | WHO STI guidelines | Assumed |
| Armenia | EURO | Upper middle income | European guidelines | Assumed |
| Azerbaijan | EURO | Upper middle income | European guidelines | Assumed |
| Bangladesh | SERO | Lower middle income | WHO STI guidelines | Assumed |
| Belarus | EURO | Upper middle income | European guidelines | Assumed |
| Belize | AMRO | Upper middle income | WHO STI guidelines | Assumed |
| Benin | AFRO | Low income | WHO STI guidelines | Assumed |
| Bhutan | SERO | Lower middle income | National guidelines 2009 |  |
| Bolivia | AMRO | Lower middle income | WHO STI guidelines | Assumed |
| Bosnia and Herzegovina | EURO | Upper middle income | European guidelines | Assumed |
| Botswana | AFRO | Upper middle income | Corbell et al.[15] | Corbell et al. |
| Brazil | AMRO | Upper middle income | WHO STI guidelines | Assumed |
| Bulgaria | EURO | Upper middle income | European guidelines | Assumed |
| Burkina Faso | AFRO | Low income | WHO STI guidelines | Assumed |
| Burundi | AFRO | Low income | WHO STI guidelines | Assumed |
| Cabo Verde | AFRO | Lower middle income | WHO STI guidelines | Assumed |
| Cambodia | WPRO | Lower middle income | National Guidelines (2010) |  |
| Cameroon | AFRO | Lower middle income | WHO STI guidelines | Assumed |
| Central African Republic | AFRO | Low income | WHO STI guidelines | Assumed |
| Chad | AFRO | Low income | WHO STI guidelines | Assumed |
| China | WPRO | Upper middle income | WHO STI guidelines | Assumed |
| Colombia | AMRO | Upper middle income | National guidelines 2013 |  |
| Comoros | AFRO | Low income | WHO STI guidelines | Assumed |
| Congo, Dem. Rep. | AFRO | Low income | WHO STI guidelines | Assumed |
| Congo, Rep. | AFRO | Lower middle income | WHO STI guidelines | Assumed |
| Costa Rica | AMRO | Upper middle income | WHO STI guidelines | Assumed |
| Côte d'Ivoire | AFRO | Lower middle income | WHO STI guidelines | Assumed |
| Cuba | AMRO | Upper middle income | WHO STI guidelines | Assumed |
| Djibouti | EMRO | Lower middle income | WHO STI guidelines | Assumed |
| Dominica | AMRO | Upper middle income | WHO STI guidelines | Assumed |
| Dominican Republic | AMRO | Upper middle income | WHO STI guidelines | Assumed |
| Ecuador | AMRO | Upper middle income | WHO STI guidelines | Assumed |
| Egypt | EMRO | Lower middle income | National guidelines 2006 |  |
| El Salvador | AMRO | Lower middle income | WHO STI guidelines | Assumed |
| Equatorial Guinea | AFRO | Upper middle income | WHO STI guidelines | Assumed |
| Eritrea | AFRO | Low income | WHO STI guidelines | Assumed |
| Ethiopia | AFRO | Low income | National guidelines 2015 |  |
| Fiji | WPRO | Upper middle income | Physician input |  |
| Gabon | AFRO | Upper middle income | WHO STI guidelines | Assumed |
| Gambia | AFRO | Low income | WHO STI guidelines | Assumed |
| Georgia | EURO | Lower middle income | European guidelines | Assumed |
| Ghana | AFRO | Lower middle income | WHO STI guidelines | Assumed |
| Grenada | AMRO | Upper middle income | WHO STI guidelines | Assumed |
| Guatemala | AMRO | Upper middle income | WHO STI guidelines | Assumed |
| Guinea | AFRO | Low income | WHO STI guidelines | Assumed |
| Guinea-Bissau | AFRO | Low income | WHO STI guidelines | Assumed |
| Guyana | AMRO | Upper middle income | WHO STI guidelines | Assumed |
| Haiti | AMRO | Low income | WHO STI guidelines | Assumed |
| Honduras | AMRO | Lower middle income | WHO STI guidelines | Assumed |
| India | SERO | Lower middle income | National guidelines 2007 |  |
| Indonesia | SERO | Lower middle income | WHO STI guidelines | Assumed |
| Iran | EMRO | Upper middle income | WHO STI guidelines | Assumed |
| Iraq | EMRO | Upper middle income | WHO STI guidelines | Assumed |
| Jamaica | AMRO | Upper middle income | WHO STI guidelines | Assumed |
| Jordan | EMRO | Upper middle income | WHO STI guidelines | Assumed |
| Kazakhstan | EURO | Upper middle income | European guidelines | Assumed |
| Kenya | AFRO | Lower middle income | Corbell et al. | Corbell et al. |
| Kiribati | WPRO | Lower middle income | WHO STI guidelines | Assumed |
| Kosovo | EURO | Lower middle income | European guidelines | Assumed |
| Lebanon | EMRO | Upper middle income | WHO STI guidelines | Assumed |
| Lesotho | AFRO | Lower middle income | National guidelines 2005 |  |
| Liberia | AFRO | Low income | WHO STI guidelines | Assumed |
| Libya | EMRO | Upper middle income | WHO STI guidelines | Assumed |
| Macedonia | EURO | Upper middle income | European guidelines | Assumed |
| Madagascar | AFRO | Low income | WHO STI guidelines | Assumed |
| Malawi | AFRO | Low income | Corbell et al. and national guidelines (200) | Corbell et al. |
| Malaysia | WPRO | Upper middle income | National guidelines 2008 | Corbell et al. |
| Maldives | SERO | Upper middle income | WHO STI guidelines | Assumed |
| Mali | AFRO | Low income | National guidelines 2007 |  |
| Marshall Islands | WPRO | Upper middle income | WHO STI guidelines | Assumed |
| Mauritania | AFRO | Lower middle income | WHO STI guidelines | Assumed |
| Mauritius | AFRO | Upper middle income | WHO STI guidelines | Assumed |
| Mexico | AMRO | Upper middle income | WHO STI guidelines | Assumed |
| Moldova | EURO | Lower middle income | European guidelines | Assumed |
| Mongolia | WPRO | Lower middle income | WHO STI guidelines | Assumed |
| Montenegro | EURO | Upper middle income | European guidelines | Assumed |
| Morocco | EMRO | Lower middle income | WHO STI guidelines | Assumed |
| Mozambique | AFRO | Low income | WHO STI guidelines | Assumed |
| Myanmar | SERO | Lower middle income | WHO STI guidelines | Assumed |
| Namibia | AFRO | Upper middle income | WHO STI guidelines | Assumed |
| Nauru | WPRO | Upper middle income | WHO STI guidelines | Assumed |
| Nepal | SERO | Low income | WHO STI guidelines | Assumed |
| Nicaragua | AMRO | Lower middle income | WHO STI guidelines | Assumed |
| Niger | AFRO | Low income | WHO STI guidelines | Assumed |
| Nigeria | AFRO | Lower middle income | WHO STI guidelines | Assumed |
| Pakistan | EMRO | Lower middle income | WHO STI guidelines | Assumed |
| Papua New Guinea | WPRO | Lower middle income | WHO STI guidelines | Assumed |
| Paraguay | AMRO | Upper middle income | WHO STI guidelines | Assumed |
| Peru | AMRO | Upper middle income | WHO STI guidelines | Assumed |
| Philippines | WPRO | Lower middle income | WHO STI guidelines | Assumed |
| Romania | EURO | Upper middle income | European guidelines | Assumed |
| Russian Federation | EURO | Upper middle income | European guidelines | Assumed |
| Rwanda | AFRO | Low income | WHO STI guidelines | Assumed |
| Samoa | WPRO | Upper middle income | WHO STI guidelines | Assumed |
| São Tomé and Principe | AFRO | Lower middle income | WHO STI guidelines | Assumed |
| Senegal | AFRO | Low income | WHO STI guidelines | Assumed |
| Serbia | EURO | Upper middle income | European guidelines | Assumed |
| Sierra Leone | AFRO | Low income | WHO STI guidelines | Assumed |
| Solomon Islands | WPRO | Lower middle income | WHO STI guidelines | Assumed |
| Somalia | EMRO | Low income | WHO STI guidelines | Assumed |
| South Africa | AFRO | Upper middle income | Corbell et al. and national guidelines (2015) | Corbell et al. |
| South Sudan | AFRO | Low income | WHO STI guidelines | Assumed |
| Sri Lanka | SERO | Lower middle income | National Guidelines (2000) |  |
| Sudan | EMRO | Lower middle income | WHO STI guidelines | Assumed |
| Suriname | AMRO | Upper middle income | WHO STI guidelines | Assumed |
| Swaziland | AFRO | Lower middle income | WHO STI guidelines | Assumed |
| Syrian Arab Republic | EMRO | Low income | WHO STI guidelines | Assumed |
| Tajikistan | EURO | Low income | European guidelines | Assumed |
| Tanzania | AFRO | Low income | Corbell et al. and national guidelines (2007) | Corbell et al. |
| Thailand | SERO | Upper middle income | Corbell et al. | Corbell et al. |
| Timor-Leste | WPRO | Lower middle income | WHO STI guidelines | Corbell et al. |
| Togo | AFRO | Low income | WHO STI guidelines | Assumed |
| Tonga | WPRO | Upper middle income | WHO STI guidelines | Assumed |
| Tunisia | EMRO | Lower middle income | WHO STI guidelines | Assumed |
| Turkey | EURO | Upper middle income | European guidelines | Assumed |
| Turkmenistan | EURO | Upper middle income | European guidelines | Assumed |
| Tuvalu | WPRO | Upper middle income | WHO STI guidelines | Assumed |
| Uganda | AFRO | Low income | Corbell et al. | Corbell et al. |
| Ukraine | EURO | Lower middle income | European guidelines | Corbell et al. |
| Uzbekistan | EURO | Lower middle income | European guidelines | Assumed |
| Vanuatu | WPRO | Lower middle income | WHO STI guidelines | Assumed |
| Venezuela, RB | AMRO | Upper middle income | WHO STI guidelines | Assumed |
| Vietnam | SERO | Lower middle income | WHO STI guidelines | Assumed |
| Yemen | EMRO | Low income | WHO STI guidelines | Assumed |
| Zambia | AFRO | Lower middle income | Corbell et al. | Corbell et al. |

Table 7: Literature on care-seeking for sexually transmitted infections

| **Author** | **Title** |
| --- | --- |
| Htut KM, Mon MM, Aye ZM, Ni LL. | Young key affected population in Myanmar: are there any challenges in seeking information and care for HIV/sexually transmitted infections and reproductive health? |
| Version 2. F1000Res. 2018 Sep 21;7:1515. doi: 10.12688/f1000research.16029.2. eCollection 2018. |  |
| PMID: 30519458 Free PMC article. |  |
|  |  |
| Leichliter JS, Lewis DA, Sternberg M, Habel MA, Paz-Bailey G. | Health care seeking among men with genital ulcer disease in South Africa: correlates and relationship to human immunodeficiency virus-1 and herpes simplex virus type 2 detection and shedding. |
| Sex Transm Dis. 2011 Sep;38(9):865-70. doi: 10.1097/OLQ.0b013e31821d4ceb. |  |
| PMID: 21844743 Clinical Trial. |  |
|  |  |
| Chuh A, Zawar V, Ooi C, Lee A. | A Case-Control Study in Primary Care Settings on the Roles of Dermatoscopy in Infectious Diseases Affecting the Skin, Part 1: Viral and Bacterial Infections. |
| Skinmed. 2018 Jul 1;16(4):247-254. eCollection 2018. |  |
| PMID: 30207527 |  |
|  |  |
| Fleming DT, Leone P, Esposito D, Heitman CK, Justus S, Chin S, Fife KH. | Herpes virus type 2 infection and genital symptoms in primary care patients. |
| Sex Transm Dis. 2006 Jul;33(7):416-21. doi: 10.1097/01.olq.0000200578.86276.0b. |  |
| PMID: 16601657 Clinical Trial. |  |
|  |  |
| Dorjgochoo T, Noel F, Deschamps MM, Theodore H, Dupont W, Wright PF, Fitzgerald DW, Vermund SH, Pape JW. | Risk factors for HIV infection among Haitian adolescents and young adults seeking counseling and testing in Port-au-Prince. |
| J Acquir Immune Defic Syndr. 2009 Dec 1;52(4):498-508. doi: 10.1097/QAI.0b013e3181ac12a8. |  |
| PMID: 19738486 Free PMC article. |  |
| Goncalez TT, Blatyta PF, Santos FM, Montebello S, Esposti SP, Hangai FN, Salles NA, Mendrone A, Truong HM, Sabino EC, McFarland W. | Does offering human immunodeficiency virus testing at the time of blood donation reduce transfusion transmission risk and increase disclosure counseling? Results of a randomized controlled trial, São Paulo, Brazil. |
| Transfusion. 2015 Jun;55(6):1214-22. doi: 10.1111/trf.13009. Epub 2015 Feb 3. |  |
| PMID: 25646883 Free PMC article. Clinical Trial. |  |
|  |  |
| Namkinga LA, Matee MI, Kivaisi AK, Moshiro C. | Goncalez TT, Blatyta PF, Santos FM, Montebello S, Esposti SP, Hangai FN, Salles NA, Mendrone A, Truong HM, Sabino EC, McFarland W. |
| East Afr Med J. 2005 Mar;82(3):138-43. doi: 10.4314/eamj.v82i3.9270. |  |
| PMID: 16122076 |  |
|  |  |
| Fortenberry JD. | Prevalence and risk factors for vaginal candidiasis among women seeking primary care for genital infections in Dar es Salaam, Tanzania. |
| Herpes. 2004 Apr;11(1):8-11. |  |
| PMID: 15115631 Review. |  |
|  |  |
| Khan AA, Naghma-e-Rehan, Qayyum K, Khan A. | The effects of stigma on genital herpes care-seeking behaviours. |
| J Pak Med Assoc. 2009 Sep;59(9):628-30. |  |
| PMID: 19750861 |  |
|  |  |
| Becker TM, Lee F, Daling JR, Nahmias AJ. | Care seeking for STI symptoms in Pakistan. |
|  | |
| Sex Transm Dis. 1996 Mar-Apr;23(2):138-44. doi: 10.1097/00007435-199603000-00009. |  |
| PMID: 8919741 |  |
|  |  |
| Richards J, Krantz E, Selke S, Wald A. | Seroprevalence of and risk factors for antibodies to herpes simplex viruses, hepatitis B, and hepatitis C among southwestern Hispanic and non-Hispanic white women. |
| Sex Transm Dis. 2008 Dec;35(12):1015-21. doi: 10.1097/OLQ.0b013e318182a596. |  |
| PMID: 18685544 Free PMC article. |  |
| Shevlin E, Morrow RA. | Healthcare seeking and sexual behavior among patients with symptomatic newly acquired genital herpes. |
| J Clin Virol. 2014 Nov;61(3):378-81. doi: 10.1016/j.jcv.2014.08.012. Epub 2014 Aug 27. |  |
| PMID: 25200648 Free PMC article. |  |
|  |  |
| Makasa M, Fylkesnes K, Sandøy IF. | Comparative performance of the Uni-Gold™ HSV-2 Rapid: a point-of-care HSV-2 diagnostic test in unselected sera from a reference laboratory. |
| BMC Public Health. 2012 Jun 6;12:407. doi: 10.1186/1471-2458-12-407. |  |
| PMID: 22672697 Free PMC article. |  |
|  |  |
| Donovan B. | Risk factors, healthcare-seeking and sexual behaviour among patients with genital ulcers in Zambia. |
| Lancet. 2004 Feb 14;363(9408):545-56. doi: 10.1016/S0140-6736(04)15543-8. |  |
| PMID: 14975619 Review. |  |
|  |  |
| Charoenwatanachokchai A, Marin N, Phonrat B, Dhitavat J. | Sexually transmissible infections other than HIV. |
|  | |
| Southeast Asian J Trop Med Public Health. 2017 Mar;48(2):367-75. |  |
| PMID: 29642299 |  |
|  |  |
| Ross SE, Carter B, Lambert S. | Partner Notification Outcomes among Male Gonorrhea Patients at Bangrak Hospital, Bangkok, Thailand. |
| Herpes. 2009 Jan;15(3):46-50. |  |
| PMID: 19306602 |  |
|  |  |
| Wolday D, G-Mariam Z, Mohammed Z, Meles H, Messele T, Seme W, Geyid A, Maayan S. | Seroprevalence of herpes simplex virus-2 infection among women seeking medical care for signs and symptoms of vaginitis. |
| Sex Transm Infect. 2004 Oct;80(5):392-4. doi: 10.1136/sti.2003.005660. |  |
| PMID: 15459409 Free PMC article. |  |
|  |  |
| Berger M, Wagner TH, Baker LC. | Risk factors associated with failure of syndromic treatment of sexually transmitted diseases among women seeking primary care in Addis Ababa. |
|  |  |
| Soc Sci Med. 2005 Oct;61(8):1821-7. doi: 10.1016/j.socscimed.2005.03.025. Epub 2005 Apr 26. |  |
| PMID: 16029778 |  |
|  |  |
| McClelland RS, Baeten JM. | Internet use and stigmatized illness. |
| J Antimicrob Chemother. 2006 Feb;57(2):163-6. doi: 10.1093/jac/dki433. Epub 2005 Dec 6. |  |
| PMID: 16332729 Review. |  |
|  |  |
| Riedner G, Rusizoka M, Hoffmann O, Nichombe F, Lyamuya E, Mmbando D, Maboko L, Hay P, Todd J, Hayes R, Hoelscher M, Grosskurth H. | Reducing HIV-1 transmission through prevention strategies targeting HIV-1-seropositive individuals. |
| Sex Transm Infect. 2003 Oct;79(5):382-7. doi: 10.1136/sti.79.5.382. |  |
| PMID: 14573833 Free PMC article. |  |
|  |  |
| Morris-Cunnington M, Brown D, Pimenta J, Robinson NJ, Miller E. | Baseline survey of sexually transmitted infections in a cohort of female bar workers in Mbeya Region, Tanzania. |
| Sex Transm Dis. 2004 Apr;31(4):243-6. doi: 10.1097/01.olq.0000118081.54177.95. |  |
| PMID: 15028940 Clinical Trial. |  |
|  |  |
| Haseen F, Chawdhury FA, Hossain ME, Huq M, Bhuiyan MU, Imam H, Rahman DM, Gazi R, Khan SI, Kelly R, Ahmed J, Rahman M. | New estimates of herpes simplex virus type 2 seroprevalence in England: 'high' but stable seroprevalence over the last decade. |
| Int J STD AIDS. 2012 Aug;23(8):553-9. doi: 10.1258/ijsa.2012.011373. |  |
| PMID: 22930291 |  |
|  |  |
| Curtis P, Okin M, McGaghie WC, Resnick J, Joseph M, Davis G. | Sexually transmitted infections and sexual behaviour among youth clients of hotel-based female sex workers in Dhaka, Bangladesh. |
| Fam Med. 1990 Nov-Dec;22(6):458-61. |  |
| PMID: 2262108 |  |
|  |  |
| Szucs TD, Berger K, Fisman DN, Harbarth S. | A pilot study of the prevalence of herpes genitalis among selected groups of patients seeking care in a family practice center. |
| BMC Infect Dis. 2001;1:5. doi: 10.1186/1471-2334-1-5. Epub 2001 Jun 28. |  |
| PMID: 11472635 Free PMC article. |  |
|  |  |
| Lewis LM, Rosenthal SL, Succop PA, Stanberry LR, Bernstein DI. | The estimated economic burden of genital herpes in the United States. An analysis using two costing approaches. |
| Int J STD AIDS. 1999 Nov;10(11):703-8. doi: 10.1258/0956462991913376. |  |
|  |  |
| Wellington M, Ndowa F, Mbengeranwa L. | College students' knowledge and perceptions of genital herpes. |
| Sex Transm Dis. 1997 Oct;24(9):528-32. doi: 10.1097/00007435-199710000-00006. |  |
| PMID: 9339971 |  |
|  |  |
| Action Contre SIDA. 1995 Jul;(25):4-5. | Risk factors for sexually transmitted disease in Harare: a case-control study. |
| PMID: 12291920 French. |  |
|  |  |
| Gomes CM, Giraldo PC, Gomes Fde A, Amaral R, Passos MR, Gonçalves AK. | [Management of STDS at the primary care level. Recommendations for STD treatment]. |
| Braz J Infect Dis. 2007 Apr;11(2):254-60. doi: 10.1590/s1413-86702007000200018. |  |
| PMID: 17625773 |  |
|  |  |
| Oliver L, Wald A, Kim M, Zeh J, Selke S, Ashley R, Corey L. | Genital ulcers in women: clinical, microbiologic and histopathologic characteristics. |
| Arch Fam Med. 1995 Mar;4(3):228-32. doi: 10.1001/archfami.4.3.228. |  |
| PMID: 7881604 |  |
|  |  |
| Kwena Z, Sharma A, Wamae N, Muga C, Bukusi E. | Seroprevalence of herpes simplex virus infections in a family medicine clinic. |
| Sex Transm Dis. 2008 May;35(5):480-3. doi: 10.1097/OLQ.0b013e3181644b84. |  |
| PMID: 18360315 |  |
|  |  |
| Fleming DT, Levine WC, Trees DL, Tambe P, Toomey K, St Louis ME. | Provider characteristics among staff providing care to sexually transmitted infection self-medicating patients in retail pharmacies in Kibera slum, Nairobi, Kenya. |
|  | |
| Sex Transm Dis. 2000 Feb;27(2):68-73. doi: 10.1097/00007435-200002000-00003. | Syphilis in Atlanta during an era of declining incidence. |
| PMID: 10676972 |  |
|  |  |
| Go VF, Quan VM, A C, Zenilman JM, Moulton LH, Celentano DD. | Barriers to reproductive tract infection (RTI) care among Vietnamese women: implications for RTI control programs. |
| Sex Transm Dis. 2002 Apr;29(4):201-6. doi: 10.1097/00007435-200204000-00003. |  |
| PMID: 11912460 |  |
|  |  |
| Wakgari N, Woyo T, Kebede E, Gemeda H, Gebremedhin S. | Sexually transmitted disease among street dwellers in southern Ethiopia: a mixed methods study design. |
| BMC Public Health. 2020 Apr 3;20(1):434. doi: 10.1186/s12889-020-08584-x. |  |
| PMID: 32245375 Free PMC article. |  |
|  |  |
| Msuya SE, Mbizvo EM, Stray-Pedersen B, Uriyo J, Sam NE, Rusakaniko S, Hussain A. | Decline in HIV prevalence among women of childbearing age in Moshi urban, Tanzania. |
| Int J STD AIDS. 2007 Oct;18(10):680-7. doi: 10.1258/095646207782193858. |  |
| PMID: 17945046 |  |
|  |  |
| Kwena ZA, Sharma A, Muga C, Wamae N, Bukusi EA. | Management of simulated patients with sexually transmitted infections by staff of retail pharmacies in Kibera slums of Nairobi. |
| East Afr Med J. 2008 Sep;85(9):419-24. doi: 10.4314/eamj.v85i9.117082. |  |
| PMID: 19537413 |  |
|  |  |
| Laga M. | Epidemiology and control of sexually transmitted diseases in developing countries. |
| Sex Transm Dis. 1994 Mar-Apr;21(2 Suppl):S45-50.  PMID: 8042115 Review. |  |
| Mayhew S, Nzambi K, Pépin J, Adjei S. | Pharmacists' role in managing sexually transmitted infections: policy issues and options for Ghana. |
| Health Policy Plan. 2001 Jun;16(2):152-60. doi: 10.1093/heapol/16.2.152. |  |
| PMID: 11358916 |  |
| Bensen C, Stern J, Skinner E, Beutner K, Conant M, Tyring S, Reitano M, Davis G, Wald A. | An interactive, computer-based program to educate patients about genital herpes. |
| Sex Transm Dis. 1999 Jul;26(6):364-8. doi: 10.1097/00007435-199907000-00010. |  |
| PMID: 10417026 |  |
|  |  |
| Parker KA, Koumans EH, Hawkins RV, Massanga M, Somse P, Barker K, Moran J. | Providing low-cost sexually transmitted diseases services in two semi-urban health centers in Central African Republic (CAR): characteristics of patients and patterns of health care-seeking behavior. |
| Sex Transm Dis. 1999 Oct;26(9):508-16. doi: 10.1097/00007435-199910000-00005. |  |
| PMID: 10534204 |  |
|  |  |
| Wald A, Stern JO, Skinner EP, Beutner K, Conant MA, Tyring SK, Reitano MV, Davis G. | Can a multimedia educational computer-based program on genital herpes teach patients about their disease? |
| Prim Care Update Ob Gyns. 1998 Jul 1;5(4):151. doi: 10.1016/s1068-607x(98)00033-x. |  |
| PMID: 10838278 |  |
|  |  |
| Steen R, Soliman C, Mujyambwani A, Twagirakristu JB, Bucyana S, Grundmann C, Ngabonziza M, Moran J. | Notes from the field: practical issues in upgrading STD services based on experience from primary healthcare facilities in two Rwandan towns. |
| Sex Transm Infect. 1998 Jun;74 Suppl 1:S159-65. |  |
| PMID: 10023368 |  |
|  |  |
| Myers JL. | Why do young women get tested for sexually transmitted infections? Evidence from the National Longitudinal Study of Adolescent Health. |
| J Womens Health (Larchmt). 2011 Aug;20(8):1225-31. doi: 10.1089/jwh.2010.2544. Epub 2011 Jun 20. |  |
| PMID: 21689024 |  |
|  |  |
| Grosskurth H, Mwijarubi E, Todd J, Rwakatare M, Orroth K, Mayaud P, Cleophas B, Buvé A, Mkanje R, Ndeki L, Gavyole A, Hayes R, Mabey D. | Operational performance of an STD control programme in Mwanza Region, Tanzania. |
| Sex Transm Infect. 2000 Dec;76(6):426-36. doi: 10.1136/sti.76.6.426. |  |
| PMID: 11221123 Free PMC article. Clinical Trial. |  |
| Brackbill RM, Sternberg MR, Fishbein M. | Where do people go for treatment of sexually transmitted diseases? |
| Fam Plann Perspect. 1999 Jan-Feb;31(1):10-5. |  |
| PMID: 10029927 |  |
|  |  |
| Wright J, Albright TS, Gehrich AP, Dunlow SG, Lettieri CF, Buller JL. | Sexually transmitted diseases in Operation Iraqi Freedom/Operation Enduring Freedom. |
| Mil Med. 2006 Oct;171(10):1024-6. doi: 10.7205/milmed.171.10.1024. |  |
| PMID: 17076459 |  |
|  |  |
| Adler MW. | Sexually transmitted diseases control in developing countries. |
| Genitourin Med. 1996 Apr;72(2):83-8. doi: 10.1136/sti.72.2.83. |  |
| PMID: 8698372 Free PMC article. |  |

Table 8: Literature on HIV related productivity losses

| **Source** | **Parameter Value** |
| --- | --- |
| 1.Habyarimana, J., Mbakile, B. & Pop-Eleches, C. The Impact of HIV/AIDS and ARV Treatment on Worker Absenteeism: Implications for African Firms. The Journal of Human Resources 45, 809–839 (2010). | Year before initiation: absenteeism of enrolled workers increases sharply to a peak of five days/month in last month. First six months after initiation: absenteeism declines sharply. 7 months-4 years after initiation: no difference between enrolled workers and rest of workforce. |
| Iro, O., Amadi, A., & Abanobi, O. (2008, August). The EffectofHIV/AIDS-relatedMorbidityonWorkPro- ductivity: A Case-control Study of Patients on ART in Abia State of Nigeria. Paper presented at the AIDS 2008 􏰑 XVII International AIDS Conference. Mexico City, Mexico. | Mean workdays lost (pB0.05) HIV+ on ART: M=1.06 (SD =1.516). HIV+ not on ART: M=4.16 (SD =3.282). HIV- or unknown serostatus: M=0.05 (SD =0.366). |
| 1.Muirhead, D. et al. Health care costs, savings and productivity benefits resulting from a large employer sponsored ART program in South Africa. undefined /paper/Health-care-costs%2C-savings-and-productivity-from-a-Muirhead-Kumaranayake/1169a2d82967c92cfe28892a31530e7ed10b2929 (2006). | Mean worker absence over 18 months on ART Immediately prior to ART initiation: 7.5 days/month. Six months on ART: 2.9 days/month. 12 months: 2.2 days/month. 18 months: 2.1 days/month. |
| Rosen, S., Ketlhapile, M., Sanne, I., & DeSilva, M.B. (2008). Differences in normal activities, job perfor- mance and symptom prevalence between patients not yet on antiretroviral therapy and patients initiating therapy in South Africa. AIDS, 22(Suppl. 1), S131 S139. | Differences in absenteeism prior to ART up to six months Mean days absent due to health in previous month: Pre-ART 3.1 (SD =7.5); ART 1-6 months 2.7 (SD =7.7) (p =0.0108); ART 1-3 months 4.2 (SD =9.8); ART 3-6 months 1.3 (SD =5.0) (p =0.0353). |
| Beard J, Feeley F, Rosen S (2009) Economic and quality of life outcomes of antiretroviral therapy for HIV/AIDS in developing countries: a systematic literature review. AIDS Care 21: 1343–1356. | Absence pre-ART 3.1 days/month; post-ART 1.3/month at 3-6 months |
| World Health Organization HIV/AIDS Programme (2009) Rapid advice: Antiretroviral therapy for HIV infection in adults and adolescents. Geneva. | Absence pre-ART 78 days/year; post-ART 52 days/year at 28 months |
| Fox MP, McCoy K, Larson BA, Rosen S, Bii M, et al. (2010) Improvements in physical wellbeing over the first two years on antiretroviral therapy in western Kenya | Absence pre-ART 7.5 days/month; post-ART 2.9 days at 6 months, 2.1 days at 18 months |
| King JT Jr, Justice AC, Roberts MS, Chang CC, Fusco JS (2003) Long-term HIV/AIDS survival estimation in the highly active antiretroviral therapy era. Med Decis Making 23: 9–20. | Pre-ART: Sick leave 0.16 months/year (0.14 in controls). Pre-ART 12% ‘fully functioning’; post-ART 84% at 12 months and 91% at 24 months |

Table 9: Literature on economic outcomes of HSV diseases

| ***Year*** | ***Author*** | ***Title*** |
| --- | --- | --- |
| 2006 | Terris-Prestholt, Fern; Vyas, Seema; Kumaranayake, Lilani; Mayaud, Philippe; Watts, Charlotte | The Costs of Treating Curable Sexually Transmitted Infections in Low- and Middle-Income Countries: A Systematic Review: |
| 2008 | Caviness, A. Chantal; Demmler, Gail J.; Swint, J. Michael; Cantor, Scott B. | Cost-effectiveness analysis of herpes simplex virus testing and treatment strategies in febrile neonates |
| 2005 | Little, Sarah E.; Caughey, Aaron B. | Acyclovir prophylaxis for pregnant women with a known history of herpes simplex virus: a cost-effectiveness analysis |
| 2005 | Thung, Stephen F.; Grobman, William A. | The cost-effectiveness of routine antenatal screening for maternal herpes simplex virus-1 and -2 antibodies |
| 2004 | Baker, David; Brown, Zane; Hollier, Lisa M.; Wendel, George D.; Hulme, Lisa; Griffiths, Dorothea A.; Mauskopf, Josephine | Cost-effectiveness of herpes simplex virus type 2 serologic testing and antiviral therapy in pregnancy |
| 2003 | Lairson, David R.; Begley, Charles E.; Reynolds, Thomas F.; Wilhelmus, Kirk R. | Prevention of herpes simplex virus eye disease: a cost-effectiveness analysis |
| 2001 | Qutub, M.; Klapper, P.; Vallely, P.; Cleator, G. | Genital herpes in pregnancy: is screening cost-effective? |
| 1998 | Scott, L. L.; Alexander, J. | Cost-effectiveness of acyclovir suppression to prevent recurrent genital herpes in term pregnancy |
| 1997 | Mennemeyer, S. T.; Cyr, L. P.; Whitley, R. J. | Antiviral therapy for neonatal herpes simplex virus: a cost-effectiveness analysis |
| 1996 | Randolph, A. G.; Hartshorn, R. M.; Washington, A. E. | Acyclovir prophylaxis in late pregnancy to prevent neonatal herpes: a cost-effectiveness analysis |
| 1989 | Binkin, N. J.; Koplan, J. P. | The high cost and low efficacy of weekly viral cultures for pregnant women with recurrent genital herpes: a reappraisal |
| 1984 | Daling, J. R.; Wolf, M. E. | The role of decision and cost analyses in the treatment of pregnant women with recurrent genital herpes |
| 2013 | Owusu-Edusei Jr, Kwame; Chesson, Harrell W.; Gift, Thomas L.; Tao, Guoyu; Mahajan, Reena; Ocfemia, Marie Cheryl Bañez; Kent, Charlotte K. | The estimated direct medical cost of selected sexually transmitted infections in the United States, 2008 |
| 2002 | Ashley, Rhoda L. | Performance and use of HSV type-specific serology test kits. |
| 2015 | Jewell, Britta L.; Cremin, Ide; Pickles, Michael; Celum, Connie; Baeten, Jared M.; Delany-Moretlwe, Sinead; Hallett, Timothy B | Estimating the cost-effectiveness of pre-exposure prophylaxis to reduce HIV-1 and HSV-2 incidence in HIV-serodiscordant couples in South Africa |
| 2009 | Fife, Kenneth H.; Van Der Pol, Barbara; Roth, Alexis M.; Brand, Juanita; Arno, Janet N.; Madlem, Jyl; Juliar, Beth E.; Katz, Barry P.; Williams, James A.; Zimet, Gregory D. | Implementation of routine access to herpes simplex virus type 2 antibody testing in a public health sexually transmitted disease clinic |
| 2011 | Tuite, Ashleigh R.; McCabe, Caitlin J.; Ku, Jennifer; Fisman, David N. | Projected cost-savings with herpes simplex virus screening in pregnancy: towards a new screening paradigm |
|  | | |
| 1987 | Darougar, S.; Woodland, R. M.; Walpita, P. | Value and cost effectiveness of double culture tests for diagnosis of ocular viral and chlamydial infections. |
| 2008 | Vickerman, Peter; Ndowa, Francis; Mayaud, Philippe | Modelling the cost per ulcer treated of incorporating episodic treatment for HSV-2 into the syndromic algorithm for genital ulcer disease |
| 2014 | Garnett, Geoff P. | The theoretical impact and cost-effectiveness of vaccines that protect against sexually transmitted infections and disease |
| 2011 | Vickerman, Peter; Devine, Angela; Foss, Anna M.; Delany-Moretlwe, Sinead; Mayaud, Philippe; Meyer-Rath, Gesine | The cost-effectiveness of herpes simplex virus-2 suppressive therapy with daily aciclovir for delaying HIV disease progression among HIV-1-infected women in South Africa |
| 2016 | Cambiano, Valentina; Miners, Alec; Phillips, Andrew | What do we know about the cost–effectiveness of HIV preexposure prophylaxis, and is it affordable? |
| 2012 | Foss, A. M.; Terris-Prestholt, F.; Cox, A. P.; Heise, L.; Meyer-Rath, G.; Delany-Moretlwe, S.; Mertenskoetter, T.; Rees, H.; Vickerman, P.; Watts, C. H. | Model projections of the population-level impact, on HIV and herpes simplex virus type-2 (HSV-2), and cost-effectiveness of tenofovir gel, an antiretroviral microbicide |
| 2002 | Barnabas, R. V.; Carabin, H.; Garnett, G. P. | The potential role of suppressive therapy for sex partners in the prevention of neonatal herpes: a health economic analysis |
| 2000 | Lafferty, W. E.; Downey, L.; Celum, C.; Wald, A. | Herpes simplex virus type 1 as a cause of genital herpes: impact on surveillance and prevention |
| 2005 | Baker, David A. | Antiviral therapy for genital herpes infections in pregnancy |
| 2001 | Qutub, M.; Klapper, P.; Vallely, P.; Cleator, G. | Genital herpes in pregnancy: is screening cost-effective? |
| 1989 | Binkin, N. J.; Koplan, J. P. | The high cost and low efficacy of weekly viral cultures for pregnant women with recurrent genital herpes: a reappraisal |
| 2015 | Owusu-Edusei, Kwame; Flagg, Elaine W.; Gift, Thomas L. | Hospitalization cost per case of neonatal herpes simplex virus infection from claims data |
| 1989 | Binkin, Nancy J.; Koplan, Jeffrey P. | The High Cost and Low Efficacy of Weekly Viral Cultures for Pregnant Women with Recurrent Genital Herpes: A Reappraisal |
| 2000 | Grobman, W. A.; Peaceman, A. M.; Socol, M. L. | Cost-effectiveness of elective cesarean delivery after one prior low transverse cesarean |
| 1989 | Binkin, N. J.; Koplan, J. P. | The high cost and low efficacy of weekly viral cultures for pregnant women with recurrent genital herpes: a reappraisal |
| 2017 | Korenromp, Eline L.; Wi, Teodora; Resch, Stephen; Stover, John; Broutet, Nathalie | Costing of National STI Program Implementation for the Global STI Control Strategy for the Health Sector, 2016-2021 |

Table 10: Literature on non-economic outcomes of HSV diseases

| ***Year*** | ***Author*** | ***Title*** | ***Impact*** |
| --- | --- | --- | --- |
| 2014 | Abdool Karim, Quarraisha; Kharsany, Ayesha B. M.; Leask, Kerry; Ntombela, Fanelisibonge; Humphries, Hilton; Frohlich, Janet A.; Samsunder, Natasha; Grobler, Anneke; Dellar, Rachael; Abdool Karim, Salim S. | Prevalence of HIV, HSV-2 and pregnancy among high school students in rural KwaZulu-Natal, South Africa: a bio-behavioural cross-sectional survey | Schooling |
| 2007 | Abdulmedzhidova, A. G.; Kurilo, L. F.; Shileĭko, L. V.; Makarova, N. P.; Klimova, R. R.; Kushch, A. A. | [Asymptomatic genital herpes infection and infertility in males] | Infertility |
| 2013 | Andrei, Graciela; Snoeck, Robert | Herpes simplex virus drug-resistance: new mutations and insights | AMR |
| 2012 | Baird, Sarah J.; Garfein, Richard S.; McIntosh, Craig T.; Ozler, Berk | Effect of a cash transfer programme for schooling on prevalence of HIV and herpes simplex type 2 in Malawi: a cluster randomised trial | Schooling |
| 2007 | Bickford, J.; Barton, S. E.; Mandalia, S. | Chronic genital herpes and disclosure.... The influence of stigma | Stigma |
| 2009 | Birdthistle, Isolde; Floyd, Sian; Nyagadza, Auxillia; Mudziwapasi, Netsai; Gregson, Simon; Glynn, Judith R. | Is education the link between orphanhood and HIV/HSV-2 risk among female adolescents in urban Zimbabwe? | Schooling |
| 2007 | Bocharova, E. N.; Zavalishina, L. E.; Bragina, E. E.; Klimova, R. R.; Gusak, Yu K.; Kurilo, L. F.; Shileiko, L. V.; Petrov, A. N.; Frank, G. A.; Kushch, A. A. | Detection of herpes simplex virus genomic DNA in spermatozoa of patients with fertility disorders by in situ hybridization | Infertility |
| 2004 | Breitkopf, Carmen Radecki | The theoretical basis of stigma as applied to genital herpes | Stigma |
| 1994 | Carney, O.; Ross, E.; Bunker, C.; Ikkos, G.; Mindel, A. | A prospective study of the psychological impact on patients with a first episode of genital herpes | Psychological |
| 2004 | Fortenberry, J. Dennis | The effects of stigma on genital herpes care-seeking behaviours | Stigma |
| 2014 | Glynn, Judith R.; Kayuni, Ndoliwe; Gondwe, Levie; Price, Alison J.; Crampin, Amelia C. | Earlier menarche is associated with a higher prevalence of Herpes simplex type-2 (HSV-2) in young women in rural Malawi | Early menarche |
| 2003 | Halton, Kate; Ratcliffe, Amy A.; Morison, Linda; West, Beryl; Shaw, Matthew; Bailey, Robin; Walraven, Gijs | Herpes simplex 2 risk among women in a polygynous setting in rural West Africa | Depression |
| 2016 | Hsu, Pao-Chu; Yolken, Robert H.; Postolache, Teodor T.; Beckie, Theresa M.; Munro, Cindy L.; Groer, Maureen W. | Association of Depressed Mood With Herpes Simplex Virus-2 Immunoglobulin-G Levels in Pregnancy | Depression |
| 1988 | Jacob, Mary; Mathai, Rache | Genital Herpes in Marital Partners | HIV |
| 2015 | Jena, Anupam B.; Goldman, Dana P.; Seabury, Seth A. | Incidence of sexually transmitted infections after human papillomavirus vaccination among adolescent females | HPV |
| 2014 | Kayibanda, Jeanne Françoise; Bitera, Raphaël; Demers, Eric; Moisan, Jocelyne; Alary, Michel | Sexual risk factors associated with intimate partner violence against women in Rwanda: a couples-based analysis | Intimate partner violence |
| 2002 | Korenromp, Eline L.; Bakker, Roel; De Vlas, Sake J.; Robinson, N. Jamie; Hayes, Richard; Habbema, J. Dik F. | Can behavior change explain increases in the proportion of genital ulcers attributable to herpes in sub-Saharan Africa? A simulation modeling study | Care seeking |
| 2018 | Kurscheidt, Fábio A.; Damke, Edilson; Bento, Jaqueline C.; Balani, Valério A.; Takeda, Karen I.; Piva, Sérgio; Piva, João P.; Irie, Mary M. T.; Gimenes, Fabrícia; Consolaro, Marcia E. L. | Effects of Herpes Simplex Virus Infections on Seminal Parameters in Male Partners of Infertile Couples | Infertility |
| 1993 | Leenaars, P. E. M.; Rombouts, R.; Kok, G. | Seeking medical care for a Sexually Transmitted Disease: Determinants of delay-behavior | Care seeking |
| 2011 | Leichliter, Jami S.; Lewis, David A.; Sternberg, Maya; Habel, Melissa A.; Paz-Bailey, Gabriela | Health Care Seeking Among Men With Genital Ulcer Disease in South Africa: Correlates and Relationship to Human Immunodeficiency Virus-1 and Herpes Simplex Virus Type 2 Detection and Shedding: | HIV |
| 2011 | Leichliter, Jami S.; Lewis, David A.; Sternberg, Maya; Habel, Melissa A.; Paz-Bailey, Gabriela | Health Care Seeking Among Men With Genital Ulcer Disease in South Africa: Correlates and Relationship to Human Immunodeficiency Virus-1 and Herpes Simplex Virus Type 2 Detection and Shedding: | Care seeking |
| 2012 | Makasa, Mpundu; Fylkesnes, Knut; Sandøy, Ingvild F. | Risk factors, healthcare-seeking and sexual behaviour among patients with genital ulcers in Zambia | Care seeking |
| 2011 | Merin, Abigail; Pachankis, John E. | The psychological impact of genital herpes stigma | Psychological |
| 2000 | Meyer-Weitz, A.; Reddy, P.; Van den Borne, H. W.; Kok, G.; Pietersen, J. | Health care seeking behaviour of patients with sexually transmitted diseases: determinants of delay behaviour | Care seeking |
| 2001 | Morgan, D.; Mahe, C.; Okongo, J. M.; Mayanja, B.; Whitworth, J. A. | Genital ulceration in rural Uganda: sexual activity, treatment-seeking behavior, and the implications for HIV control | Care seeking |
| 2007 | Morris, Chester N; Ferguson, Alan G | Sexual and treatment‐seeking behaviour for sexually transmitted infection in long‐distance transport workers of East Africa | Care seeking |
| 1994 | Moses, S.; Ngugi, E. N.; Bradley, J. E.; Njeru, E. K.; Eldridge, G.; Muia, E.; Olenja, J.; Plummer, F. A. | Health care-seeking behavior related to the transmission of sexually transmitted diseases in Kenya | Care seeking |
| 2011 | Naumenko, V. A.; Tiulenev, Iu A.; Pushkar', D. Iu; Segal, A. S.; Kovalev, V. A.; Kurilo, L. F.; Shileĭko, L. V.; Klimova, R. R.; Al'khovskiĭ, S. V.; Kushch, A. A. | [Effect of herpes simplex virus on spermatogenesis] | Infertility |
| 2008 | O'Farrell, Nigel; Morison, Linda; Moodley, Prashini; Pillay, Keshree; Vanmali, Trusha; Quigley, Maria; Sturm, A. Wim | Genital ulcers and concomitant complaints in men attending a sexually transmitted infections clinic: implications for sexually transmitted infections management | Infertility |
| 1993 | Randolph, A. G.; Washington, A. E.; Prober, C. G. | Cesarean delivery for women presenting with genital herpes lesions. Efficacy, risks, and costs | Cesarean delivery |
| 2008 | Richards, Julie; Krantz, Elizabeth; Selke, Stacy; Wald, Anna | Healthcare seeking and sexual behavior among patients with symptomatic newly acquired genital herpes | Sexual behavior |
| 2008 | Richards, Julie; Krantz, Elizabeth; Selke, Stacy; Wald, Anna | Healthcare seeking and sexual behavior among patients with symptomatic newly acquired genital herpes | Care seeking |
| 2008 | Richards, Julie; Krantz, Elizabeth; Selke, Stacy; Wald, Anna | Healthcare seeking and sexual behavior among patients with symptomatic newly acquired genital herpes | Care seeking |
| 2010 | Sanchez-Lorente, Segunda; Blasco-Ros, Concepcion; Coe, Christopher L.; Martinez, Manuela | Recovery of immune control over herpes simplex virus type 1 in female victims of intimate partner violence | Intimate partner violence |
| 2002 | Smith, Jennifer S.; Herrero, Rolando; Bosetti, Cristina; Muñoz, Nubia; Bosch, F. Xavier; Eluf-Neto, José; Castellsagué, Xavier; Meijer, Chris J. L. M.; Van den Brule, Adriaan J. C.; Franceschi, Silvia; Ashley, Rhoda; International Agency for Research on Cancer (IARC) Multicentric Cervical Cancer Study Group | Herpes simplex virus-2 as a human papillomavirus cofactor in the etiology of invasive cervical cancer | HPV |
| 2014 | Steiner, Riley J.; Michael, Shannon L.; Hall, Jeffrey E.; Barrios, Lisa C.; Robin, Leah | Youth violence and connectedness in adolescence: what are the implications for later sexually transmitted infections? | Intimate partner violence |
| 2017 | Stoner, Marie C. D.; Pettifor, Audrey; Edwards, Jessie K.; Aiello, Allison E.; Halpern, Carolyn T.; Julien, Aimée; Selin, Amanda; Twine, Rhian; Hughes, James P.; Wang, Jing; Agyei, Yaw; Gomez-Olive, F. Xavier; Wagner, Ryan G.; MacPhail, Catherine; Kahn, Kathleen | The effect of school attendance and school dropout on incident HIV and HSV-2 among young women in rural South Africa enrolled in HPTN 068 | Schooling |
| 1994 | Waitzman, N. J.; Romano, P. S.; Scheffler, R. M. | Estimates of the economic costs of birth defects | Birth defects |
| 2002 | Wald, Anna; Link, Katherine | Risk of human immunodeficiency virus infection in herpes simplex virus type 2-seropositive persons: a meta-analysis | HIV |
| 2003 | Wollenberg, Andreas; Zoch, Claudia; Wetzel, Stefanie; Plewig, Gerd; Przybilla, Bernhard | Predisposing factors and clinical features of eczema herpeticum: a retrospective analysis of 100 cases | Eczema |
| 1976 |  | Editorial: Herpesvirus and cancer of uterine cervix | Partner violence |
| 2013 | Biraro, Samuel; Ruzagira, Eugene; Kamali, Anatoli; Whitworth, James; Grosskurth, Heiner; Weiss, Helen A. | HIV-1 transmission within marriage in rural Uganda: a longitudinal study | Duplicate |
| 2001 | Bogaerts, J.; Ahmed, J.; Akhter, N.; Begum, N.; Rahman, M.; Nahar, S.; Van Ranst, M.; Verhaegen, J. | Sexually transmitted infections among married women in Dhaka, Bangladesh: unexpected high prevalence of herpes simplex type 2 infection | Duplicate |
| 2013 | Burrel, Sonia; Aime, Catherine; Hermet, Laurence; Ait-Arkoub, Zaïna; Agut, Henri; Boutolleau, David | Surveillance of herpes simplex virus resistance to antivirals: a 4-year survey | Duplicate |
| 1994 | Carney, O.; Ross, E.; Bunker, C.; Ikkos, G.; Mindel, A. | A prospective study of the psychological impact on patients with a first episode of genital herpes | Psychological |
| 2014 | Glynn, Judith R.; Kayuni, Ndoliwe; Gondwe, Levie; Price, Alison J.; Crampin, Amelia C. | Earlier menarche is associated with a higher prevalence of Herpes simplex type-2 (HSV-2) in young women in rural Malawi | early menarche |
| 2015 | Jena, Anupam B.; Goldman, Dana P.; Seabury, Seth A. | Incidence of sexually transmitted infections after human papillomavirus vaccination among adolescent females | HPV |
| 2002 | Korenromp, Eline L.; Bakker, Roel; De Vlas, Sake J.; Robinson, N. Jamie; Hayes, Richard; Habbema, J. Dik F. | Can behavior change explain increases in the proportion of genital ulcers attributable to herpes in sub-Saharan Africa? A simulation modeling study | Care seeking |
| 2000 | Meyer-Weitz, A.; Reddy, P.; Van den Borne, H. W.; Kok, G.; Pietersen, J. | Health care seeking behaviour of patients with sexually transmitted diseases: determinants of delay behaviour | Care seeking |
| 2002 | O'Farrell, N. | Genital ulcers, stigma, HIV, and STI control in sub-Saharan Africa | Stigma |
| 1993 | Randolph, A. G.; Washington, A. E.; Prober, C. G. | Cesarean delivery for women presenting with genital herpes lesions. Efficacy, risks, and costs | Cesarean delivery |
| 1994 | Waitzman, N. J.; Romano, P. S.; Scheffler, R. M. | Estimates of the economic costs of birth defects | Birth defects |

**References**

1. World Bank Country and Lending Groups – World Bank Data Help Desk [Internet]. [cited 2018 Mar 19]. Available from: https://datahelpdesk.worldbank.org/knowledgebase/articles/906519-world-bank-country-and-lending-groups

2. WHO | WHO regional offices [Internet]. WHO. [cited 2018 May 27]. Available from: http://www.who.int/about/regions/en/

3. World Population Prospects - Population Division - United Nations [Internet]. [cited 2019 Feb 17]. Available from: https://population.un.org/wpp/

4. Ayoub HH, Amara I, Awad SF, Omori R, Chemaitelly H, Abu-Raddad LJ. Analytic characterization of the herpes simplex virus type 2 epidemic in the United States, 1950-2050. Open Forum Infectious Diseases [Internet]. 2021 Apr 29 [cited 2021 May 13];(ofab218). Available from: https://doi.org/10.1093/ofid/ofab218

5. Ayoub HH, Chemaitelly H, Abu-Raddad LJ. Epidemiological Impact of Novel Preventive and Therapeutic HSV-2 Vaccination in the United States: Mathematical Modeling Analyses. Vaccines (Basel). 2020 Jul 8;8(3).

6. Harfouche M, Abu-Hijleh FM, James C, Looker KJ, Abu-Raddad LJ. The epidemiology of herpes simplex virus type 2 in sub-Saharan Africa: systematic review, meta-analyses, and meta-regressions. medRxiv [Internet]. 2021 Jan 26 [cited 2021 Feb 21];2021.01.25.21250443. Available from: https://www.medrxiv.org/content/10.1101/2021.01.25.21250443v1

7. Bernstein DI, Lovett MA, Bryson YJ. Serologic analysis of first-episode nonprimary genital herpes simplex virus infection. Presence of type 2 antibody in acute serum samples. Am J Med. 1984 Dec;77(6):1055–60.

8. Schiffer JT, Corey L. New concepts in understanding genital herpes. Curr Infect Dis Rep. 2009 Nov;11(6):457–64.

9. Guidelines for the Management of Sexually Transmitted Infections. February 2004 [Internet]. [cited 2018 Mar 19]. Available from: http://apps.who.int/medicinedocs/en/d/Jh2942e/

10. Sexually Transmitted Diseases Treatment Guidelines, 2015 [Internet]. Center for Disease Control (CDC). [cited 2017 Jun 22]. Available from: https://www.cdc.gov/mmwr/preview/mmwrhtml/rr6403a1.htm

11. Langenberg AG, Corey L, Ashley RL, Leong WP, Straus SE. A prospective study of new infections with herpes simplex virus type 1 and type 2. Chiron HSV Vaccine Study Group. N Engl J Med. 1999 Nov 4;341(19):1432–8.

12. Benedetti J, Corey L, Ashley R. Recurrence rates in genital herpes after symptomatic first-episode infection. Ann Intern Med. 1994 Dec 1;121(11):847–54.

13. Corey L, Langenberg AG, Ashley R, Sekulovich RE, Izu AE, Douglas JM, et al. Recombinant glycoprotein vaccine for the prevention of genital HSV-2 infection: two randomized controlled trials. Chiron HSV Vaccine Study Group. JAMA. 1999 Jul 28;282(4):331–40.

14. Corey L, Adams HG, Brown ZA, Holmes KK. Genital herpes simplex virus infections: clinical manifestations, course, and complications. Ann Intern Med. 1983 Jun;98(6):958–72.

15. Corbell C, Stergachis A, Ndowa F, Ndase P, Barnes L, Celum C. Genital ulcer disease treatment policies and access to acyclovir in eight sub-Saharan African countries. Sex Transm Dis. 2010 Aug;37(8):488–93.

16. Global Health Expenditure Database [Internet]. [cited 2021 Jan 30]. Available from: https://apps.who.int/nha/database/Home/Index/en

17. ILOSTAT - the world’s leading source on labour statistics [Internet]. [cited 2018 May 28]. Available from: http://www.ilo.org/ilostat/faces/wcnav_defaultSelection;ILOSTATCOOKIE=Zn6oS2hA4g9ckMXyT3l2JTeORaorY-9iwKzOw5xWoemTpxGz2-Kg!125143199?_afrLoop=966594818050688&_afrWindowMode=0&_afrWindowId=null#!%40%40%3F_afrWindowId%3Dnull%26_afrLoop%3D966594818050688%26_afrWindowMode%3D0%26_adf.ctrl-state%3Dpqa11aqbo_4

18. Patel R, Boselli F, Cairo I, Barnett G, Price M, Wulf HC. Patients’ perspectives on the burden of recurrent genital herpes. International Journal of STD & AIDS. 2001;12(10):640–5.

19. Knight GM, Gomez GB, Dodd PJ, Dowdy D, Zwerling A, Wells WA, et al. The Impact and Cost-Effectiveness of a Four-Month Regimen for First-Line Treatment of Active Tuberculosis in South Africa. PLoS ONE. 2015;10(12):e0145796.

20. Fisman DN. Health related quality of life in genital herpes: a pilot comparison of measures. Sex Transm Infect. 2005 Jun;81(3):267–70.

21. Guidelines For Regulatory Impact Analysis [Internet]. ASPE. 2016 [cited 2020 Dec 28]. Available from: https://aspe.hhs.gov/pdf-report/guidelines-regulatory-impact-analysis

22. Hammitt JK, Robinson LA. The Income Elasticity of the Value per Statistical Life: Transferring Estimates between High and Low Income Populations. Journal of Benefit-Cost Analysis [Internet]. 2011 Jan [cited 2019 Mar 1];2(1):1–29. Available from: https://www.cambridge.org/core/journals/journal-of-benefit-cost-analysis/article/income-elasticity-of-the-value-per-statistical-life-transferring-estimates-between-high-and-low-income-populations/3AE5B0BB1034B7D898E2C9B3C1507794#

23. World Bank Open Data | Data [Internet]. [cited 2020 Jul 28]. Available from: https://data.worldbank.org/

24. Hanley JA. A heuristic approach to the formulas for population attributable fraction. J Epidemiol Community Health. 2001 Jul;55(7):508–14.

25. Looker KJ, Elmes JAR, Gottlieb SL, Schiffer JT, Vickerman P, Turner KME, et al. Effect of HSV-2 infection on subsequent HIV acquisition: an updated systematic review and meta-analysis. The Lancet Infectious Diseases [Internet]. 2017 Dec 1 [cited 2018 Jul 28];17(12):1303–16. Available from: https://www.thelancet.com/journals/laninf/article/PIIS1473-3099(17)30405-X/abstract

26. UNAIDS - KEY POPULATIONS ATLAS [Internet]. [cited 2018 Mar 4]. Available from: http://www.aidsinfoonline.org/kpatlas/#/home

27. AIDSinfo | UNAIDS [Internet]. [cited 2019 May 12]. Available from: http://aidsinfo.unaids.org/

28. Global AIDS Update 2016 [Internet]. [cited 2019 Jan 1]. Available from: http://www.unaids.org/en/resources/documents/2016/Global-AIDS-update-2016

29. AIDS Epidemic Update 2009 [Internet]. [cited 2019 Oct 27]. Available from: https://www.unaids.org/en/resources/documents/2009/20091124_jc1700_epi_update_2009_en.pdf

30. Stover J, Korenromp EL, Blakley M, Komatsu R, Viisainen K, Bollinger L, et al. Long-Term Costs and Health Impact of Continued Global Fund Support for Antiretroviral Therapy. PLOS ONE [Internet]. 2011 Jun 23 [cited 2019 May 27];6(6):e21048. Available from: https://journals.plos.org/plosone/article?id=10.1371/journal.pone.0021048

31. NHANES Questionnaires, Datasets, and Related Documentation [Internet]. [cited 2021 May 15]. Available from: https://wwwn.cdc.gov/nchs/nhanes/

32. Liljeros F, Edling CR, Amaral LA, Stanley HE, Aberg Y. The web of human sexual contacts. Nature. 2001 Jun 21;411(6840):907–8.

33. Garnett GP, Anderson RM. Balancing sexual partnerships in an age and activity stratified model of HIV transmission in heterosexual populations. IMA J Math Appl Med Biol. 1994;11(3):161–92.

34. Awad SF, Abu-Raddad LJ. Could there have been substantial declines in sexual risk behavior across sub-Saharan Africa in the mid-1990s? Epidemics. 2014 Sep;8:9–17.

35. Harfouche M, Abu-Hijleh FM, James C, Looker KJ, Abu-Raddad LJ. Epidemiology of herpes simplex virus type 2 in sub-Saharan Africa: Systematic review, meta-analyses, and meta-regressions. EClinicalMedicine [Internet]. 2021 May 1 [cited 2021 May 15];35. Available from: https://www.thelancet.com/journals/eclinm/article/PIIS2589-5370(21)00156-5/abstract

36. Weaver M. Unit cost of outpatient and inpatient visits from 1995 to 2040. 2021.

37. Foreman KJ, Marquez N, Dolgert A, Fukutaki K, Fullman N, McGaughey M, et al. Forecasting life expectancy, years of life lost, and all-cause and cause-specific mortality for 250 causes of death: reference and alternative scenarios for 2016–40 for 195 countries and territories. The Lancet [Internet]. 2018 Nov 10 [cited 2021 Feb 12];392(10159):2052–90. Available from: https://www.thelancet.com/journals/lancet/article/PIIS0140-6736(18)31694-5/abstract

38. Moses MW, Pedroza P, Baral R, Bloom S, Brown J, Chapin A, et al. Funding and services needed to achieve universal health coverage: applications of global, regional, and national estimates of utilisation of outpatient visits and inpatient admissions from 1990 to 2016, and unit costs from 1995 to 2016. The Lancet Public Health [Internet]. 2019 Jan 1 [cited 2019 Mar 1];4(1):e49–73. Available from: https://www.thelancet.com/journals/lanpub/article/PIIS2468-2667(18)30213-5/abstract

39. Akaike H. A new look at the statistical model identification. IEEE Transactions on Automatic Control. 1974 Dec;19(6):716–23.

40. International Medical Products Price Guide | Management Sciences for Health [Internet]. [cited 2018 Mar 19]. Available from: /resources/international-medical-products-price-guide

41. Looker KJ, Welton NJ, Sabin KM, Dalal S, Vickerman P, Turner KME, et al. Global and regional estimates of the contribution of herpes simplex virus type 2 infection to HIV incidence: a population attributable fraction analysis using published epidemiological data. The Lancet Infectious Diseases [Internet]. 2020 Feb 1 [cited 2020 Jul 28];20(2):240–9. Available from: https://www.thelancet.com/journals/laninf/article/PIIS1473-3099(19)30470-0/abstract

42. Stover J, Bollinger L, Izazola JA, Loures L, DeLay P, Ghys PD, et al. What Is Required to End the AIDS Epidemic as a Public Health Threat by 2030? The Cost and Impact of the Fast-Track Approach. PLOS ONE [Internet]. 2016 May 9 [cited 2019 Aug 3];11(5):e0154893. Available from: https://journals.plos.org/plosone/article?id=10.1371/journal.pone.0154893

43. Resch S, Korenromp E, Stover J, Blakley M, Krubiner C, Thorien K, et al. Economic Returns to Investment in AIDS Treatment in Low and Middle Income Countries. PLOS ONE [Internet]. 2011 Oct 5 [cited 2018 Dec 25];6(10):e25310. Available from: https://journals.plos.org/plosone/article?id=10.1371/journal.pone.0025310

44. Tan-Torres Edejer. Making choices in health: WHO guide to cost-effectiveness analysis [Internet]. [cited 2017 Jun 16]. Available from: http://www.who.int/choice/publications/p_2003_generalised_cea.pdf

45. Hsu PC, Yolken RH, Postolache TT, Beckie TM, Munro CL, Groer MW. Association of Depressed Mood With Herpes Simplex Virus-2 Immunoglobulin-G Levels in Pregnancy. Psychosom Med. 2016;78(8):966–72.

46. Merin A, Pachankis JE. The psychological impact of genital herpes stigma. J Health Psychol. 2011 Jan;16(1):80–90.

47. Bickford J, Barton SE, Mandalia S. Chronic genital herpes and disclosure.... The influence of stigma. Int J STD AIDS. 2007 Sep;18(9):589–92.

48. Fortenberry JD. The effects of stigma on genital herpes care-seeking behaviours. Herpes. 2004 Apr;11(1):8–11.

49. Breitkopf CR. The theoretical basis of stigma as applied to genital herpes. Herpes. 2004 Apr;11(1):4–7.

50. O’Farrell N. Genital ulcers, stigma, HIV, and STI control in sub-Saharan Africa. Sex Transm Infect. 2002 Apr;78(2):143–6.

51. Sanchez-Lorente S, Blasco-Ros C, Coe CL, Martinez M. Recovery of immune control over herpes simplex virus type 1 in female victims of intimate partner violence. Psychosom Med. 2010 Jan;72(1):97–106.

52. Kayibanda JF, Bitera R, Demers E, Moisan J, Alary M. Sexual risk factors associated with intimate partner violence against women in Rwanda: a couples-based analysis. Women Health. 2014;54(4):301–16.

53. Steiner RJ, Michael SL, Hall JE, Barrios LC, Robin L. Youth violence and connectedness in adolescence: what are the implications for later sexually transmitted infections? J Adolesc Health. 2014 Mar;54(3):312-318.e1.

54. Biraro S, Ruzagira E, Kamali A, Whitworth J, Grosskurth H, Weiss HA. HIV-1 transmission within marriage in rural Uganda: a longitudinal study. PLoS ONE. 2013;8(2):e55060.

55. Halton K, Ratcliffe AA, Morison L, West B, Shaw M, Bailey R, et al. Herpes simplex 2 risk among women in a polygynous setting in rural West Africa. AIDS. 2003 Jan 3;17(1):97–103.

56. Bogaerts J, Ahmed J, Akhter N, Begum N, Rahman M, Nahar S, et al. Sexually transmitted infections among married women in Dhaka, Bangladesh: unexpected high prevalence of herpes simplex type 2 infection. Sex Transm Infect. 2001 Apr;77(2):114–9.

57. Latif AS, Katzenstein DA, Bassett MT, Houston S, Emmanuel JC, Marowa E. Genital ulcers and transmission of HIV among couples in Zimbabwe. AIDS. 1989 Aug;3(8):519–23.

58. Jacob M, Mathai R. Genital Herpes in Marital Partners. Indian J Dermatol Venereol Leprol. 1988 Dec;54(6):288–92.

59. Kurscheidt FA, Damke E, Bento JC, Balani VA, Takeda KI, Piva S, et al. Effects of Herpes Simplex Virus Infections on Seminal Parameters in Male Partners of Infertile Couples. Urology. 2018 Mar;113:52–8.

60. Naumenko VA, Tiulenev IA, Pushkar’ DI, Segal AS, Kovalev VA, Kurilo LF, et al. [Effect of herpes simplex virus on spermatogenesis]. Urologiia. 2011 Dec;(6):32–6.

61. Abdulmedzhidova AG, Kurilo LF, Shileĭko LV, Makarova NP, Klimova RR, Kushch AA. [Asymptomatic genital herpes infection and infertility in males]. Urologiia. 2007 Jun;(3):56–9.

62. Bocharova EN, Zavalishina LE, Bragina EE, Klimova RR, Gusak YK, Kurilo LF, et al. Detection of herpes simplex virus genomic DNA in spermatozoa of patients with fertility disorders by in situ hybridization. Dokl Biol Sci. 2007 Feb;412:82–6.

63. Smith JS, Herrero R, Bosetti C, Muñoz N, Bosch FX, Eluf-Neto J, et al. Herpes simplex virus-2 as a human papillomavirus cofactor in the etiology of invasive cervical cancer. J Natl Cancer Inst. 2002 Nov 6;94(21):1604–13.

64. Andrei G, Snoeck R. Herpes simplex virus drug-resistance: new mutations and insights. Curr Opin Infect Dis. 2013 Dec;26(6):551–60.

65. Burrel S, Aime C, Hermet L, Ait-Arkoub Z, Agut H, Boutolleau D. Surveillance of herpes simplex virus resistance to antivirals: a 4-year survey. Antiviral Res. 2013 Nov;100(2):365–72.

66. Bodsworth N, Fife K, Koltun W, Tyring S, Abudalu M, Prichard M, et al. Single-day famciclovir for the treatment of genital herpes: follow-up results of time to next recurrence and assessment of antiviral resistance. Curr Med Res Opin. 2009 Feb;25(2):483–7.

67. Kriesel JD, Spruance SL, Prichard M, Parker JN, Kern ER. Recurrent antiviral-resistant genital herpes in an immunocompetent patient. J Infect Dis. 2005 Jul 1;192(1):156–61.

68. Corey L, Wald A. Maternal and Neonatal HSV Infections. N Engl J Med [Internet]. 2009 Oct 1 [cited 2018 May 21];361(14):1376–85. Available from: https://www.ncbi.nlm.nih.gov/pmc/articles/PMC2780322/

69. Brown ZA, Selke S, Zeh J, Kopelman J, Maslow A, Ashley RL, et al. The acquisition of herpes simplex virus during pregnancy. N Engl J Med. 1997 Aug 21;337(8):509–15.

70. Brown ZA, Vontver LA, Benedetti J, Critchlow CW, Hickok DE, Sells CJ, et al. Genital herpes in pregnancy: risk factors associated with recurrences and asymptomatic viral shedding. Am J Obstet Gynecol. 1985 Sep 1;153(1):24–30.

71. Abdool Karim Q, Kharsany ABM, Leask K, Ntombela F, Humphries H, Frohlich JA, et al. Prevalence of HIV, HSV-2 and pregnancy among high school students in rural KwaZulu-Natal, South Africa: a bio-behavioural cross-sectional survey. Sex Transm Infect. 2014 Dec;90(8):620–6.

72. Stoner MCD, Pettifor A, Edwards JK, Aiello AE, Halpern CT, Julien A, et al. The effect of school attendance and school dropout on incident HIV and HSV-2 among young women in rural South Africa enrolled in HPTN 068. AIDS. 2017 24;31(15):2127–34.

73. Baird SJ, Garfein RS, McIntosh CT, Ozler B. Effect of a cash transfer programme for schooling on prevalence of HIV and herpes simplex type 2 in Malawi: a cluster randomised trial. Lancet. 2012 Apr 7;379(9823):1320–9.

74. Birdthistle I, Floyd S, Nyagadza A, Mudziwapasi N, Gregson S, Glynn JR. Is education the link between orphanhood and HIV/HSV-2 risk among female adolescents in urban Zimbabwe? Soc Sci Med. 2009 May;68(10):1810–8.

75. Szucs TD, Berger K, Fisman DN, Harbarth S. The estimated economic burden of genital herpes in the United States. An analysis using two costing approaches. BMC Infect Dis. 2001;1:5.
