# Supplementary material for: Estimated economic burden of genital herpes and HIV attributable to herpes simplex virus type 2 infections in 90 low- and middle-income countries: A modeling study
Source: PLoS Med. 2022 Dec 15;19(12):e1003938. doi: 10.1371/journal.pmed.1003938 (PMC9754187; doi:10.1371/journal.pmed.1003938)
Supplement: S2 Appendix — (DOCX) [file pmed.1003938.s002.docx]

**Estimated economic burden of genital herpes and HIV attributable to herpes simplex virus type-2 infections in 90 low and middle income countries: A modeling study**

**Supplementary Appendix 2**

Sachin SILVA^1, 2^, Houssein H AYOUB^3^, Christine JOHNSTON^4,5^, Rifat ATUN^1^, Laith J ABU-RADDAD^6,7,8^

1. Harvard TH Chan School of Public Health, Harvard University

Boston, MA Massachusetts, United States of America

1. University of California, San Francisco, Institute for Global Health Sciences,

San Francisco, California, United States of America

1. Mathematics Program, Department of Mathematics, Statistics, and Physics, College of Arts and Sciences, Qatar University

Doha, Qatar

1. Department of Medicine, University of Washington

, Seattle, Washington, United States of America

1. Vaccine and Infectious Diseases Division, Fred Hutchinson Cancer Research Center Eastlake Building, Day Campus,., Seattle, Washington, United States of America
2. Infectious Diseases Epidemiology Group, Weill Cornell Medicine – Qatar

Doha, Qatar

1. World Health Organization Collaborating Centre for Disease Epidemiology Analytics on HIV/AIDS, Sexually Transmitted Infections, and Viral Hepatitis, Weill Cornell Medicine – Qatar

Doha, Qatar

1. Department of Population Health Sciences, Weill Cornell Medicine, Cornell University,

New York, New York, United States of America

***** sas7443@mail.harvard.edu

Table of Contents

[Section 1: Cost of Medicines 3](#_Toc83920666)

[Section 2: Cost of Outpatient Care 5](#_Toc83920667)

[Section 3: Wage Losses 7](#_Toc83920668)

[Section 4: QALY Losses 10](#_Toc83920669)

[Section 5: Spending on ART for HIV cases attributable to HSV-2 (mid-year initiation) 12](#_Toc83920670)

[Section 6: Spending on ART for HIV cases attributable to HSV-2 (full-year initiation) 15](#_Toc83920671)

[Section 7: Wage Losses in HIV cases attributable to HSV-2 17](#_Toc83920672)

[Section 8: Lifetime Costs of Genital Herpes 20](#_Toc83920673)

[Section 9: Populations by Disease States – Africa Region 24](#_Toc83920674)

[Section 10: Results of Sensitivity Analysis 25](#_Toc83920675)

**TABLES**

[Table 1: Cost of medicines by country and scenario considered (2019). 4](#_Toc111477723)

[Table 2: Cost of outpatient care by scenario (2019). 6](#_Toc111477724)

[Table 3: Wage losses by country and scenario (2019). 9](#_Toc111477725)

[Table 4: QALY losses by country and scenario (2019). 11](#_Toc111477726)

[Table 5: Spending on ART for HIV cases attributable to HSV-2 (2019). 13](#_Toc111477727)

[Table 6: Spending on ART for HIV cases attributable to HSV-2 (2019). 16](#_Toc111477728)

[Table 7: Wage losses in HIV cases attributable to HSV-2 (2019). 18](#_Toc111477729)

[Table 8: Lifetime costs of genital herpes. 22](#_Toc111477730)

[Table 9: HSV-2 populations by disease state (2019-2050). 26](#_Toc111477731)

[Table 10: Sensitivity of outpatient costs to parameter uncertainty 29](#_Toc111477732)

[Table 11: Sensitivity of the consumption value of QALY losses to parameter uncertainty 32](#_Toc111477733)

[Table 12: Sensitivity of spending on ART to parameter uncertainty 34](#_Toc111477734)

[Table 13: Sensitivity of wage losses in HIV patients to parameter uncertainty 35](#_Toc111477735)

**FIGURES**

[Figure 1: Tornado Plot – Sensitivity of the total cost of medicines to care-seeking for STIs, unit price of medicines, proportion of population experiencing recurrences, proportion seeking care for recurrences, number of clinic visits made for recurrences and the number of clinical visits made for frequent recurrences. 27](#_Toc111477711)

[Figure 2: Spider Diagram – Sensitivity of the total cost of medicines to care-seeking for STIs, unit price of medicines, proportion of population experiencing recurrences, proportion seeking care for recurrences, number of clinic visits made for recurrences and the number of clinical visits made for frequent recurrences. 27](#_Toc111477712)

[Figure 3: Tornado Plot: Sensitivity of outpatient costs to care-seeking for STIs, proportion with recurrences, number of clinical visits for recurrences, number of clinic visits for freq. recurrences and the proportion with first episodes. 28](#_Toc111477713)

[Figure 4: Spider Diagram: Sensitivity of outpatient costs to care-seeking for STIs, proportion with recurrences, number of clinical visits for recurrences, number of clinic visits for freq. recurrences and the proportion with first episodes. 29](#_Toc111477714)

[Figure 5: Tornado Plot – Sensitivity of wage loses to proportion with first episodes and recurrences, number of recurrent and frequent recurrent episodes per year, absent days due to first episodes, recurrences and freq. recurrences. 30](#_Toc111477715)

[Figure 6: Spider Diagram - Sensitivity of wage loses to proportion with first episodes and recurrences, number of recurrent and frequent recurrent episodes per year, absent days due to first episodes, recurrences and freq. recurrences. 30](#_Toc111477716)

[Figure 7: Tornado Diagram – Sensitivity of the consumption value of QALYs to income elasticity and the value of the US value-of-a-statistical life (VSL). 31](#_Toc111477717)

[Figure 8: Spider Diagram – Sensitivity of the consumption value of QALYs to income elasticity and the value of the US value-of-a-statistical life (VSL). 32](#_Toc111477718)

[Figure 9: Tornado diagram - Sensitivity of spending on ART to relative risk of prevalent infections and relative risk of incident infections 33](#_Toc111477719)

[Figure 10: Spider Diagram - Sensitivity of spending on ART to relative risk of prevalent infections and relative risk of incident infections 33](#_Toc111477720)

[Figure 11: Tornado Diagram - Sensitivity of wage losses in HIV patients to the proportion employed, absent days when not on ART, and absent days when on ART. 34](#_Toc111477721)

[Figure 12: Spider Diagram - Sensitivity of wage losses in HIV patients to the proportion employed, absent days when not on ART, and absent days when on ART. 35](#_Toc111477722)

## Section 1: Cost of Medicines

Table 1: Cost of medicines by country and scenario considered (2019).

| **Country Name** | **Scenario 1** | **Scenario 1**  **OOP Cost** | **Scenario 2** | **Scenario 2**  **OOP Cost** |
| --- | --- | --- | --- | --- |
| Afghanistan | 2,492,072 | 1,940,000 | 550,000 | 429,000 |
| Algeria | 4,000,000 | 1,320,000 | 866,000 | 286,000 |
| Angola | 7,480,000 | 2,768,472 | 1,630,000 | 602,000 |
| Argentina | 6,830,000 | 1,910,000 | 1,475,509 | 413,000 |
| Azerbaijan | 805,000 | 588,000 | 176,000 | 129,000 |
| Bangladesh | 9,256,444 | 6,850,000 | 2,040,000 | 1,505,928 |
| Belarus | 1,070,000 | 268,000 | 230,000 | 57,475 |
| Benin | 1,880,000 | 846,000 | 413,000 | 186,000 |
| Bhutan | 45,547 | 5,921 | 10,035 | 1,305 |
| Bolivia | 2,520,222 | 580,000 | 547,000 | 126,000 |
| Botswana | 762,000 | 22,856 | 165,000 | 4,941 |
| Brazil | 52,949,380 | 14,825,826 | 11,466,732 | 3,210,685 |
| Bulgaria | 493,000 | 202,000 | 106,000 | 43,601 |
| Burkina Faso | 3,450,000 | 1,240,000 | 755,000 | 271,896 |
| Burundi | 2,503,658 | 651,000 | 547,000 | 142,000 |
| Cambodia | 2,210,000 | 1,280,000 | 483,000 | 280,000 |
| Cameroon | 6,196,812 | 4,709,577 | 1,360,000 | 1,030,000 |
| Central African Republic | 1,400,000 | 589,000 | 305,000 | 128,000 |
| Chad | 1,900,000 | 1,180,000 | 418,000 | 259,000 |
| China | 125,168,920 | 45,060,812 | 27,206,758 | 9,794,433 |
| Colombia | 10,398,681 | 1,560,000 | 2,250,000 | 337,000 |
| Congo | 1,550,000 | 0 | 333,000 | 0 |
| Congo, Dem. Rep. | 22,099,092 | 11,491,527 | 4,818,816 | 2,510,000 |
| Cote d'Ivoire | 4,554,978 | 1,780,000 | 995,000 | 388,000 |
| Cuba | 2,160,000 | 238,000 | 465,000 | 51,137 |
| Dominican Republic | 2,140,000 | 965,000 | 466,000 | 210,000 |
| Ecuador | 3,814,248 | 1,530,000 | 827,000 | 331,000 |
| Egypt | 8,474,412 | 5,250,000 | 1,850,000 | 1,150,000 |
| El Salvador | 1,279,739 | 371,000 | 278,000 | 80,610 |
| Ethiopia | 16,646,875 | 5,826,406 | 3,670,000 | 1,280,000 |
| Gabon | 537,000 | 123,000 | 116,000 | 26,710 |
| Gambia, The | 334,000 | 96,869 | 73,353 | 21,272 |
| Ghana | 5,950,000 | 2,260,000 | 1,297,293 | 493,000 |
| Guatemala | 3,527,179 | 2,050,000 | 770,000 | 447,000 |
| Guinea | 1,990,000 | 1,210,000 | 436,000 | 266,000 |
| Haiti | 2,070,000 | 910,000 | 452,000 | 199,000 |
| Honduras | 2,380,000 | 1,210,000 | 518,000 | 264,000 |
| India | 90,794,896 | 57,200,784 | 20,017,900 | 12,611,277 |
| Indonesia | 32,599,966 | 11,409,988 | 7,090,277 | 2,481,597 |
| Iran | 10,594,264 | 3,810,000 | 2,300,000 | 826,404 |
| Iraq | 3,680,000 | 1,880,000 | 805,768 | 411,000 |
| Jamaica | 661,000 | 112,000 | 143,000 | 24,376 |
| Jordan | 1,210,000 | 374,000 | 263,000 | 81,574 |
| Kazakhstan | 1,360,000 | 449,000 | 297,000 | 98,045 |
| Kenya | 11,987,308 | 2,880,000 | 2,622,468 | 629,000 |
| Lebanon | 485,000 | 160,000 | 105,000 | 34,655 |
| Lesotho | 635,000 | 102,000 | 138,000 | 22,110 |
| Liberia | 862,000 | 362,000 | 188,000 | 78,968 |
| Libya | 728,000 | 0 | 158,000 | 0 |
| Madagascar | 6,060,000 | 1,700,000 | 1,320,000 | 370,000 |
| Malawi | 4,804,223 | 528,000 | 1,050,000 | 115,000 |
| Malaysia | 4,450,000 | 1,560,000 | 970,000 | 339,000 |
| Mali | 3,640,000 | 1,240,000 | 799,000 | 272,000 |
| Mexico | 18,765,130 | 7,880,000 | 4,090,000 | 1,720,000 |
| Mongolia | 267,000 | 85,327 | 58,258 | 18,642 |
| Morocco | 3,650,000 | 1,720,000 | 793,000 | 372,000 |
| Mozambique | 7,010,000 | 701,000 | 1,530,000 | 153,000 |
| Myanmar | 7,550,000 | 5,740,000 | 1,640,000 | 1,250,000 |
| Namibia | 674,000 | 53,959 | 147,000 | 11,740 |
| Nepal | 1,670,000 | 851,000 | 368,000 | 188,000 |
| Nicaragua | 1,330,000 | 439,000 | 289,000 | 95,466 |
| Niger | 3,020,000 | 1,480,000 | 665,000 | 326,083 |
| Nigeria | 35,117,552 | 27,040,514 | 7,717,493 | 5,940,000 |
| Pakistan | 9,009,726 | 5,050,000 | 2,000,000 | 1,120,000 |
| Papua New Guinea | 1,660,000 | 166,000 | 361,000 | 36,103 |
| Paraguay | 1,620,000 | 728,921 | 353,000 | 159,000 |
| Peru | 7,650,000 | 2,220,000 | 1,650,000 | 479,000 |
| Philippines | 12,870,921 | 6,950,000 | 2,820,000 | 1,520,000 |
| Russian Federation | 16,045,486 | 6,100,000 | 3,460,000 | 1,310,000 |
| Rwanda | 2,960,000 | 326,000 | 645,000 | 70,933 |
| Senegal | 2,150,000 | 1,200,000 | 471,000 | 264,000 |
| Serbia | 443,000 | 168,000 | 96,357 | 36,616 |
| Sierra Leone | 1,410,000 | 633,000 | 309,000 | 139,000 |
| South Africa | 17,036,700 | 1,362,936 | 3,690,000 | 295,000 |
| Sri Lanka | 2,930,000 | 1,493,958 | 633,000 | 323,000 |
| Sudan | 3,300,000 | 2,180,000 | 723,000 | 477,383 |
| Tajikistan | 636,000 | 432,000 | 140,000 | 95,487 |
| Tanzania | 11,486,603 | 2,760,000 | 2,510,000 | 602,000 |
| Thailand | 11,160,191 | 1,227,621 | 2,390,000 | 263,000 |
| Togo | 1,420,000 | 793,000 | 309,000 | 173,000 |
| Tunisia | 1,110,000 | 433,000 | 240,000 | 93,560 |
| Turkey | 6,760,000 | 1,150,000 | 1,470,000 | 249,000 |
| Uganda | 9,781,304 | 3,720,000 | 2,140,000 | 814,000 |
| Ukraine | 4,899,695 | 2,400,000 | 1,050,000 | 517,000 |
| Uzbekistan | 2,440,000 | 1,470,000 | 537,000 | 322,000 |
| Venezuela | 5,878,080 | 2,230,000 | 1,270,000 | 481,000 |
| Vietnam | 13,981,030 | 6,290,000 | 3,023,287 | 1,360,000 |
| Yemen | 2,470,000 | 0 | 541,000 | 0 |
| Zambia | 4,908,555 | 491,000 | 1,070,000 | 107,000 |
| Zimbabwe | 4,210,000 | 1,009,287 | 917,000 | 220,000 |

## Section 2: Cost of Outpatient Care

Table 2: Cost of outpatient care by scenario (2019).

| Country | Scenario 1 | Scenario 1  OOP Cost | Scenario 2 | Scenario 2  OOP Cost |
| --- | --- | --- | --- | --- |
| Afghanistan | 30,922,742 | 24,119,738 | 4,530,000 | 3,540,000 |
| Algeria | 598,023,104 | 197,347,632 | 86,506,888 | 28,547,274 |
| Angola | 245,052,080 | 90,669,272 | 35,550,608 | 13,153,725 |
| Argentina | 1,152,293,120 | 322,642,080 | 166,258,000 | 46,552,240 |
| Azerbaijan | 110,262,440 | 80,491,584 | 16,072,976 | 11,733,273 |
| Bangladesh | 91,414,992 | 67,647,096 | 13,368,362 | 9,892,588 |
| Belarus | 53,898,280 | 13,474,570 | 7,730,000 | 1,930,000 |
| Benin | 24,297,240 | 10,933,758 | 3,550,000 | 1,600,000 |
| Bhutan | 1,280,000 | 166,000 | 187,000 | 24,306 |
| Bolivia | 126,047,144 | 28,990,844 | 18,252,334 | 4,198,037 |
| Botswana | 162,326,864 | 4,869,806 | 23,439,526 | 703,000 |
| Brazil | 9,327,915,008 | 2,611,816,192 | 1,348,654,208 | 377,623,168 |
| Bulgaria | 72,951,416 | 29,910,080 | 10,516,484 | 4,310,000 |
| Burkina Faso | 97,833,288 | 35,219,984 | 14,271,311 | 5,137,672 |
| Burundi | 11,789,733 | 3,070,000 | 1,720,000 | 446,000 |
| Cambodia | 85,414,208 | 49,540,240 | 12,420,390 | 7,203,826 |
| Cameroon | 135,898,464 | 103,282,832 | 19,793,002 | 15,042,681 |
| Central African Republic | 5,679,749 | 2,390,000 | 824,000 | 346,000 |
| Chad | 30,646,428 | 19,000,786 | 4,490,000 | 2,780,000 |
| China | 15,957,665,792 | 5,744,759,808 | 2,313,613,056 | 832,900,736 |
| Colombia | 860,676,032 | 129,101,408 | 124,264,240 | 18,639,636 |
| Congo | 41,410,220 | 0 | 5,970,000 | 0 |
| Congo, Dem. Rep. | 132,868,136 | 69,091,432 | 19,310,210 | 10,041,309 |
| Cote d'Ivoire | 122,778,104 | 47,883,460 | 17,863,834 | 6,966,895 |
| Cuba | 413,992,672 | 45,539,192 | 59,480,024 | 6,540,000 |
| Dominican Republic | 420,108,288 | 189,048,720 | 60,919,640 | 27,413,838 |
| Ecuador | 441,967,808 | 176,787,120 | 63,950,172 | 25,580,070 |
| Egypt | 292,037,440 | 181,063,216 | 42,462,232 | 26,326,584 |
| El Salvador | 227,453,712 | 65,961,576 | 32,959,506 | 9,558,256 |
| Ethiopia | 230,830,048 | 80,790,512 | 33,801,360 | 11,830,476 |
| Gabon | 37,180,124 | 8,551,429 | 5,372,069 | 1,240,000 |
| Gambia, The | 4,966,283 | 1,440,222 | 726,000 | 210,000 |
| Ghana | 219,117,600 | 83,264,688 | 31,851,610 | 12,103,612 |
| Guatemala | 225,371,472 | 130,715,448 | 32,797,110 | 19,022,324 |
| Guinea | 18,694,396 | 11,403,582 | 2,728,046 | 1,660,000 |
| Haiti | 56,099,496 | 24,683,778 | 8,170,000 | 3,593,486 |
| Honduras | 126,930,720 | 64,734,664 | 18,437,964 | 9,403,361 |
| India | 2,719,050,752 | 1,713,001,984 | 398,483,456 | 251,044,576 |
| Indonesia | 2,666,676,480 | 933,336,768 | 386,804,864 | 135,381,696 |
| Iran | 1,542,874,880 | 555,435,008 | 223,165,696 | 80,339,656 |
| Iraq | 579,318,016 | 295,452,192 | 84,462,080 | 43,075,660 |
| Jamaica | 89,860,776 | 15,276,332 | 13,004,757 | 2,210,000 |
| Jordan | 127,678,504 | 39,580,336 | 18,559,890 | 5,753,566 |
| Kazakhstan | 110,946,576 | 36,612,372 | 16,149,110 | 5,330,000 |
| Kenya | 318,008,384 | 76,322,008 | 46,331,816 | 11,119,636 |
| Lebanon | 77,533,408 | 25,586,026 | 11,204,802 | 3,700,000 |
| Lesotho | 36,482,028 | 5,840,000 | 5,296,077 | 847,000 |
| Liberia | 24,058,094 | 10,104,399 | 3,500,000 | 1,470,000 |
| Libya | 42,870,988 | 0 | 6,196,341 | 0 |
| Madagascar | 60,953,468 | 17,066,972 | 8,857,522 | 2,480,000 |
| Malawi | 114,839,480 | 12,632,343 | 16,718,663 | 1,840,000 |
| Malaysia | 514,733,088 | 180,156,576 | 74,709,896 | 26,148,464 |
| Mali | 87,491,024 | 29,746,948 | 12,784,761 | 4,346,819 |
| Mexico | 3,683,998,720 | 1,547,279,360 | 534,765,952 | 224,601,696 |
| Mongolia | 12,629,439 | 4,040,000 | 1,840,000 | 588,000 |
| Morocco | 272,829,632 | 128,229,928 | 39,507,692 | 18,568,616 |
| Mozambique | 84,782,368 | 8,478,237 | 12,337,577 | 1,230,000 |
| Myanmar | 444,329,184 | 337,690,176 | 64,359,708 | 48,913,376 |
| Namibia | 151,182,288 | 12,094,583 | 21,934,776 | 1,754,782 |
| Nepal | 45,176,612 | 23,040,072 | 6,620,000 | 3,380,000 |
| Nicaragua | 52,786,056 | 17,419,400 | 7,650,000 | 2,530,000 |
| Niger | 33,114,848 | 16,226,276 | 4,855,028 | 2,380,000 |
| Nigeria | 1,390,735,232 | 1,070,866,112 | 203,316,032 | 156,553,344 |
| Pakistan | 106,320,032 | 59,539,220 | 15,659,110 | 8,769,102 |
| Papua New Guinea | 27,213,176 | 2,720,000 | 3,955,395 | 396,000 |
| Paraguay | 190,577,632 | 85,759,936 | 27,659,284 | 12,446,677 |
| Peru | 587,660,544 | 170,421,552 | 84,775,872 | 24,585,002 |
| Philippines | 639,609,536 | 345,389,152 | 93,295,784 | 50,379,724 |
| Russian Federation | 1,043,574,208 | 396,558,208 | 150,237,104 | 57,090,100 |
| Rwanda | 46,623,008 | 5,128,531 | 6,771,881 | 745,000 |
| Senegal | 39,023,032 | 21,852,898 | 5,698,684 | 3,191,263 |
| Serbia | 41,604,256 | 15,809,617 | 6,030,000 | 2,290,000 |
| Sierra Leone | 21,194,516 | 9,537,532 | 3,090,000 | 1,390,000 |
| South Africa | 3,907,432,192 | 312,594,560 | 564,840,704 | 45,187,256 |
| Sri Lanka | 161,499,712 | 82,364,848 | 23,322,608 | 11,894,530 |
| Sudan | 104,252,184 | 68,806,448 | 15,224,502 | 10,048,172 |
| Tajikistan | 11,851,265 | 8,060,000 | 1,740,000 | 1,180,000 |
| Tanzania | 214,762,096 | 51,542,900 | 31,237,654 | 7,497,037 |
| Thailand | 1,923,439,616 | 211,578,352 | 275,922,208 | 30,351,442 |
| Togo | 20,137,062 | 11,276,755 | 2,920,000 | 1,640,000 |
| Tunisia | 90,723,184 | 35,382,040 | 13,095,114 | 5,110,000 |
| Turkey | 439,256,128 | 74,673,544 | 63,584,232 | 10,809,320 |
| Uganda | 214,919,808 | 81,669,528 | 31,344,884 | 11,911,056 |
| Ukraine | 216,392,240 | 106,032,200 | 31,138,180 | 15,257,708 |
| Uzbekistan | 51,052,984 | 30,631,792 | 7,470,000 | 4,479,222 |
| Venezuela | 513,839,776 | 195,259,120 | 74,029,616 | 28,131,254 |
| Vietnam | 1,283,065,472 | 577,379,456 | 185,304,048 | 83,386,816 |
| Yemen | 87,538,048 | 0 | 12,776,712 | 0 |
| Zambia | 255,086,368 | 25,508,638 | 37,078,884 | 3,710,000 |
| Zimbabwe | 159,908,160 | 38,377,956 | 23,231,756 | 5,580,000 |

## Section 3: Wage Losses

Table 3: Wage losses by country and scenario (2019).

| Country | Scenario 1 | Scenario 2 |
| --- | --- | --- |
| Afghanistan | 20,254,716 | 74,672 |
| Algeria | 160,506,176 | 516,000 |
| Angola | 465,207,392 | 1,540,000 |
| Azerbaijan | 1,148,359,936 | 3,585,263 |
| Argentina | 63,490,704 | 221,000 |
| Bangladesh | 297,007,680 | 1,070,000 |
| Bhutan | 112,182,288 | 326,000 |
| Bolivia | 49,413,168 | 176,000 |
| Botswana | 2,520,000 | 9,226 |
| Brazil | 172,703,792 | 561,580 |
| Bulgaria | 92,392,680 | 291,000 |
| Myanmar | 7,408,955,904 | 23,681,886 |
| Burundi | 66,763,496 | 206,000 |
| Belarus | 54,468,124 | 191,000 |
| Cambodia | 22,460,942 | 76,998 |
| Cameroon | 79,567,464 | 271,000 |
| Central African Republic | 203,000,608 | 702,000 |
| Sri Lanka | 17,434,924 | 58,108 |
| Chad | 30,365,020 | 111,000 |
| China | 23,160,160,256 | 76,332,736 |
| Colombia | 1,152,263,936 | 3,620,000 |
| Congo | 49,252,040 | 152,000 |
| Congo, Dem. Rep. | 241,293,248 | 816,000 |
| Cuba | 165,265,408 | 565,000 |
| Benin | 231,114,448 | 686,000 |
| Dominican Republic | 288,245,536 | 952,000 |
| Ecuador | 417,067,232 | 1,340,000 |
| El Salvador | 269,084,928 | 914,000 |
| Ethiopia | 80,954,456 | 265,000 |
| Gabon | 351,847,520 | 1,280,000 |
| Gambia, The | 44,823,840 | 142,000 |
| Ghana | 4,342,920 | 15,508 |
| Guatemala | 246,626,688 | 836,000 |
| Guinea | 276,124,800 | 947,000 |
| Haiti | 34,834,660 | 123,000 |
| Honduras | 30,347,378 | 104,000 |
| India | 105,923,840 | 356,000 |
| Indonesia | 2,630,484,736 | 9,671,570 |
| Iran | 2,372,083,456 | 7,857,153 |
| Iraq | 917,741,376 | 2,950,000 |
| Cote d'Ivoire | 220,204,480 | 769,000 |
| Jamaica | 58,277,624 | 188,000 |
| Kazakhstan | 48,593,580 | 165,000 |
| Jordan | 216,290,912 | 743,000 |
| Kenya | 447,756,736 | 1,550,000 |
| Lebanon | 44,477,096 | 141,000 |
| Lesotho | 13,404,240 | 44,789 |
| Liberia | 12,527,618 | 42,432 |
| Libya | 61,564,852 | 196,000 |
| Madagascar | 92,132,208 | 311,000 |
| Malawi | 49,421,860 | 170,000 |
| Malaysia | 847,425,152 | 2,830,000 |
| Mali | 66,619,100 | 238,000 |
| Mexico | 2,841,575,936 | 9,487,640 |
| Mongolia | 15,893,687 | 54,585 |
| Morocco | 135,087,952 | 439,000 |
| Mozambique | 89,620,672 | 307,000 |
| Namibia | 189,225,664 | 617,000 |
| Nepal | 44,939,336 | 149,000 |
| Nicaragua | 46,526,056 | 172,000 |
| Niger | 45,589,708 | 151,000 |
| Nigeria | 41,639,420 | 154,000 |
| Pakistan | 1,010,775,808 | 3,630,000 |
| Papua New Guinea | 208,164,912 | 801,000 |
| Paraguay | 60,245,164 | 204,000 |
| Peru | 169,624,064 | 565,000 |
| Philippines | 1,058,398,272 | 3,297,910 |
| Russian Federation | 814,309,184 | 2,860,000 |
| Rwanda | 2,883,355,648 | 8,770,321 |
| Senegal | 63,845,608 | 215,000 |
| Serbia | 39,688,724 | 141,000 |
| Sierra Leone | 41,016,516 | 136,000 |
| Vietnam | 13,646,816 | 48,433 |
| South Africa | 1,138,279,040 | 3,630,000 |
| Zimbabwe | 169,004,544 | 533,000 |
| Sudan | 26,618,318 | 94,559 |
| Tajikistan | 7,636,915 | 28,368 |
| Thailand | 311,191,008 | 1,060,000 |
| Togo | 1,469,047,552 | 4,278,424 |
| Tunisia | 24,265,536 | 81,293 |
| Turkey | 40,394,968 | 127,000 |
| Uganda | 810,786,240 | 2,630,000 |
| Ukraine | 169,504,992 | 594,000 |
| Egypt | 226,906,144 | 686,000 |
| Tanzania | 79,161,040 | 285,000 |
| Burkina Faso | 899,114,688 | 2,759,108 |
| Uzbekistan | 764,076,224 | 2,410,000 |
| Venezuela | 24,069,080 | 85,042 |
| Yemen | 139,858,144 | 474,000 |
| Zambia | 137,717,328 | 464,000 |

## Section 4: QALY Losses

Table 4: QALY losses by country and scenario (2019)

| Country | Scenario 1 | Scenario 2 |
| --- | --- | --- |
| Algeria | 34,482 | 339,000 |
| Angola | 64,057 | 629,000 |
| Argentina | 59,007 | 579,000 |
| Azerbaijan | 6,924 | 67,905 |
| Bangladesh | 79,344 | 777,869 |
| Belarus | 9,194 | 90,356 |
| Benin | 15,957 | 156,000 |
| Bhutan | 387 | 3,792 |
| Bolivia | 21,592 | 212,000 |
| Botswana | 6,543 | 64,249 |
| Brazil | 454,000 | 4,460,000 |
| Bulgaria | 4,192 | 41,175 |
| Burkina Faso | 29,257 | 287,000 |
| Burundi | 21,333 | 209,000 |
| Cambodia | 18,985 | 186,000 |
| Cameroon | 52,538 | 515,000 |
| Central African Republic | 11,840 | 116,000 |
| Chad | 16,045 | 157,000 |
| China | 1,076,785 | 10,567,969 |
| Colombia | 89,481 | 879,000 |
| Congo | 13,259 | 130,000 |
| Cote d'Ivoire | 38,588 | 379,000 |
| Cuba | 18,688 | 184,000 |
| Dominican Republic | 189,000 | 1,850,888 |
| DRC | 18,341 | 180,000 |
| Ecuador | 32,751 | 322,000 |
| Egypt | 72,988 | 716,000 |
| El Salvador | 10,907 | 107,000 |
| Ethiopia | 142,000 | 1,390,000 |
| Gabon | 4,594 | 45,106 |
| Gambia | 2,830 | 27,748 |
| Ghana | 50,645 | 497,000 |
| Guatemala | 30,119 | 295,000 |
| Guinea | 16,880 | 166,000 |
| Haiti | 17,551 | 172,000 |
| Honduras | 20,383 | 200,000 |
| India | 774,503 | 7,590,000 |
| Indonesia | 280,000 | 2,750,000 |
| Iran | 91,618 | 899,000 |
| Iraq | 31,566 | 310,000 |
| Jamaica | 5,696 | 55,918 |
| Jordan | 10,399 | 102,000 |
| Kazakhstan | 11,656 | 114,000 |
| Kenya | 103,000 | 1,010,000 |
| Lebanon | 4,201 | 41,248 |
| Lesotho | 5,291 | 51,924 |
| Liberia | 7,366 | 72,274 |
| Libya | 6,269 | 61,552 |
| Madagascar | 51,900 | 509,000 |
| Malawi | 41,013 | 402,000 |
| Malaysia | 38,364 | 376,000 |
| Mali | 30,918 | 303,000 |
| Mexico | 161,000 | 1,580,000 |
| Mongolia | 2,283 | 22,398 |
| Morocco | 31,549 | 310,000 |
| Mozambique | 59,774 | 586,000 |
| Myanmar | 64,614 | 634,000 |
| Namibia | 5,763 | 56,553 |
| Nepal | 14,284 | 140,000 |
| Nicaragua | 11,427 | 112,000 |
| Niger | 25,697 | 252,000 |
| Nigeria | 294,000 | 2,879,542 |
| Pakistan | 76,896 | 753,000 |
| Papua New Guinea | 14,164 | 139,000 |
| Paraguay | 13,892 | 136,000 |
| Peru | 65,871 | 647,000 |
| Philippines | 110,000 | 1,080,000 |
| Russian Federation | 136,000 | 1,334,741 |
| Rwanda | 25,373 | 249,000 |
| Senegal | 18,338 | 180,000 |
| Serbia | 3,805 | 37,340 |
| Sierra Leone | 11,784 | 116,000 |
| South Africa | 144,000 | 1,411,112 |
| Sri Lanka | 25,255 | 248,000 |
| Sudan | 28,085 | 275,000 |
| Tajikistan | 5,462 | 53,530 |
| Tanzania | 98,086 | 962,000 |
| Thailand | 95,620 | 940,000 |
| Togo | 12,027 | 118,000 |
| Tunisia | 9,598 | 94,246 |
| Turkey | 58,237 | 572,000 |
| Uganda | 83,721 | 821,000 |
| Ukraine | 41,658 | 409,000 |
| Uzbekistan | 20,928 | 205,000 |
| Venezuela | 50,561 | 497,000 |
| Vietnam | 120,000 | 1,180,000 |
| Yemen | 21,110 | 207,000 |
| Zambia | 41,950 | 412,000 |
| Zimbabwe | 35,884 | 352,000 |

## Section 5: Spending on ART for HIV cases attributable to HSV-2 (mid-year initiation)

Table 5: Spending on ART for HIV cases attributable to HSV-2 (2019).

| **Country** | **Total HIV Population** | **Population Attributable to HSV-2** | **Total HIV Cost** | **Total Attributable Cost** |
| --- | --- | --- | --- | --- |
| Afghanistan | 638.73 | 92.906 | $12,951 | $3,646 |
| Algeria | 419.621 | 76.595 | $186,000 | $65,900 |
| Angola | 38714.078 | 16085.89 | $2,190,000 | $1,760,472 |
| Argentina | 10183.807 | 2791.85 | $5,680,000 | $3,020,000 |
| Azerbaijan | 279.778 | 43.152 | $116,000 | $34,633 |
| Bangladesh | 502.349 | 58.367 | $19,353 | $4,373 |
| Belarus | 1809.461 | 427.494 | $909,913 | $420,000 |
| Benin | 2552.69 | 740.147 | $348,000 | $195,000 |
| Bhutan | 61.081 | 7.006 | $4,583 | $1,020 |
| Bolivia | 1225.994 | 425.235 | $612,000 | $410,000 |
| Botswana | 8516.342 | 3648.939 | $1,460,000 | $1,210,000 |
| Brazil | 50169.379 | 18500.438 | $28,800,946 | $20,615,854 |
| Bulgaria | 130.89 | 22.139 | $52,238 | $17,204 |
| Burkina Faso | 1807.729 | 536.289 | $254,000 | $146,000 |
| Burundi | 1142.949 | 426.13 | $201,000 | $144,000 |
| Cambodia | 658.583 | 160.277 | $112,000 | $52,523 |
| Cameroon | 26286.949 | 9307.995 | $3,418,344 | $2,340,000 |
| Central African Republic | 5987.277 | 2454.623 | $578,000 | $459,000 |
| Chad | 7422.91 | 1907.289 | $903,000 | $449,000 |
| China | 22143.824 | 4020.312 | $2,510,000 | $880,000 |
| Colombia | 7120.068 | 2482.242 | $2,670,000 | $1,800,000 |
| Congo | 5261.646 | 2242.016 | $276,000 | $228,000 |
| Congo, Dem. Rep. | 9818.929 | 3981.168 | $1,132,689 | $885,000 |
| Cote d'Ivoire | 11861.637 | 3615.729 | $1,570,000 | $924,473 |
| Cuba | 1331.136 | 456.472 | $842,000 | $561,000 |
| Dominican Republic | 2490.861 | 804.003 | $995,000 | $620,000 |
| Ecuador | 3124.173 | 1088.464 | $1,690,000 | $1,140,000 |
| Egypt | 376.108 | 64.26 | $79,539 | $26,289 |
| El Salvador | 795.49 | 267.29 | $331,000 | $215,000 |
| Ethiopia | 18420.559 | 5311.334 | $2,859,028 | $1,580,000 |
| Gabon | 2135.914 | 913.704 | $228,000 | $189,000 |
| Gambia, The | 1959.121 | 543.993 | $119,000 | $63,572 |
| Ghana | 15501.37 | 4840.952 | $1,460,000 | $882,000 |
| Guatemala | 1700.601 | 550.709 | $821,000 | $511,000 |
| Guinea | 8811.388 | 2685.718 | $1,050,000 | $620,000 |
| Haiti | 7058.877 | 2004.239 | $4,170,000 | $2,290,000 |
| Honduras | 450.977 | 169.477 | $180,000 | $130,000 |
| India | 60017.82 | 7708.328 | $6,815,021 | $1,690,000 |
| Indonesia | 12142.374 | 2695.405 | $345,000 | $148,000 |
| Iran | 2671.583 | 581.96 | $441,000 | $187,000 |
| Iraq | 211.492 | 35.154 | $16,772 | $5,400 |
| Jamaica | 839.178 | 300.916 | $307,000 | $213,000 |
| Jordan | 32.962 | 6.467 | $18,298 | $6,921 |
| Kazakhstan | 2266.527 | 352.067 | $940,750 | $283,000 |
| Kenya | 56673.879 | 20948.83 | $8,796,270 | $6,292,074 |
| Lebanon | 75.841 | 14.056 | $31,576 | $11,380 |
| Lesotho | 17868.689 | 7481.059 | $2,436,071 | $1,970,000 |
| Liberia | 2180.582 | 661.653 | $151,000 | $88,632 |
| Libya | 138.098 | 24.909 | $31,030 | $10,885 |
| Madagascar | 4678.515 | 1739.986 | $128,000 | $91,477 |
| Malawi | 28582.387 | 11719.77 | $4,735,976 | $3,740,000 |
| Malaysia | 4247.739 | 1044.468 | $431,000 | $205,000 |
| Mali | 5412.741 | 1759.847 | $409,000 | $256,000 |
| Mexico | 14396.016 | 3830.857 | $7,430,000 | $3,820,000 |
| Mongolia | 21.393 | 3.335 | $1,388 | $419 |
| Morocco | 620.875 | 119.054 | $287,000 | $107,000 |
| Mozambique | 1.50E+05 | 61141.359 | $18,838,052 | $14,768,125 |
| Myanmar | 7460.978 | 1850.025 | $1,150,000 | $551,000 |
| Namibia | 5270.551 | 2160.732 | $940,000 | $744,000 |
| Nepal | 1197.371 | 135.324 | $153,000 | $33,533 |
| Nicaragua | 1200.625 | 398.41 | $589,000 | $376,000 |
| Niger | 1488.881 | 426.747 | $190,000 | $105,000 |
| Nigeria | 118000 | 36046.918 | $16,083,469 | $9,473,204 |
| Pakistan | 8741.824 | 793.174 | $213,000 | $37,359 |
| Papua New Guinea | 5904.578 | 1714.374 | $742,300 | $417,000 |
| Paraguay | 947.286 | 339.834 | $347,000 | $241,000 |
| Peru | 4569.056 | 1623.627 | $2,930,000 | $2,020,000 |
| Philippines | 41012.059 | 8845.224 | $3,660,000 | $1,530,000 |
| Russian Federation | 110000 | 24981.332 | $31,715,548 | $14,015,862 |
| Rwanda | 4252.102 | 1578.919 | $776,000 | $556,000 |
| Senegal | 1940.75 | 520.012 | $285,000 | $147,000 |
| Serbia | 76.294 | 9.278 | $40,192 | $9,495 |
| Sierra Leone | 6497.756 | 1950.466 | $586,000 | $339,000 |
| South Africa | 324000 | 136000 | $47,506,748 | $38,533,704 |
| Sri Lanka | 176.324 | 44.827 | $18,234 | $8,975 |
| Sudan | 12520.833 | 2064.864 | $578,000 | $185,000 |
| Tajikistan | 427.077 | 59.82 | $174,000 | $46,761 |
| Tanzania | 46728.582 | 16588.646 | $7,350,684 | $5,040,000 |
| Thailand | 21993.119 | 6180.816 | $3,570,000 | $1,956,739 |
| Togo | 3366.432 | 1048.678 | $451,891 | $273,000 |
| Tunisia | 244.435 | 45.66 | $32,308 | $11,739 |
| Turkey | 350.164 | 54.186 | $75,465 | $22,769 |
| Uganda | 54982.164 | 21975.055 | $9,686,904 | $7,470,000 |
| Ukraine | 30320.51 | 6904.665 | $13,068,939 | $5,806,426 |
| Uzbekistan | 2145.225 | 314.5 | $993,000 | $280,000 |
| Venezuela | 5135.127 | 1765.786 | $1,710,000 | $1,140,000 |
| Vietnam | 14815.55 | 3725.933 | $2,100,000 | $1,020,000 |
| Yemen | 674.389 | 109.931 | $107,000 | $33,889 |
| Zambia | 39436.797 | 16217.912 | $7,030,000 | $5,585,282 |
| Zimbabwe | 24203.699 | 10247.44 | $4,320,000 | $3,520,000 |

##

## Section 6: Spending on ART for HIV cases attributable to HSV-2 (full-year initiation)

Table 6: Spending on ART for HIV cases attributable to HSV-2 (2019).

| Country | **Total HIV Population** | **Population Attributable to HSV-2** | **Total HIV Cost** | **Total Attributable Cost** |
| --- | --- | --- | --- | --- |
| Afghanistan | 638.73 | 92.906 | $25,903 | $3,768 |
| Algeria | 419.621 | 76.595 | $372,000 | $67,831 |
| Angola | 38714.078 | 16085.89 | $4,380,000 | $1,820,000 |
| Argentina | 10183.807 | 2791.85 | $11,353,607 | $3,110,000 |
| Azerbaijan | 279.778 | 43.152 | $232,000 | $35,822 |
| Bangladesh | 502.349 | 58.367 | $38,707 | $4,497 |
| Belarus | 1809.461 | 427.494 | $1,819,826 | $430,000 |
| Benin | 2552.69 | 740.147 | $696,000 | $202,000 |
| Bhutan | 61.081 | 7.006 | $9,165 | $1,051 |
| Bolivia | 1225.994 | 425.235 | $1,220,000 | $425,000 |
| Botswana | 8516.342 | 3648.939 | $2,930,000 | $1,260,000 |
| Brazil | 50169.379 | 18500.438 | $57,601,892 | $21,241,246 |
| Bulgaria | 130.89 | 22.139 | $104,000 | $17,672 |
| Burkina Faso | 1807.729 | 536.289 | $508,000 | $151,000 |
| Burundi | 1142.949 | 426.13 | $403,000 | $150,000 |
| Cambodia | 658.583 | 160.277 | $224,000 | $54,598 |
| Cameroon | 26286.949 | 9307.995 | $6,836,688 | $2,420,000 |
| Central African Republic | 5987.277 | 2454.623 | $1,160,000 | $474,000 |
| Chad | 7422.91 | 1907.289 | $1,810,000 | $464,000 |
| China | 22143.824 | 4020.312 | $5,030,000 | $913,000 |
| Colombia | 7120.068 | 2482.242 | $5,331,453 | $1,858,684 |
| Congo | 5261.646 | 2242.016 | $552,000 | $235,000 |
| Congo, Dem. Rep. | 9818.929 | 3981.168 | $2,265,378 | $919,000 |
| Cote d'Ivoire | 11861.637 | 3615.729 | $3,130,000 | $956,000 |
| Cuba | 1331.136 | 456.472 | $1,680,000 | $577,000 |
| Dominican Republic | 2490.861 | 804.003 | $1,990,000 | $642,000 |
| Ecuador | 3124.173 | 1088.464 | $3,379,071 | $1,180,000 |
| Egypt | 376.108 | 64.26 | $159,000 | $27,179 |
| El Salvador | 795.49 | 267.29 | $662,000 | $222,000 |
| Ethiopia | 18420.559 | 5311.334 | $5,718,056 | $1,648,729 |
| Gabon | 2135.914 | 913.704 | $456,949 | $195,000 |
| Gambia, The | 1959.121 | 543.993 | $238,327 | $66,177 |
| Ghana | 15501.37 | 4840.952 | $2,930,000 | $914,000 |
| Guatemala | 1700.601 | 550.709 | $1,640,000 | $531,000 |
| Guinea | 8811.388 | 2685.718 | $2,110,000 | $642,000 |
| Haiti | 7058.877 | 2004.239 | $8,339,555 | $2,370,000 |
| Honduras | 450.977 | 169.477 | $360,000 | $135,000 |
| India | 60017.82 | 7708.328 | $13,630,042 | $1,750,000 |
| Indonesia | 12142.374 | 2695.405 | $689,383 | $153,000 |
| Iran | 2671.583 | 581.96 | $883,000 | $192,000 |
| Iraq | 211.492 | 35.154 | $33,545 | $5,576 |
| Jamaica | 839.178 | 300.916 | $614,000 | $220,000 |
| Jordan | 32.962 | 6.467 | $36,597 | $7,180 |
| Kazakhstan | 2266.527 | 352.067 | $1,881,500 | $292,000 |
| Kenya | 56673.879 | 20948.83 | $17,592,540 | $6,502,875 |
| Lebanon | 75.841 | 14.056 | $63,153 | $11,704 |
| Lesotho | 17868.689 | 7481.059 | $4,872,142 | $2,039,813 |
| Liberia | 2180.582 | 661.653 | $302,000 | $91,592 |
| Libya | 138.098 | 24.909 | $62,060 | $11,194 |
| Madagascar | 4678.515 | 1739.986 | $255,000 | $94,886 |
| Malawi | 28582.387 | 11719.77 | $9,471,952 | $3,880,000 |
| Malaysia | 4247.739 | 1044.468 | $861,000 | $212,000 |
| Mali | 5412.741 | 1759.847 | $817,000 | $266,000 |
| Mexico | 14396.016 | 3830.857 | $14,851,931 | $3,950,000 |
| Mongolia | 21.393 | 3.335 | $2,776 | $433 |
| Morocco | 620.875 | 119.054 | $574,000 | $110,000 |
| Mozambique | 1.50E+05 | 61141.359 | $37,676,104 | $15,388,640 |
| Myanmar | 7460.978 | 1850.025 | $2,300,000 | $570,000 |
| Namibia | 5270.551 | 2160.732 | $1,880,000 | $770,000 |
| Nepal | 1197.371 | 135.324 | $306,000 | $34,573 |
| Nicaragua | 1200.625 | 398.41 | $1,180,000 | $391,000 |
| Niger | 1488.881 | 426.747 | $381,000 | $109,000 |
| Nigeria | 1.18E+05 | 36046.918 | $32,166,938 | $9,828,684 |
| Pakistan | 8741.824 | 793.174 | $425,000 | $38,599 |
| Papua New Guinea | 5904.578 | 1714.374 | $1,484,600 | $431,000 |
| Paraguay | 947.286 | 339.834 | $694,000 | $249,000 |
| Peru | 4569.056 | 1623.627 | $5,850,000 | $2,080,000 |
| Philippines | 41012.059 | 8845.224 | $7,318,013 | $1,580,000 |
| Russian Federation | 1.10E+05 | 24981.332 | $63,431,096 | $14,356,817 |
| Rwanda | 4252.102 | 1578.919 | $1,550,000 | $576,000 |
| Senegal | 1940.75 | 520.012 | $570,000 | $153,000 |
| Serbia | 76.294 | 9.278 | $80,385 | $9,775 |
| Sierra Leone | 6497.756 | 1950.466 | $1,170,000 | $352,000 |
| South Africa | 3.24E+05 | 1.36E+05 | $95,013,496 | $39,884,776 |
| Sri Lanka | 176.324 | 44.827 | $36,468 | $9,271 |
| Sudan | 12520.833 | 2064.864 | $1,160,000 | $191,000 |
| Tajikistan | 427.077 | 59.82 | $348,000 | $48,703 |
| Tanzania | 46728.582 | 16588.646 | $14,701,368 | $5,218,986 |
| Thailand | 21993.119 | 6180.816 | $7,140,000 | $2,010,000 |
| Togo | 3366.432 | 1048.678 | $903,782 | $282,000 |
| Tunisia | 244.435 | 45.66 | $64,616 | $12,070 |
| Turkey | 350.164 | 54.186 | $151,000 | $23,356 |
| Uganda | 54982.164 | 21975.055 | $19,373,808 | $7,740,000 |
| Ukraine | 30320.51 | 6904.665 | $26,137,878 | $5,950,000 |
| Uzbekistan | 2145.225 | 314.5 | $1,990,000 | $291,000 |
| Venezuela | 5135.127 | 1765.786 | $3,420,000 | $1,180,000 |
| Vietnam | 14815.55 | 3725.933 | $4,210,000 | $1,060,000 |
| Yemen | 674.389 | 109.931 | $214,000 | $34,872 |
| Zambia | 39436.797 | 16217.912 | $14,061,591 | $5,780,000 |
| Zimbabwe | 24203.699 | 10247.44 | $8,630,075 | $3,653,829 |
|  | | | | |

## Section 7: Wage Losses in HIV cases attributable to HSV-2

Table 7: Wage losses in HIV cases attributable to HSV-2 (2019).

| **Country** | **Attributable**  **Percent** | **ART**  **Coverage** | **Daily**  **Wage** | **Scenario 1: Total Wage Loss** | **Scenario 2: Total Wage Loss** |
| --- | --- | --- | --- | --- | --- |
| Afghanistan | 13.091 | 0.1 | $2.52 | $955 | $907 |
| Algeria | 16.428 | 0.67 | $15.15 | $3,234 | $1,647 |
| Angola | 37.395 | 0.27 | $11.76 | $698,000 | $594,000 |
| Argentina | 24.673 | 0.67 | $41.77 | $325,000 | $166,000 |
| Azerbaijan | 13.881 | 0.52 | $17.03 | $2,297 | $1,517 |
| Bangladesh | 10.457 | 0.19 | $7.68 | $1,735 | $1,561 |
| Belarus | 21.263 | 0.63 | $23.66 | $29,097 | $16,093 |
| Benin | 26.095 | 0.65 | $5.14 | $10,765 | $5,722 |
| Bhutan | 10.323 | 0.37 | $11.47 | $278 | $218 |
| Bolivia | 31.216 | 0.6 | $13.53 | $16,945 | $9,898 |
| Botswana | 38.562 | 0.82 | $28.74 | $257,000 | $81,178 |
| Brazil | 33.188 | 0.69 | $34.15 | $1,730,000 | $842,342 |
| Bulgaria | 15.223 | 0.5 | $35.18 | $2,470 | $1,675 |
| Burkina Faso | 26.7 | 0.67 | $3.44 | $5,144 | $2,620 |
| Burundi | 33.555 | 0.84 | $1.56 | $1,601 | $459 |
| Cambodia | 21.903 | 0.84 | $5.98 | $2,304 | $660 |
| Cameroon | 31.868 | 0.62 | $6.06 | $163,000 | $92,127 |
| Central African Republic | 36.898 | 0.46 | $2.45 | $19,595 | $13,954 |
| Chad | 23.125 | 0.58 | $3.11 | $17,739 | $10,716 |
| China | 16.34 | 0.56 | $38.86 | $474,000 | $296,000 |
| Colombia | 31.376 | 0.45 | $24.50 | $200,000 | $144,000 |
| Congo | 38.349 | 0.25 | $6.98 | $58,436 | $50,454 |
| Congo, Dem. Rep. | 36.491 | 0.55 | $2.45 | $29,799 | $18,860 |
| Cote d'Ivoire | 27.434 | 0.63 | $8.96 | $93,263 | $51,582 |
| Cuba | 30.863 | 0.76 | $0.53 | $629 | $251 |
| Dominican Republic | 29.05 | 0.48 | $30.32 | $78,403 | $54,520 |
| Ecuador | 31.356 | 0.65 | $22.92 | $70,651 | $37,553 |
| Egypt | 15.377 | 0.32 | $10.44 | $2,400 | $1,962 |
| El Salvador | 30.241 | 0.5 | $15.26 | $12,935 | $8,772 |
| Ethiopia | 25.95 | 0.74 | $3.66 | $51,138 | $21,754 |
| Gabon | 38.5 | 0.51 | $27.08 | $77,902 | $52,145 |
| Gambia, The | 24.99 | 0.29 | $3.26 | $6,461 | $5,412 |
| Ghana | 28.106 | 0.45 | $8.71 | $138,000 | $99,706 |
| Guatemala | 29.145 | 0.58 | $17.51 | $28,829 | $17,416 |
| Guinea | 27.432 | 0.57 | $4.03 | $32,616 | $20,021 |
| Haiti | 25.554 | 0.71 | $3.44 | $18,601 | $8,604 |
| Honduras | 33.822 | 0.48 | $9.33 | $5,087 | $3,537 |
| India | 11.559 | 0.56 | $8.38 | $196,000 | $122,000 |
| Indonesia | 19.979 | 0.14 | $15.44 | $166,000 | $154,000 |
| Iran | 19.605 | 0.25 | $0.53 | $1,159 | $1,001 |
| Iraq | 14.96 | 0.12 | $21.67 | $3,069 | $2,883 |
| Jamaica | 32.273 | 0.44 | $19.86 | $19,764 | $14,396 |
| Jordan | 17.658 | 0.84 | $16.37 | $254 | $73 |
| Kazakhstan | 13.98 | 0.52 | $32.97 | $36,285 | $23,964 |
| Kenya | 33.267 | 0.74 | $6.98 | $384,000 | $163,000 |
| Lebanon | 16.68 | 0.63 | $28.52 | $1,153 | $638 |
| Lesotho | 37.68 | 0.65 | $5.54 | $117,000 | $62,394 |
| Liberia | 27.309 | 0.33 | $2.67 | $6,277 | $5,088 |
| Libya | 16.233 | 0.34 | $28.66 | $2,522 | $2,027 |
| Madagascar | 33.472 | 0.13 | $2.45 | $17,066 | $15,936 |
| Malawi | 36.903 | 0.79 | $1.93 | $56,966 | $20,452 |
| Malaysia | 22.13 | 0.5 | $41.77 | $138,000 | $93,821 |
| Mali | 29.262 | 0.36 | $3.77 | $23,157 | $18,278 |
| Mexico | 23.949 | 0.62 | $35.25 | $392,000 | $221,000 |
| Mongolia | 14.03 | 0.32 | $14.45 | $172 | $141 |
| Morocco | 17.258 | 0.7 | $12.28 | $3,975 | $1,886 |
| Mozambique | 36.76 | 0.6 | $2.30 | $414,000 | $242,000 |
| Myanmar | 22.316 | 0.76 | $5.65 | $27,009 | $10,791 |
| Namibia | 36.897 | 0.85 | $19.16 | $98,554 | $26,714 |
| Nepal | 10.172 | 0.63 | $4.55 | $1,770 | $979 |
| Nicaragua | 29.865 | 0.59 | $7.57 | $8,945 | $5,315 |
| Niger | 25.796 | 0.61 | $2.60 | $3,236 | $1,857 |
| Nigeria | 27.5 | 0.65 | $8.01 | $817,000 | $435,000 |
| Pakistan | 8.166 | 0.12 | $6.17 | $19,710 | $18,512 |
| Papua New Guinea | 26.131 | 0.62 | $10.77 | $53,537 | $30,173 |
| Paraguay | 32.287 | 0.44 | $20.82 | $23,396 | $17,042 |
| Peru | 31.982 | 0.77 | $25.35 | $105,000 | $40,714 |
| Philippines | 19.411 | 0.44 | $14.71 | $430,000 | $313,000 |
| Russian Federation | 20.37 | 0.36 | $41.99 | $3,660,000 | $2,890,000 |
| Rwanda | 33.419 | 0.87 | $3.55 | $13,096 | $3,136 |
| Senegal | 24.115 | 0.7 | $5.87 | $8,303 | $3,940 |
| Serbia | 10.945 | 0.66 | $26.38 | $688 | $358 |
| Sierra Leone | 27.016 | 0.43 | $2.37 | $15,416 | $11,352 |
| South Africa | 37.78 | 0.7 | $22.77 | $8,410,409 | $3,990,000 |
| Sri Lanka | 22.881 | 0.51 | $15.33 | $2,164 | $1,449 |
| Sudan | 14.842 | 0.22 | $2.71 | $21,249 | $18,740 |
| Tajikistan | 12.606 | 0.51 | $4.33 | $815 | $545 |
| Tanzania | 31.95 | 0.75 | $4.51 | $195,000 | $80,435 |
| Thailand | 25.293 | 0.8 | $27.26 | $420,000 | $145,000 |
| Togo | 28.036 | 0.64 | $3.07 | $9,201 | $4,991 |
| Tunisia | 16.812 | 0.2 | $12.90 | $2,268 | $2,027 |
| Turkey | 13.927 | 0.27 | $35.92 | $7,183 | $6,110 |
| Uganda | 35.971 | 0.84 | $3.41 | $180,000 | $51,490 |
| Ukraine | 20.495 | 0.54 | $12.94 | $275,000 | $177,000 |
| Uzbekistan | 13.194 | 0.58 | $7.16 | $6,734 | $4,068 |
| Venezuela | 30.948 | 0.4 | $0.53 | $3,197 | $2,429 |
| Vietnam | 22.634 | 0.7 | $9.89 | $100,000 | $47,523 |
| Yemen | 14.671 | 0.24 | $3.99 | $1,650 | $1,435 |
| Zambia | 37.011 | 0.85 | $5.87 | $227,000 | $61,436 |
| Zimbabwe | 38.104 | 0.85 | $5.65 | $138,000 | $37,359 |

## Section 8: Lifetime Costs of Genital Herpes

Table 8: Lifetime costs of genital herpes.

| ***Country*** | ***Cost of Medicines + Outpatient*** | ***OOP Cost of Medicines + Wages*** | ***Cost Medicines + Outpatient + Wage Losses*** | ***OOP Cost of Medicines + Outpatient + Wages Losses*** | ***Cost of Medicines + Outpatient + Wages Losses per case*** | ***Cost of Medicines + Outpatient per case*** | ***OOP Cost of Medicines + Wage Losses per case*** | ***OOP Cost of Medicines + Outpatient + Wages Losses per case*** | ***Lifetime OOP Cost of Medicines + Wage Losses*** | ***Lifetime Cost of Medicines + Outpatient Care*** | ***Lifetime OOP Cost of Medicines + Outpatient + Wage Losses*** |
| --- | --- | --- | --- | --- | --- | --- | --- | --- | --- | --- | --- |
| Afghanistan | 23,210,238 | 32,358,972 | 53,999,210 | 48,895,157 | 28.54 | 12.27 | 17.10 | 25.84 | 934.00 | 669.93 | 1411.29 |
| Algeria | 412,782,571 | 244,985,104 | 656,707,675 | 380,143,136 | 220.17 | 138.39 | 82.13 | 127.45 | 4450.81 | 7499.29 | 6906.31 |
| Angola | 173,886,019 | 709,254,128 | 880,910,147 | 771,365,040 | 157.08 | 31.01 | 126.47 | 137.55 | 6553.76 | 1606.77 | 7127.68 |
| Argentina | 794,510,001 | 1,746,648,480 | 2,539,618,481 | 1,967,573,248 | 499.08 | 156.13 | 343.24 | 386.66 | 16568.43 | 7536.59 | 18664.08 |
| Azerbaijan | 76,209,174 | 96,974,784 | 172,710,958 | 152,134,364 | 284.63 | 125.59 | 159.81 | 250.72 | 7274.71 | 5716.95 | 11412.59 |
| Bangladesh | 70,112,061 | 456,972,337 | 521,570,589 | 503,341,453 | 74.49 | 10.01 | 65.26 | 71.89 | 3299.76 | 506.27 | 3634.58 |
| Belarus | 37,751,356 | 170,675,712 | 208,211,068 | 179,898,051 | 262.47 | 47.59 | 215.15 | 226.78 | 7762.78 | 1717.03 | 8182.23 |
| Benin | 18,163,185 | 75,788,608 | 93,270,793 | 83,282,541 | 65.55 | 12.76 | 53.26 | 58.53 | 2651.80 | 635.52 | 2914.01 |
| Bhutan | 911,669 | 3,834,767 | 4,741,669 | 3,948,767 | 137.36 | 26.41 | 111.09 | 114.39 | 5497.89 | 1307.06 | 5661.33 |
| Bolivia | 88,363,256 | 262,933,120 | 350,830,376 | 282,789,770 | 186.51 | 46.98 | 139.78 | 150.34 | 7190.47 | 2416.48 | 7733.50 |
| Botswana | 111,770,832 | 140,425,194 | 252,177,648 | 143,759,929 | 443.98 | 196.78 | 247.23 | 253.10 | 11780.60 | 9376.72 | 12060.36 |
| Brazil | 6,430,765,852 | 11,271,388,793 | 17,690,232,604 | 13,060,081,145 | 447.46 | 162.66 | 285.10 | 330.34 | 13707.63 | 7820.73 | 15882.93 |
| Bulgaria | 50,345,076 | 101,617,696 | 151,800,772 | 122,096,816 | 413.40 | 137.10 | 276.73 | 332.51 | 8816.78 | 4368.15 | 10593.63 |
| Burkina Faso | 69,817,296 | 83,788,104 | 152,606,400 | 107,925,132 | 58.68 | 26.85 | 32.22 | 41.50 | 1696.22 | 1413.40 | 2184.86 |
| Burundi | 10,090,000 | 34,662,148 | 44,228,148 | 36,762,148 | 23.49 | 5.36 | 18.41 | 19.52 | 955.13 | 278.04 | 1013.00 |
| Cambodia | 60,303,540 | 121,962,080 | 181,235,620 | 155,904,692 | 109.18 | 36.33 | 73.47 | 93.92 | 3515.59 | 1738.27 | 4494.00 |
| Cameroon | 98,112,768 | 312,333,104 | 406,655,872 | 383,106,408 | 87.15 | 21.03 | 66.94 | 82.11 | 3096.60 | 972.73 | 3798.28 |
| Central African Republic | 5,020,000 | 26,971,872 | 31,517,872 | 28,606,054 | 29.99 | 4.78 | 25.66 | 27.22 | 1187.17 | 220.96 | 1259.10 |
| Chad | 22,539,944 | 47,105,108 | 68,697,052 | 60,131,273 | 47.61 | 15.62 | 32.64 | 41.67 | 1582.25 | 757.11 | 2019.79 |
| China | 11,031,588,592 | 35,234,757,372 | 46,230,101,744 | 39,169,885,180 | 493.11 | 117.67 | 375.83 | 417.80 | 16261.97 | 5091.43 | 18078.15 |
| Colombia | 597,727,346 | 1,752,316,542 | 2,348,789,746 | 1,840,721,502 | 303.03 | 77.12 | 226.07 | 237.48 | 11238.18 | 3833.42 | 11805.16 |
| Congo | 29,592,780 | 74,844,568 | 104,437,348 | 74,844,568 | 90.58 | 25.67 | 64.91 | 64.91 | 3142.43 | 1242.49 | 3142.43 |
| Congo, Dem. Rep. | 108,810,032 | 375,975,460 | 475,540,400 | 423,311,584 | 28.64 | 6.55 | 22.65 | 25.50 | 1209.99 | 350.18 | 1362.33 |
| Cote d'Ivoire | 87,785,472 | 252,614,304 | 338,969,776 | 285,423,236 | 99.00 | 25.64 | 73.78 | 83.36 | 3280.89 | 1140.13 | 3707.00 |
| Cuba | 285,122,752 | 351,377,336 | 636,309,088 | 382,549,438 | 395.47 | 177.20 | 218.38 | 237.76 | 8761.50 | 7109.46 | 9538.77 |
| Dominican Republic | 289,495,648 | 438,849,216 | 727,568,864 | 568,348,256 | 453.31 | 180.37 | 273.43 | 354.11 | 14209.97 | 9373.89 | 18403.15 |
| Ecuador | 305,768,144 | 635,060,272 | 939,598,416 | 756,139,528 | 329.65 | 107.28 | 222.80 | 265.28 | 12040.29 | 5797.14 | 14335.86 |
| Egypt | 206,906,733 | 413,200,222 | 615,879,757 | 537,255,198 | 96.76 | 32.51 | 64.92 | 84.41 | 3164.73 | 1584.71 | 4114.88 |
| El Salvador | 156,827,968 | 123,330,344 | 279,860,312 | 168,511,752 | 292.13 | 163.70 | 128.74 | 175.90 | 6517.97 | 8288.31 | 8905.79 |
| Ethiopia | 171,642,258 | 539,519,120 | 706,471,378 | 594,903,304 | 56.02 | 13.61 | 42.78 | 47.17 | 2196.03 | 698.64 | 2421.46 |
| Gabon | 25,893,366 | 68,217,703 | 94,011,806 | 74,077,703 | 234.94 | 64.71 | 170.48 | 185.13 | 8614.48 | 3269.79 | 9354.48 |
| Gambia, The | 3,669,000 | 6,679,206 | 10,270,236 | 7,666,206 | 40.61 | 14.51 | 26.41 | 30.31 | 1379.75 | 757.92 | 1583.64 |
| Ghana | 154,914,880 | 376,657,792 | 529,752,672 | 433,705,244 | 118.55 | 34.67 | 84.29 | 97.05 | 4113.27 | 1691.74 | 4736.26 |
| Guatemala | 157,263,024 | 421,329,168 | 576,942,192 | 510,894,520 | 217.22 | 59.21 | 158.63 | 192.35 | 8965.94 | 3346.58 | 10871.90 |
| Guinea | 14,413,040 | 53,924,480 | 67,360,520 | 61,744,480 | 44.91 | 9.61 | 35.95 | 41.16 | 1890.59 | 505.32 | 2164.76 |
| Haiti | 40,100,160 | 46,857,984 | 86,225,144 | 63,771,654 | 55.24 | 25.69 | 30.02 | 40.85 | 1407.24 | 1204.29 | 1915.19 |
| Honduras | 88,869,704 | 161,962,232 | 249,856,936 | 206,311,680 | 140.37 | 49.93 | 90.99 | 115.91 | 5225.56 | 2867.30 | 6656.45 |
| India | 1,937,205,472 | 4,044,603,932 | 5,935,756,000 | 5,218,989,980 | 86.21 | 28.14 | 58.74 | 75.80 | 2826.12 | 1353.60 | 3646.71 |
| Indonesia | 1,852,946,256 | 3,614,270,242 | 5,458,038,608 | 4,253,623,522 | 223.39 | 75.84 | 147.93 | 174.10 | 6568.03 | 3367.27 | 7729.90 |
| Iran | 1,065,185,830 | 1,397,781,680 | 2,459,897,510 | 1,778,181,552 | 310.85 | 134.60 | 176.63 | 224.70 | 8497.81 | 6475.80 | 10810.45 |
| Iraq | 399,962,624 | 336,207,696 | 734,660,320 | 538,679,024 | 265.32 | 144.44 | 121.42 | 194.54 | 6443.68 | 7665.59 | 10324.19 |
| Jamaica | 62,077,400 | 88,657,064 | 150,644,056 | 99,119,782 | 304.82 | 125.61 | 179.40 | 200.57 | 8336.50 | 5837.19 | 9320.32 |
| Jordan | 88,447,912 | 74,156,400 | 162,303,312 | 101,274,242 | 178.95 | 97.52 | 81.76 | 111.66 | 4575.29 | 5457.05 | 6248.41 |
| Kazakhstan | 77,111,200 | 329,100,616 | 405,850,816 | 354,187,612 | 396.73 | 75.38 | 321.70 | 346.22 | 14409.01 | 3376.16 | 15507.39 |
| Kenya | 227,563,090 | 682,875,008 | 908,118,098 | 735,175,084 | 100.57 | 25.20 | 75.63 | 81.42 | 3800.27 | 1266.41 | 4091.33 |
| Lebanon | 53,486,556 | 67,720,728 | 121,078,284 | 85,242,592 | 334.17 | 147.62 | 186.90 | 235.26 | 9367.60 | 7398.63 | 11791.35 |
| Lesotho | 25,503,466 | 20,453,752 | 45,875,530 | 24,453,752 | 96.40 | 53.59 | 42.98 | 51.39 | 1527.14 | 1904.16 | 1825.79 |
| Liberia | 17,176,948 | 19,331,196 | 36,217,144 | 26,254,034 | 55.94 | 26.53 | 29.86 | 40.55 | 1488.26 | 1322.41 | 2021.23 |
| Libya | 29,944,276 | 93,560,176 | 123,504,452 | 93,560,176 | 227.39 | 55.13 | 172.26 | 172.26 | 8526.72 | 2729.01 | 8526.72 |
| Madagascar | 46,636,636 | 141,397,152 | 186,663,788 | 153,090,015 | 40.99 | 10.24 | 31.05 | 33.62 | 1578.84 | 520.75 | 1709.40 |
| Malawi | 82,559,704 | 75,541,304 | 157,676,008 | 84,197,171 | 43.56 | 22.81 | 20.87 | 23.26 | 1100.98 | 1203.27 | 1227.14 |
| Malaysia | 356,199,264 | 1,289,181,392 | 1,644,130,656 | 1,412,598,136 | 492.25 | 106.65 | 385.98 | 422.93 | 18172.05 | 5020.92 | 19911.71 |
| Mali | 62,897,660 | 102,256,184 | 164,158,844 | 122,645,188 | 59.76 | 22.90 | 37.22 | 44.65 | 1938.29 | 1192.24 | 2324.77 |
| Mexico | 2,538,850,441 | 4,325,025,158 | 6,857,535,625 | 5,385,002,310 | 487.27 | 180.40 | 307.32 | 382.63 | 14542.25 | 8536.51 | 18106.27 |
| Mongolia | 8,868,721 | 24,225,378 | 33,025,443 | 26,995,378 | 164.96 | 44.30 | 121.01 | 134.84 | 5651.01 | 2068.79 | 6297.16 |
| Morocco | 189,808,928 | 206,680,368 | 395,109,296 | 294,508,760 | 144.66 | 69.50 | 75.67 | 107.83 | 3696.62 | 3394.86 | 5267.49 |
| Mozambique | 63,732,180 | 136,777,456 | 199,945,636 | 142,586,674 | 37.88 | 12.08 | 25.91 | 27.02 | 1306.88 | 608.95 | 1362.38 |
| Myanmar | 310,410,204 | 292,190,799 | 597,987,964 | 523,489,519 | 105.93 | 54.99 | 51.76 | 92.74 | 2410.00 | 2560.27 | 4317.76 |
| Namibia | 104,107,792 | 68,342,516 | 172,406,904 | 76,627,699 | 340.96 | 205.89 | 135.16 | 151.54 | 6348.30 | 9670.52 | 7117.91 |
| Nepal | 32,313,186 | 71,409,096 | 103,037,282 | 87,205,421 | 81.58 | 25.58 | 56.54 | 69.05 | 3014.67 | 1364.16 | 3681.55 |
| Nicaragua | 37,228,612 | 69,639,864 | 106,515,476 | 81,572,206 | 106.84 | 37.34 | 69.85 | 81.82 | 3612.61 | 1931.26 | 4231.61 |
| Niger | 25,133,338 | 64,485,764 | 88,429,102 | 75,610,400 | 38.55 | 10.96 | 28.11 | 32.96 | 1502.51 | 585.60 | 1761.72 |
| Nigeria | 981,532,498 | 1,558,160,116 | 2,517,926,610 | 2,292,174,132 | 94.80 | 36.95 | 58.66 | 86.30 | 2567.72 | 1617.48 | 3777.31 |
| Pakistan | 80,178,640 | 320,515,520 | 396,634,160 | 361,349,960 | 57.69 | 11.66 | 46.62 | 52.56 | 2480.21 | 620.44 | 2796.19 |
| Papua New Guinea | 19,976,387 | 91,696,992 | 111,540,379 | 93,561,444 | 89.88 | 16.10 | 73.89 | 75.39 | 3602.12 | 784.73 | 3675.36 |
| Paraguay | 131,855,088 | 258,383,280 | 389,652,368 | 317,133,068 | 320.97 | 108.61 | 212.84 | 261.23 | 11018.54 | 5622.85 | 13523.87 |
| Peru | 408,537,232 | 1,610,173,088 | 2,016,930,320 | 1,726,865,384 | 354.53 | 71.81 | 283.03 | 303.54 | 15136.59 | 3840.49 | 16233.57 |
| Philippines | 448,694,286 | 1,243,305,312 | 1,686,406,158 | 1,480,006,800 | 173.72 | 46.22 | 128.07 | 152.46 | 6292.22 | 2270.79 | 7490.14 |
| Russian Federation | 727,347,855 | 4,386,422,087 | 5,108,868,751 | 4,657,913,063 | 428.50 | 61.01 | 367.91 | 390.68 | 12972.44 | 2151.06 | 13775.35 |
| Rwanda | 34,321,014 | 97,296,928 | 131,355,942 | 100,806,928 | 59.20 | 15.47 | 43.85 | 45.43 | 2280.49 | 804.43 | 2362.76 |
| Senegal | 28,475,640 | 61,293,268 | 88,801,908 | 76,270,826 | 54.92 | 17.61 | 37.91 | 47.17 | 1955.93 | 908.68 | 2433.87 |
| Serbia | 28,855,200 | 62,471,636 | 91,191,836 | 73,301,332 | 274.45 | 86.84 | 188.02 | 220.61 | 6952.87 | 3211.48 | 8158.17 |
| Sierra Leone | 15,658,877 | 21,252,924 | 36,401,801 | 27,792,924 | 34.24 | 14.73 | 19.99 | 26.15 | 866.31 | 638.29 | 1132.90 |
| South Africa | 2,689,650,767 | 1,730,944,421 | 4,419,499,215 | 1,945,020,501 | 347.45 | 211.45 | 136.08 | 152.91 | 5745.39 | 8927.55 | 6455.96 |
| Sri Lanka | 112,952,368 | 258,033,641 | 369,784,784 | 314,435,749 | 169.47 | 51.77 | 118.26 | 144.10 | 5698.74 | 2494.58 | 6944.39 |
| Sudan | 74,102,512 | 42,209,452 | 114,561,964 | 89,368,112 | 45.97 | 29.74 | 16.94 | 35.86 | 863.50 | 1515.95 | 1828.24 |
| Tajikistan | 8,642,000 | 11,956,955 | 20,250,955 | 17,482,354 | 41.97 | 17.91 | 24.78 | 36.23 | 1321.80 | 955.35 | 1932.62 |
| Tanzania | 156,389,648 | 475,191,712 | 629,361,360 | 510,507,192 | 72.88 | 18.11 | 55.03 | 59.12 | 2748.60 | 904.59 | 2952.87 |
| Thailand | 1,325,427,586 | 2,233,190,288 | 3,557,631,874 | 2,378,000,832 | 430.84 | 160.51 | 270.45 | 287.98 | 10693.47 | 6346.71 | 11386.88 |
| Togo | 14,934,886 | 37,517,468 | 51,814,354 | 45,242,897 | 48.79 | 14.06 | 35.33 | 42.60 | 1677.33 | 667.71 | 2022.72 |
| Tunisia | 63,016,364 | 61,734,776 | 124,403,140 | 85,962,888 | 150.33 | 76.15 | 74.60 | 103.88 | 3578.65 | 3652.94 | 4983.10 |
| Turkey | 306,288,666 | 1,233,110,880 | 1,538,475,546 | 1,284,255,496 | 304.32 | 60.59 | 243.92 | 254.03 | 12088.59 | 3002.65 | 12589.97 |
| Uganda | 155,156,944 | 260,629,040 | 412,795,984 | 316,598,080 | 55.96 | 21.03 | 35.33 | 42.92 | 1860.63 | 1107.67 | 2260.20 |
| Ukraine | 152,080,752 | 346,731,536 | 496,882,288 | 419,320,504 | 136.61 | 41.81 | 95.33 | 115.28 | 3283.97 | 1440.39 | 3971.47 |
| Uzbekistan | 36,964,388 | 121,506,456 | 157,290,844 | 142,503,090 | 85.07 | 19.99 | 65.71 | 77.07 | 3214.11 | 977.79 | 3769.51 |
| Venezuela | 356,529,750 | 1,368,105,152 | 1,722,834,902 | 1,501,790,840 | 394.54 | 81.65 | 313.30 | 343.92 | 15853.12 | 4131.34 | 17402.22 |
| Vietnam | 889,868,602 | 1,166,209,821 | 2,051,019,706 | 1,561,591,965 | 196.74 | 85.36 | 111.87 | 149.79 | 5377.35 | 4103.15 | 7200.44 |
| Yemen | 61,984,396 | 36,584,292 | 98,568,688 | 36,584,292 | 52.88 | 33.25 | 19.63 | 19.63 | 999.39 | 1693.26 | 999.39 |
| Zambia | 178,717,824 | 212,959,528 | 391,282,352 | 230,436,310 | 106.10 | 48.46 | 57.74 | 62.48 | 3054.65 | 2563.50 | 3305.34 |
| Zimbabwe | 112,933,696 | 210,120,800 | 322,242,496 | 236,413,686 | 102.06 | 35.77 | 66.55 | 74.88 | 2994.04 | 1609.21 | 3368.69 |

## Section 9: Populations by Disease States – Africa Region

Table 9: HSV-2 populations by disease state (2019-2050).

| ***Year*** | ***HSV-2 Seroprevalence*** | ***Total 15-49 Population*** | ***Seroincident Population*** | ***Seroprevalent Population*** | ***Prevalent Population with First Episodes*** | ***Prevalent Population with Recurrent Episodes*** | ***Prevalent Population with Frequent Recurrences*** | ***Incident Population with First Episodes*** | ***Incident Population with Recurrent Episodes*** | ***Incident Population with Frequent Recurrences*** |
| --- | --- | --- | --- | --- | --- | --- | --- | --- | --- | --- |
| 2019 | 31.39 | 524,319,790 | 7,129,164 | 164,590,896 | 172,820 | 103,445,376 | 34,481,792 | 1,497,124 | 940,943 | 313,648 |
| 2020 | 30.98 | 538,306,421 | 7,158,711 | 166,778,961 | 175,118 | 104,820,576 | 34,940,192 | 1,503,329 | 944,843 | 314,948 |
| 2021 | 30.57 | 552,568,591 | 7,181,504 | 168,893,942 | 177,339 | 106,149,840 | 35,383,280 | 1,508,116 | 947,851 | 315,950 |
| 2022 | 30.14 | 567,105,909 | 7,197,114 | 170,929,386 | 179,476 | 107,429,120 | 35,809,708 | 1,511,394 | 949,911 | 316,637 |
| 2023 | 29.71 | 581,917,645 | 7,205,102 | 172,878,570 | 181,523 | 108,654,184 | 36,218,060 | 1,513,071 | 950,965 | 316,988 |
| 2024 | 29.27 | 597,002,725 | 7,205,022 | 174,734,492 | 183,471 | 109,820,632 | 36,606,876 | 1,513,055 | 950,955 | 316,985 |
| 2025 | 28.82 | 612,359,715 | 7,196,414 | 176,489,873 | 185,314 | 110,923,888 | 36,974,628 | 1,511,247 | 949,819 | 316,606 |
| 2026 | 28.37 | 627,986,806 | 7,178,819 | 178,137,144 | 187,044 | 111,959,200 | 37,319,732 | 1,507,552 | 947,496 | 315,832 |
| 2027 | 27.90 | 643,881,799 | 7,151,776 | 179,668,444 | 188,652 | 112,921,616 | 37,640,540 | 1,501,873 | 943,927 | 314,642 |
| 2028 | 27.43 | 660,042,106 | 7,114,814 | 181,075,668 | 190,129 | 113,806,056 | 37,935,352 | 1,494,111 | 939,049 | 313,016 |
| 2029 | 26.96 | 676,464,732 | 7,067,466 | 182,350,458 | 191,468 | 114,607,264 | 38,202,424 | 1,484,168 | 932,799 | 310,933 |
| 2030 | 26.47 | 693,146,327 | 7,009,256 | 183,484,336 | 192,659 | 115,319,904 | 38,439,968 | 1,471,944 | 925,117 | 308,372 |
| 2031 | 25.98 | 710,083,025 | 6,939,722 | 184,468,369 | 193,692 | 115,938,368 | 38,646,124 | 1,457,342 | 915,939 | 305,313 |
| 2032 | 25.48 | 727,270,527 | 6,858,398 | 185,293,436 | 194,558 | 116,456,928 | 38,818,976 | 1,440,264 | 905,206 | 301,735 |
| 2033 | 24.97 | 744,704,067 | 6,764,829 | 185,950,200 | 195,248 | 116,869,696 | 38,956,564 | 1,420,614 | 892,856 | 297,619 |
| 2034 | 24.45 | 762,378,520 | 6,658,564 | 186,429,318 | 195,751 | 117,170,824 | 39,056,940 | 1,398,298 | 878,831 | 292,944 |
| 2035 | 23.93 | 780,288,170 | 6,539,171 | 186,721,068 | 196,057 | 117,354,192 | 39,118,064 | 1,373,226 | 863,073 | 287,691 |
| 2036 | 23.40 | 798,426,944 | 6,406,234 | 186,815,791 | 196,157 | 117,413,728 | 39,137,908 | 1,345,309 | 845,527 | 281,842 |
| 2037 | 22.86 | 816,788,236 | 6,259,356 | 186,703,638 | 196,039 | 117,343,232 | 39,114,412 | 1,314,465 | 826,141 | 275,380 |
| 2038 | 22.31 | 835,364,925 | 6,098,175 | 186,374,675 | 195,693 | 117,136,480 | 39,045,492 | 1,280,617 | 804,868 | 268,289 |
| 2039 | 21.75 | 854,149,526 | 5,922,354 | 185,819,107 | 195,110 | 116,787,304 | 38,929,104 | 1,243,694 | 781,662 | 260,554 |
| 2040 | 21.19 | 873,133,882 | 5,731,610 | 185,026,953 | 194,278 | 116,289,448 | 38,763,148 | 1,203,638 | 756,487 | 252,162 |
| 2041 | 20.62 | 892,309,524 | 5,525,699 | 183,988,552 | 193,188 | 115,636,800 | 38,545,600 | 1,160,397 | 729,309 | 243,103 |
| 2042 | 20.04 | 911,667,379 | 5,304,447 | 182,694,273 | 191,829 | 114,823,352 | 38,274,448 | 1,113,934 | 700,107 | 233,369 |
| 2043 | 19.45 | 931,198,032 | 5,067,745 | 181,134,873 | 190,192 | 113,843,272 | 37,947,756 | 1,064,226 | 668,866 | 222,955 |
| 2044 | 18.86 | 950,891,318 | 4,815,587 | 179,301,207 | 188,266 | 112,690,808 | 37,563,600 | 1,011,273 | 635,585 | 211,862 |
| 2045 | 18.25 | 970,736,735 | 4,548,060 | 177,184,727 | 186,044 | 111,360,600 | 37,120,200 | 955,093 | 600,276 | 200,092 |
| 2046 | 17.64 | 990,723,265 | 4,265,381 | 174,777,427 | 183,516 | 109,847,608 | 36,615,872 | 895,730 | 562,966 | 187,655 |
| 2047 | 17.02 | 1,010,839,374 | 3,967,915 | 172,071,994 | 180,676 | 108,147,248 | 36,049,084 | 833,262 | 523,705 | 174,568 |
| 2048 | 16.40 | 1,031,073,046 | 3,656,214 | 169,062,010 | 177,515 | 106,255,480 | 35,418,492 | 767,805 | 482,565 | 160,855 |
| 2049 | 15.76 | 1,051,411,849 | 3,331,036 | 165,742,171 | 174,029 | 104,168,960 | 34,722,984 | 699,518 | 439,647 | 146,549 |
| 2050 | 15.12 | 1,071,842,914 | 2,993,403 | 162,108,480 | 170,214 | 101,885,176 | 33,961,728 | 628,615 | 395,084 | 131,695 |

## Section 10: Results of Sensitivity Analysis

Figure 1: Tornado Plot – Sensitivity of the total cost of medicines to care-seeking for STIs, unit price of medicines, proportion of population experiencing recurrences, proportion seeking care for recurrences, number of clinic visits made for recurrences and the number of clinical visits made for frequent recurrences.


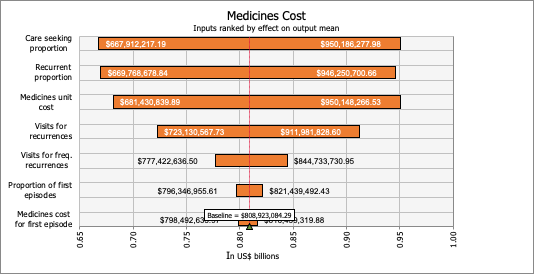


Figure 2: Spider Diagram – Sensitivity of the total cost of medicines to care-seeking for STIs, unit price of medicines, proportion of population experiencing recurrences, proportion seeking care for recurrences, number of clinic visits made for recurrences and the number of clinical visits made for frequent recurrences.


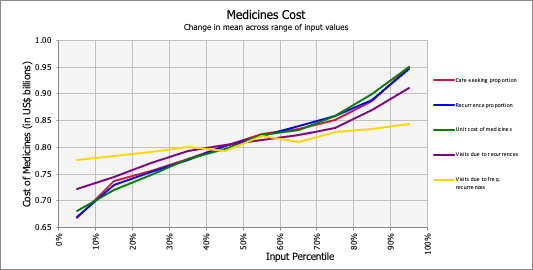


Table : Sensitivity of the cost of medicines to parameter uncertainty

| *Rank* | *Name* | *Range of Mean* |
| --- | --- | --- |
| *1* | *Care-seeking Proportion* | *US$667,912,217-US$950,186,277* |
| *2* | *Proportion with recurrences* | *US$669,768,678- US$946,250,700* |
| *3* | *Unit cost of medicines* | *US$681,430,839- US$950,148,266* |
| *4* | *Number of visits due to recurrences* | *US$723,130,567- US$911,981,828* |
| *5* | *Number of visits due to freq. recurrences* | *US$777,422,636-US$844,733,730* |
| *6* | *Proportion of first episodes* | *US$796,346,955- US$821,439,492* |
| *7* | *Medicines for first episode* | *US$798,492,633 - US$816,439,319* |

Figure 3: Tornado Plot: Sensitivity of outpatient costs to care-seeking for STIs, proportion with recurrences, number of clinical visits for recurrences, number of clinic visits for freq. recurrences and the proportion with first episodes.


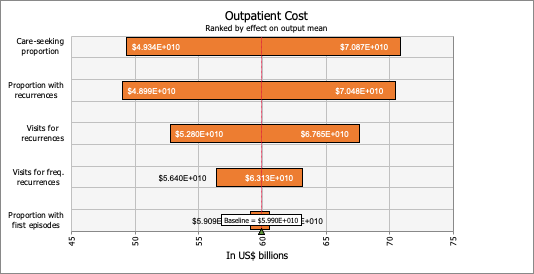


Figure 4: Spider Diagram: Sensitivity of outpatient costs to care-seeking for STIs, proportion with recurrences, number of clinical visits for recurrences, number of clinic visits for freq. recurrences and the proportion with first episodes.


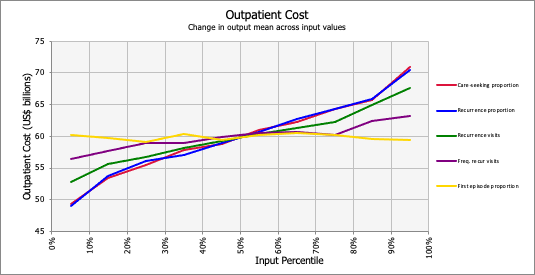


Table 10: Sensitivity of outpatient costs to parameter uncertainty

| *Rank* | *Name* | *Range of Mean* |
| --- | --- | --- |
| *1* | *Care-seeking proportion* | *US$49,342,255,803-US$70,868,016,632* |
| *2* | *Proportion with recurrences* | *US$48,990,324,403- US$70,484,350,847* |
| *4* | *Number of visits due to recurrences* | *US$52,798,448,726- US$67,645,748,933* |
| *5* | *Number of visits due to freq. recurrences* | *US$56,400,070,701- US$63,132,839,936* |
| *6* | *Proportion of first episodes* | *US$59,094,432,623- US$60,523,946,670* |

Figure 5: Tornado Plot – Sensitivity of wage loses to proportion with first episodes and recurrences, number of recurrent and frequent recurrent episodes per year, absent days due to first episodes, recurrences and freq. recurrences.


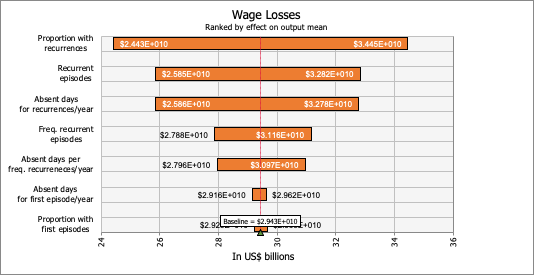


Figure 6: Spider Diagram - Sensitivity of wage loses to proportion with first episodes and recurrences, number of recurrent and frequent recurrent episodes per year, absent days due to first episodes, recurrences and freq. recurrences.


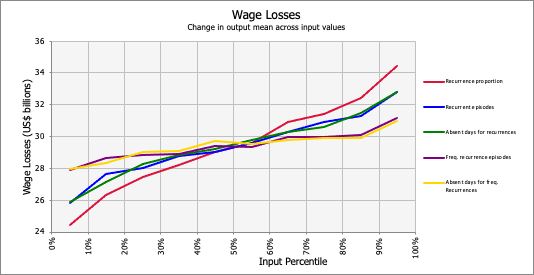


Table : Sensitivity of wage losses to parameter uncertainty

| *Rank* | *Name* | *Range of Mean* |
| --- | --- | --- |
| *1* | *Proportion with recurrences* | *US$24,427,759,908- US$34,447,003,617* |
| *2* | *Number of recurrent episodes* | *US$25,852,011,945- US$32,824,588,522* |
| *3* | *Number of absent days due to recurrences* | *US$25,863,051,673- US$3,277,5670,234* |
| *4* | *Number freq. recurrent episodes* | *US$27,877,236,206- US$31,157,422,056* |
| *5* | *Number of absent days due to freq. recurrences* | *US$27,958,208,187- US$30,966,960,126* |
| *6* | *Number of absent days due to first episodes* | *US$29,156,618,354- US$29,618,610,065* |
| *7* | *Proportion with first episodes* | *US$29,231,972,333- US$29,653,904,395* |

Figure 7: Tornado Diagram – Sensitivity of the consumption value of QALYs to income elasticity and the value of the US value-of-a-statistical life (VSL).


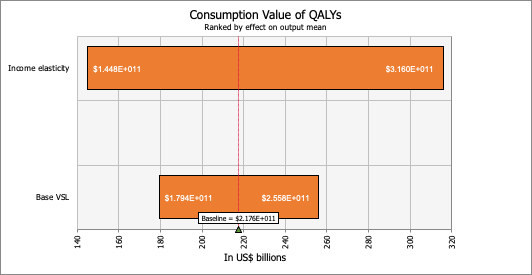


Figure 8: Spider Diagram – Sensitivity of the consumption value of QALYs to income elasticity and the value of the US value-of-a-statistical life (VSL).


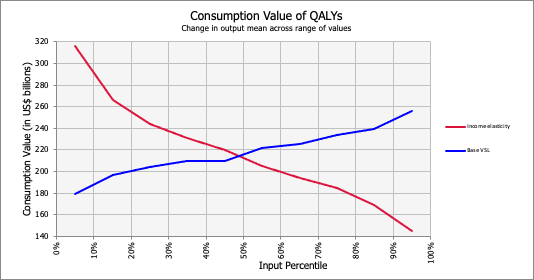


Table 11: Sensitivity of the consumption value of QALY losses to parameter uncertainty

| *Rank* | *Name* | *Range of Mean* |
| --- | --- | --- |
| *1* | *Income elasticity* | *US$144,845,820,413-US$316,026,610,248* |
| *2* | *US VSL* | *US$179,410,107,407- US$255,835,404,040* |

Figure 9: Tornado diagram - Sensitivity of spending on ART to relative risk of prevalent infections and relative risk of incident infections


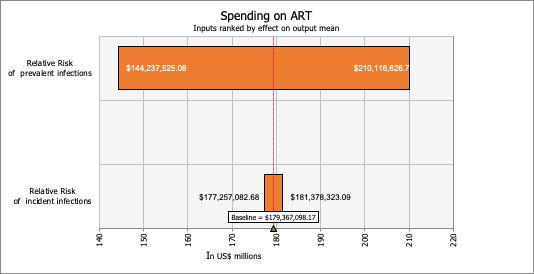


Figure 10: Spider Diagram - Sensitivity of spending on ART to relative risk of prevalent infections and relative risk of incident infections


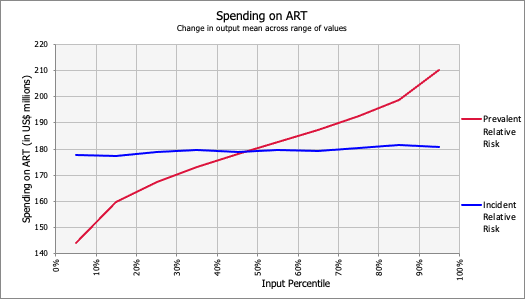


Table 12: Sensitivity of spending on ART to parameter uncertainty

| *Rank* | *Name* | *Range of Mean* |
| --- | --- | --- |
| *1* | *Relative risk (RR) due to prevalent HSV-2 infections* | *$144,291,971 - $210,075,984* |
| *2* | *Relative risk (RR) due to incident HSV-2 infections* | *$ 177,605,776 - $181,260,757* |

Figure 11: Tornado Diagram - Sensitivity of wage losses in HIV patients to the proportion employed, absent days when not on ART, and absent days when on ART.


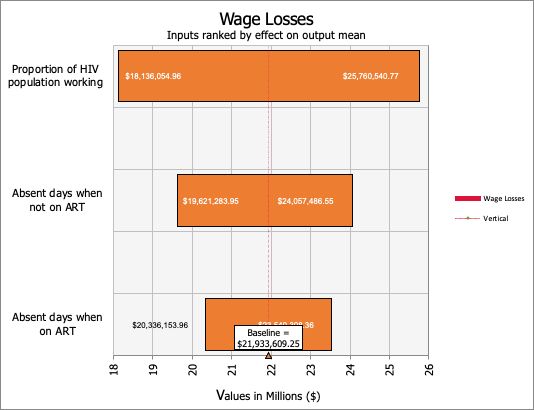


Figure 12: Spider Diagram - Sensitivity of wage losses in HIV patients to the proportion employed, absent days when not on ART, and absent days when on ART.


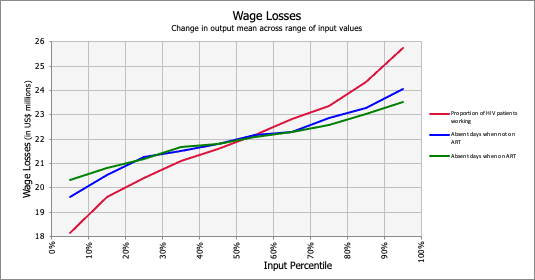


Table 13: Sensitivity of wage losses in HIV patients to parameter uncertainty

| *Rank* | *Name* | *Range of Mean* |
| --- | --- | --- |
| *1* | *Proportion of HIV patients who are employed* | *US$18,136,054 - US$25,760,540* |
| *2* | *Absent days when not on ART* | *US$19,621,283- US$24,057,486* |
| *3* | *Absent days when on ART* | *US$20,336,153-US$23,540,308* |
