# Supplementary material for: Estimated economic burden of genital herpes and HIV attributable to herpes simplex virus type 2 infections in 90 low- and middle-income countries: A modeling study
Source: PLoS Med. 2022 Dec 15;19(12):e1003938. doi: 10.1371/journal.pmed.1003938 (PMC9754187; doi:10.1371/journal.pmed.1003938)
Supplement: S1 Checklist — (DOCX) [file pmed.1003938.s003.docx]

**Estimated economic burden of genital herpes and HIV attributable to herpes simplex virus type-2 infections in 90 low- and middle-income countries: A modeling study**

**Consolidated Health Economic Evaluation Reporting Standards (CHEERS) guideline (S1 Checklist)**

Sachin SILVA^1, 2^, Houssein H AYOUB^3^, Christine JOHNSTON^4,5^, Rifat ATUN^1^, Laith J ABU-RADDAD^6,7,8^

1. Harvard TH Chan School of Public Health, Harvard University

Boston, MA Massachusetts, United States of America

1. University of California, San Francisco, Institute for Global Health Sciences,

San Francisco, California, United States of America

1. Mathematics Program, Department of Mathematics, Statistics, and Physics, College of Arts and Sciences, Qatar University

Doha, Qatar

1. Department of Medicine, University of Washington

, Seattle, Washington, United States of America

1. Vaccine and Infectious Diseases Division, Fred Hutchinson Cancer Research Center Eastlake Building, Day Campus,., Seattle, Washington, United States of America
2. Infectious Diseases Epidemiology Group, Weill Cornell Medicine – Qatar

Doha, Qatar

1. World Health Organization Collaborating Centre for Disease Epidemiology Analytics on HIV/AIDS, Sexually Transmitted Infections, and Viral Hepatitis, Weill Cornell Medicine – Qatar

Doha, Qatar

1. Department of Population Health Sciences, Weill Cornell Medicine, Cornell University,

New York, New York, United States of America

***** sas7443@mail.harvard.edu

| **Section or item** | **Item number** | | **CHEERS recommendation** | **Reported on page number and line number** | |
| --- | --- | --- | --- | --- | --- |
| **Title and abstract** | | | | | |
| Title | | 1 | Identify the study as an economic evaluation or use more specific terms such as “cost-effectiveness analysis”, and describe the interventions compared. | | Not applicable. We have identified the study as a modelling study. |
| Abstract | | 2 | Provide a structured summary of objectives, perspective, setting, methods (including study design and inputs), results (including base case and uncertainty analyses), and conclusions. | | Abstract section |
| **Introduction** | | | | | |
| Background and objectives | | 3 | Provide an explicit statement of the broader context for the study. | | Manuscript Introduction paragraph 1 |
|  |  |  | Present the study question and its relevance for health policy or practice decisions. | | Manuscript Introduction paragraph 2 Manuscript Introduction paragraph 1 |
| **Methods** | | | | | |
| Target population and subgroups | | 4 | Describe characteristics of the base case population and subgroups analysed, including why they were chosen. | | Manuscript Methods paragraph 1 |
| Setting and location | | 5 | State relevant aspects of the system(s) in which the decision(s) need(s) to be made. | | Not applicable |
| Study perspective | | 6 | Describe the perspective of the study and relate this to the costs being evaluated. | | Supplementary Appendix 1 Section 1 |
| Comparators | | 7 | Describe the interventions or strategies being compared and state why they were chosen. | | Not applicable |
| Time horizon | | 8 | State the time horizon(s) over which costs and consequences are being evaluated and say why appropriate. | | Manuscript Methods paragraph 1 |
| Discount rate | | 9 | Report the choice of discount rate(s) used for costs and outcomes and say why appropriate. | | Manuscript Results paragraph 10 |
| Choice of health outcomes | | 10 | Describe what outcomes were used as the measure(s) of benefit in the evaluation and their relevance for the type of analysis performed. | | Not applicable. Not an effectiveness study |
| Measurement of effectiveness | | 11a | *Single study-based estimates:*Describe fully the design features of the single effectiveness study and why the single study was a sufficient source of clinical effectiveness data. | | Not applicable |
|  |  | 11b | *Synthesis-based estimates*: Describe fully the methods used for identification of included studies and synthesis of clinical effectiveness data. | | Not applicable |
| Measurement and valuation of preference based outcomes | | 12 | If applicable, describe the population and methods used to elicit preferences for outcomes. | | Not applicable |
| Estimating resources and costs | | 13a | *Single study-based economic evaluation:* Describe approaches used to estimate resource use associated with the alternative interventions. Describe primary or secondary research methods for valuing each resource item in terms of its unit cost. Describe any adjustments made to approximate to opportunity costs. | | Not applicable |
|  |  | 13b | *Model-based economic evaluation:*Describe approaches and data sources used to estimate resource use associated with model health states. Describe primary or secondary research methods for valuing each resource item in terms of its unit cost. Describe any adjustments made to approximate to opportunity costs. | | Manuscript Methods paragraphes 1-8 |
| Currency, price date, and conversion | | 14 | Report the dates of the estimated resource quantities and unit costs. Describe methods for adjusting estimated unit costs to the year of reported costs if necessary. Describe methods for converting costs into a common currency base and the exchange rate. | | Manuscript Methods paragraph 8 |
| Choice of model | | 15 | Describe and give reasons for the specific type of decision-analytical model used. Providing a figure to show model structure is strongly recommended. | | Supplementary Appendix 1 section 3B (natural history model).  Supplementary Appendix 1 section 5A (epidemiological model).  Supplementary Appendix 1 section 5B (economic model). |
| Assumptions | | 16 | Describe all structural or other assumptions underpinning the decision-analytical model. | | Supplementary Appendix 1 section 3B (natural history model).  Supplementary Appendix 1 section 5A (epidemiological model).  Supplementary Appendix 1 section 5B (economic model).  Supplementary Appendix 1 section 7 (sensitivity analysis). |
| Analytical methods | | 17 | Describe all analytical methods supporting the evaluation. This could include methods for dealing with skewed, missing, or censored data; extrapolation methods; methods for pooling data; approaches to validate or make adjustments (such as half cycle corrections) to a model; and methods for handling population heterogeneity and uncertainty. | | Supplementary Appendix 1 section 5B |
| **Results** | | | | | |
| Study parameters | | 18 | Report the values, ranges, references, and, if used, probability distributions for all parameters. Report reasons or sources for distributions used to represent uncertainty where appropriate. Providing a table to show the input values is strongly recommended. | | Supplementary Appendix 1 section 7 |
| Incremental costs and outcomes | | 19 | For each intervention, report mean values for the main categories of estimated costs and outcomes of interest, as well as mean differences between the comparator groups. If applicable, report incremental cost-effectiveness ratios. | | Not applicable |
| Characterising uncertainty | | 20a | *Single study-based economic evaluation:* Describe the effects of sampling uncertainty for the estimated incremental cost and incremental effectiveness parameters, together with the impact of methodological assumptions (such as discount rate, study perspective). | | Not applicable |
|  |  | 20b | *Model-based economic evaluation:*Describe the effects on the results of uncertainty for all input parameters, and uncertainty related to the structure of the model and assumptions. | | Supplementary Appendix 1 section 7 |
| Characterising heterogeneity | | 21 | If applicable, report differences in costs, outcomes, or cost-effectiveness that can be explained by variations between subgroups of patients with different baseline characteristics or other observed variability in effects that are not reducible by more information. | | Not applicable |
| **Discussion** | | | | | |
| Study findings, limitations, generalisability, and current knowledge | | 22 | Summarise key study findings and describe how they support the conclusions reached. Discuss limitations and the generalisability of the findings and how the findings fit with current knowledge. | | Manuscript Results paragraphs 1-8 |
| **Other** | | | | | |
| Source of funding | | 23 | Describe how the study was funded and the role of the funder in the identification, design, conduct, and reporting of the analysis. Describe other non-monetary sources of support. | | Manuscript Role of the Funding Source section |
| Conflicts of interest | | 24 | Describe any potential for conflict of interest of study contributors in accordance with journal policy. In the absence of a journal policy, we recommend authors comply with International Committee of Medical Journal Editors recommendations. | | Manuscript Competing Interests Statement section |
